# A control theoretic three timescale model for analyzing energy management in mammalian cancer cells

**DOI:** 10.1016/j.csbj.2020.12.019

**Published:** 2020-12-29

**Authors:** Abhijit Dasgupta, Abhisek Bakshi, Nirmalya Chowdhury, Rajat K. De

**Affiliations:** a Machine Intelligence Unit, Indian Statistical Institute, 203 B.T. Road, Kolkata 700108, India; b Department of Data Science, School of Interdisciplinary Studies, University of Kalyani, Kalyani, Nadia 741235, West Bengal, India; c Department of Computer Science & Engineering, Jadavpur University, Kolkata 700032, India; d Department of Information Technology, Bengal Institute of Technology, Basanti Highway, Kolkata 700150, India

**Keywords:** Pathway integration, Timescale, MIMO, Support vector regression, Genetic algorithm, Warburg effect

## Abstract

Interaction among different pathways, such as metabolic, signaling and gene regulatory networks, of cellular system is responsible to maintain homeostasis in a mammalian cell. Malfunctioning of this cooperation may lead to many complex diseases, such as cancer and type 2 diabetes. Timescale differences among these pathways make their integration a daunting task. Metabolic, signaling and gene regulatory networks have three different timescales, such as, ultrafast, fast and slow respectively.

The article deals with this problem by developing a support vector regression (SVR) based three timescale model with the application of genetic algorithm based nonlinear controller. The proposed model can successfully capture the nonlinear transient dynamics and regulations of such integrated biochemical pathway under consideration. Besides, the model is quite capable of predicting the effects of certain drug targets for many types of complex diseases. Here, energy and cell proliferation management of mammalian cancer cells have been explored and analyzed with the help of the proposed novel approach. Previous investigations including *in silico*/*in vivo*/*in vitro* experiments have validated the results (the regulations of glucose transporter 1 (glut1), hexokinase (HK), and hypoxia-inducible factor-1α (HIF-1α) among others, and the switching of pyruvate kinase (M2 isoform) between dimer and tetramer) generated by this model proving its effectiveness. Subsequently, the model predicts the effects of six selected drug targets, such as, the deactivation of transketolase and glucose-6-phosphate isomerase among others, in the case of mammalian malignant cells in terms of growth, proliferation, fermentation, and energy supply in the form of adenosine triphosphate (ATP).

## Highlights

• Developed a three timescale model of integrated biochemical pathway.
• Simulated “Warburg Effect” using support vector regression and genetic algorithm.
• Identified rational drug targets using nonlinear controller.
• Explored energy and cell proliferation management of cancer cells.
• Validated the model by previous *in vivo*/*in vitro*/*in silico* experiments.

## Graphical abstract

**Figure:**
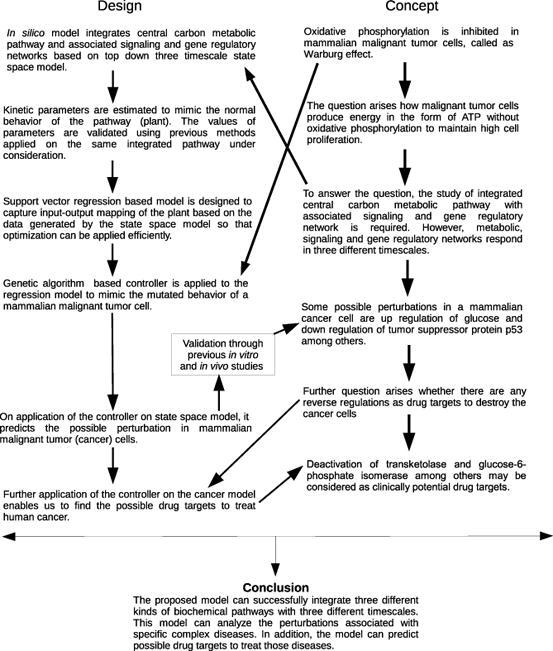

## 1 Introduction

Cellular decision making and responses are orchestrated by a set of complex biochemical pathways/networks. Broadly speaking, biochemical pathways/networks can be categorized as metabolic pathways, gene regulatory networks (GRNs), and signaling pathways. A metabolic pathway is a coherent set of biochemical reactions catalyzed by a number of enzymes. It helps a living organism to transform an initial (source) compound into a final (target) compound and energy. On the other hand, fundamental information processing and control mechanisms in a cell are performed by GRNs. Regulatory genes code for proteins that activate or inhibit the expression of other genes. Thus, a complex web of interactions, called a GRN, in terms of activation and inhibition of genes, is formed. Moreover, signaling pathways contain a series of specific actions in a cell in which a signal is passed from the environment and accordingly the cell responds. These pathways are of diverse nature and are interacting with one another. They also form a hierarchy. For example, an entire organism can be thought of as a huge network of interacting organs, each of which, in turn, is a network of interacting tissues. A tissue is a collection of interacting cells performing similar functions. A cell may be considered as a huge network of interacting components to constitute aforesaid biochemical networks. Moreover, the influence of environment and other factors on enzymes or gene regulation needs to be considered to make a study on how an organism is responsive to environmental changes. Therefore, it is necessary to focus not only on individual processes or pathways but also on their integration. Fig. S1 in Supplementary material depicts the interaction among metabolic, signaling and gene regulatory networks related to central carbon metabolism in a mammalian cell. Attempts to elucidate such huge biochemical networks on the structural/ functional basis face the problem of combinatorial explosion. There exist several investigations on modeling each of these pathways individually [1], [2], [3], [4], [5], [6], [7], [8], [9]. However, the study on the integration of gene regulation, metabolism, and signaling events (pathways) is not much.

Among several approaches one of the most common is to explore metabolic pathway is Flux Balance Analysis (FBA) [10], [11]. FBA can analyze only steady-state response of a system rather transient response. However, Metabolic Control Analysis [12] overcomes the limitation of FBA. Yet, it does not provide any supervisory controller that can regulate the enzyme/metabolite concentration to meet some particular requirements of a cell. Recently, we have developed a control theoretic approach to solve this problem [13], [14], [15]. Some investigations on crosstalk mechanism [16] and identifying properties [17], [18] of signaling networks have also been developed. Since the activation of regulatory mechanism depends on corresponding gene expression level thus any GRN can be modeled mathematically by coupled ordinary differential equations (ODEs), Boolean network or Bayesian network [19].

Response time of the interconnected complex subsystems may vary from each other. In other words, they can be treated as complex subsystems of different timescales. Biochemical pathways, such as metabolic, signaling and regulatory networks show this kind of phenomena. Timescale differences among metabolic, signaling and gene regulatory networks make their integration a daunting task. Several researchers have tried to model the dynamic behavior of a system by stoichiometric reconstruction of integrated biochemical pathways [20]. Here, the authors have considered two different timescales. The metabolic and signaling networks have been considered in fast timescale, whereas GRN has been considered in slow timescale. However, the metabolic, signaling and gene regulatory networks participate in three different timescales. Previous investigation [21] show that proteins in *Escherichia coli* take seconds to minutes to express. It will be longer in the case of mammalian systems. On the other hand, metabolite concentrations can respond in seconds to microseconds [22]. Gene regulatory events take minutes to hours [20]. Thus, integration of these biochemical pathways becomes a three timescale problem, where metabolic, signaling and gene regulatory networks are ultrafast, fast and slow respectively.

In this study, we develop a three timescale multiple input and multiple output (MIMO) model based on support vector regression (SVR) and genetic algorithm based controller to simulate the dynamic behavior of integrated signaling, metabolic and gene regulatory networks specifically responsible for mammalian carbon metabolism in normal and cancer cells. Here, the metabolic, signaling and GRN have been mathematically modeled by sets of ODEs with proper three timescale selection, feedback, allosteric effects and perturbation. In this context, we have collected all pathway information under consideration from KEGG database [23] and literature. Finally, we have developed a genetic algorithm (GA) based controller to drive the change of concentrations/expression levels of certain metabolites/proteins/genes with respect to time in a desired fashion. Thus, the proposed model can also predict the possible effects of certain drug targets of specific diseases through GA controller.

**Fig. 1:**
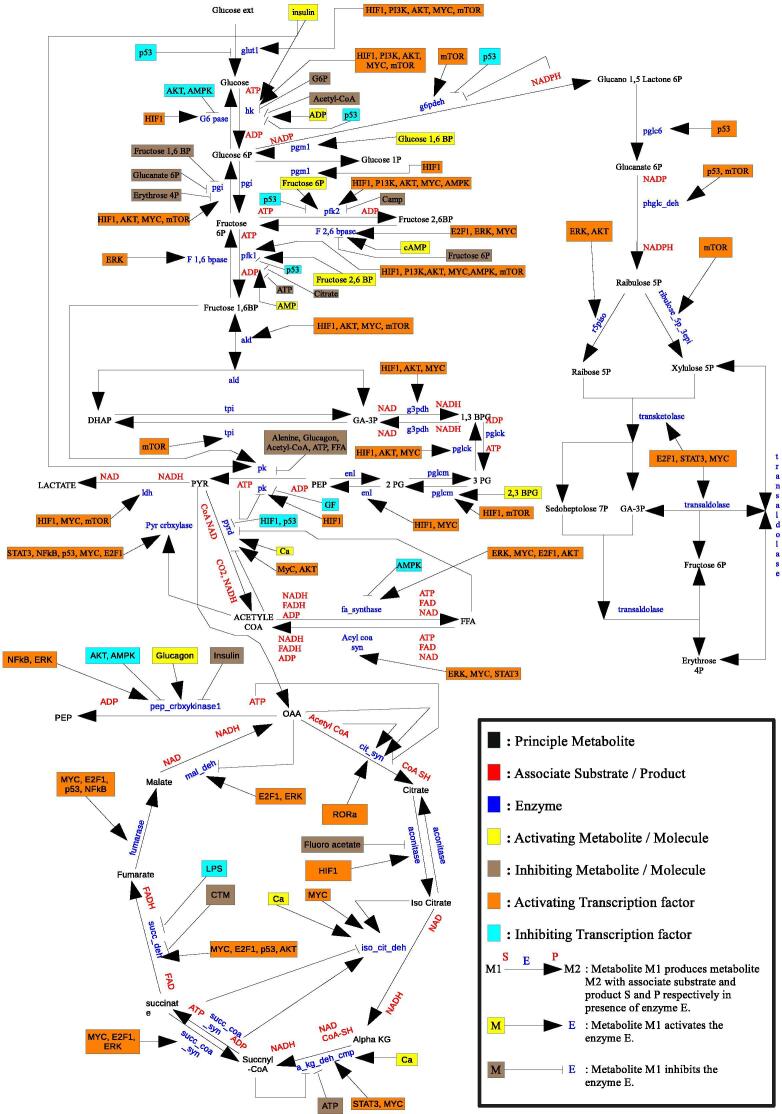
Integrated metabolic, signaling and gene regulatory networks associated with central carbon metabolism of mammalian cells.

The central carbon metabolic (CCM) network comprises glycolysis, tricarboxylic acid (TCA) cycle, pentose phosphate pathway (PPP) and free fatty acid (FFA) metabolism. Glycolysis consumes glucose to produce pyruvate and energy in the form adenosine triphospate (ATP), although most of the energy is generated from TCA cycle taking pyruvate as input. Here, PPP is involved in macromolecular synthesis including reduced nicotinamide adenine dinucleotide phosphate (NADPH) and ribose 5P. FFA is formed by acetyl CoA of TCA cycle. However, a mammalian tumor cell slows down oxidative phosphorylation by inhibiting TCA cycle activities and produces abnormal amount of lactate. This effect is called “Warburg effect” [24], [25] which may lead to cancer [26]. In this context, we have analyzed the energy and cell proliferation management of CCM pathway along with corresponding signaling and gene regulatory networks in mammalian cancer cells. Here, we have considered the interactions among different enzymes/proteins, transcription factors and genes associated with central carbon metabolism to capture the cellular dynamics during normal and malignant conditions in mammalian cells. These detailed interactions can be found in Supplementary Tables S1–S8 (abbreviations of different molecules can be found in Supplementary Table S10). Fig. 1 depicts the integrated metabolic, signaling and gene regulatory networks associated with central carbon metabolism. We also have analyzed the possible effects of six drug targets, such as deactivation of pyruvate kinase, glucose-6-phosphate dehydrogenase, transketolase, ribose 5P isomerase, glucose-6-phosphate isomerase and finally activation of pyruvate kinase, on mammalian malignant cells.

In accordance with previous investigations including some *in vivo* and *in vitro* experiments [24], [27], [26], [28], [29], [30], [31], [32], [33], [34], [35], [36], [37], [38], [39], [40], [41], [42], [43], [44], [45], [25], [46], [47], [48], [49], [50], [51], [52], [53], [54], [55], [56], [57], [58], [59], [60], [61], [62], [63], [64], [65], the simulation results also suggest that abnormal over expressions of certain proteins and genes depicted in Table 3 play significant roles to maintain growth, proliferation and energy supply in mammalian cancer cells to survive. Under expressions of prolyl-hydroxylases (PHD) and tumor suppressing protein p53 are also important in this context. Besides, switching of pyruvate kinase (M2 isoform) to its inactive dimer or active tetrameric form helps to synthesize energy in the form of ATP, and macromolecular precursors for cell growth and proliferation according to the requirements of mammalian malignant cells.

**Table 3:** Illustrating significant regulations of different metabolites, transcription factors and genes in mammalian cancel cells compared to that in normal ones.

Network type	Name of the molecules	Significant observation in cancer cells compared to normal ones	References for validation
Metabolic	ATP	Over production	[46], [47], [48], [49]
	Ribose-5P	Over production	[51], [52], [53]
	Lactate	Over production	[109], [25], [105], [107]
	GA3P	Over production	[46], [47], [48], [49]
	Pyruvate	Over production	[46], [47], [48], [49]
	Glucose consumption/G6P production	Over production	[46], [107], [105]
	Glucose production	Less production	[46]
	PEP	Less production	[46], [47], [48], [49]
	Fructose 6P	Less production	[96]
	NADH	Less production	[50], [103]

Signaling	HIF-1α	Over expressed	[28], [54]
	P13K	Over expressed	[31], [32], [33], [55]
	AKT	Over expressed	[31], [32], [33], [56], [57]
	mTOR	Over expressed	[31], [32], [33], [55]
	MYC	Over expressed	[34], [35], [58], [59], [65]
	ERK	Over expressed	[38], [39], [40]
	STAT3	Over expressed	[41], [42], [60], [61], [62]
	NF-κB	Over expressed	[39], [43], [44], [63]
	p53	Under expressed	[36]
	PHD	Under expressed	[29], [30]

Gene regulatory	HK	Over expressed	[27], [45], [106]
	Glut 1	Over expressed	[27], [45], [106]
	LDH	Over expressed	[109], [25], [105]
	PFK 1	Over expressed	[46], [104]
	PFK 2	Over expressed	[46]
	Glyceraldehyde-3-phosphate dehydrogenase	Over expressed	[46]
	Pyruvate kinase	Switching alternatively from low to high and vice versa	[47], [48], [49], [104], [64]
	Glucose-6-phosphate dehydrogenase	Over expressed	[51], [52], [53]
	Phospho-gluco dehydrogenase	Over expressed	[51], [52], [53]
	Ribose 5P isomerase	Over expressed	[51], [52], [53]
	Transketolase	Over expressed	[51], [52], [53]
	Transaldolase	Over expressed	[51], [52], [53]

We have also compared the proposed model with the following theoretical models. In this context, FBA based model [66] has depicted a similar set of malfunctioning enzymes as identified by the proposed model in cancer cells for its growth. However, FBA based model does not involve transient analysis of the behavior of molecules in cancer pathways. Previous differential equation and optimization-based models [67], [68], [69] have also depicted similar mutation as depicted by the proposed model in cancer cells. However, these models do not consider the three timescale nature of the integrated CCM pathways. Besides, they have not explored the outcome of probable drug targets to control energy metabolism in cancer cells.

Here the proposed model is able to predict that deactivation of glucose-6-phosphate dehydrogenase and ribose 5P isomerase may slow down growth and proliferation of cancer cells. However, glucose-6-phosphate dehydrogenase deactivation may not be able to reduce fermentation and energy supply in malignant cells. On the other hand, though ribose 5P isomerase deactivation may inhibit cell fermentation, it may not get success to stop energy supply in mammalian cancer cells. In this context, deactivation of transketolase and glucose-6-phosphate isomerase may be considered as potential drug targets to resist cell growth, fermentation and proliferation as well as energy supply in human cancer cells. Activation of pyruvate kinase (M2 isoform) may also reduce cancer progression in terms of cell growth, proliferation and fermentation. Besides, it may fail to decrease ATP production in tumor cells. However, deactivation of pyruvate kinase may be a poor choice as a drug target for cancer therapy.

In summary, the proposed novel model can tackle three timescale nature of any integrated biochemical pathway comprising metabolic, signaling and gene regulatory networks. Subsequently, it can capture the nonlinear transient dynamics of the integrated network of both normal and perturbed human cells. This has been validated appropriately by some other methods/results [70], [71], [72], [66], [67], [68], [69]. Moreover, using proposed GA controller, effects of drug targets on diseased cells have been explored and analyzed. Thus, it can help to find out a novel therapeutic goal for complex diseases, such as cancer and type 2 diabetes.

## 2 Method

**Fig. 2:**
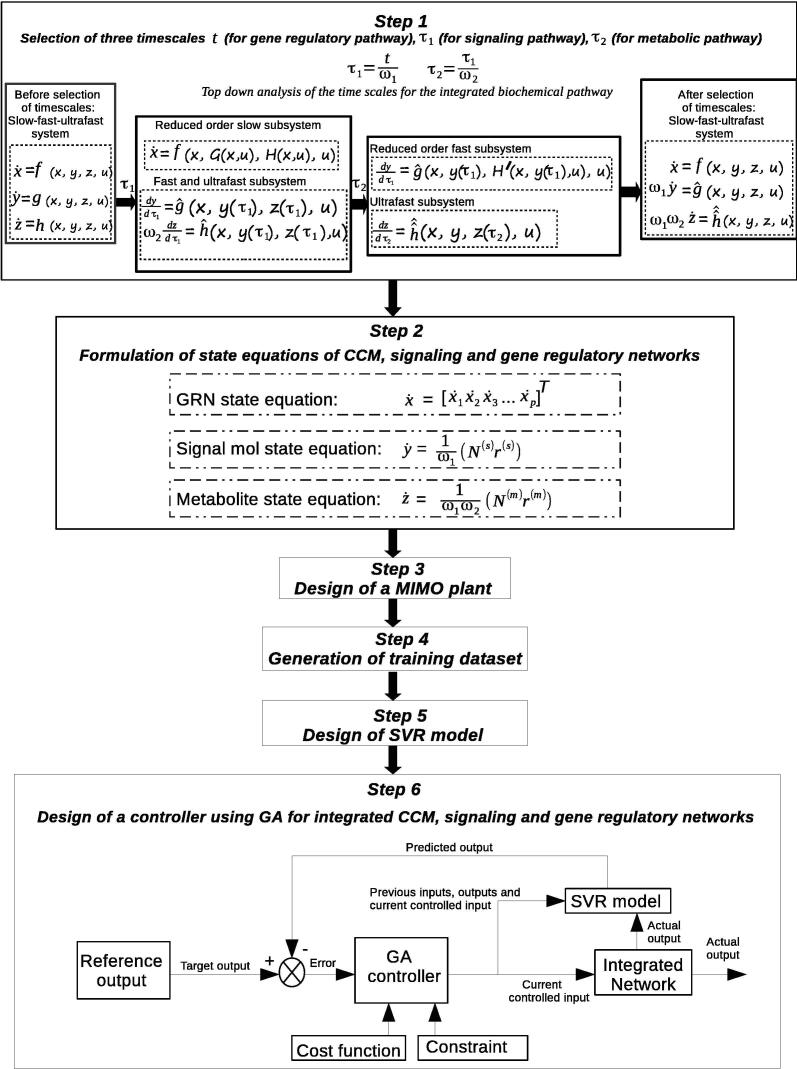
Flowchart of the entire methodology: Here slow, fast and ultrafast subsystems correspond to gene regulatory, signaling and metabolic pathways respectively.

Here, we describe the proposed methodology for developing the model that will mimic the behavior of an integrated pathway system. The methodology involves six steps. At first, timescales of metabolic, signaling and gene regulatory networks have been selected, and the basic dynamics of the integrated pathway system has been described. Then, we have formulated the state equations for the integrated pathway. In the third step, an appropriate MIMO plant has been developed by tuning its parameters. Subsequently, training dataset has been generated from the appropriately perturbed MIMO plant/system. Thereafter, SVR model is developed to approximate the MIMO plant. Finally, GA controller is applied on the plant as a model predictive controller to capture nonlinear transient cellular dynamics. Fig. 2 depicts the flowchart of the entire methodology. Here we have considered central carbon metabolism related metabolic (glycolysis, TCA cycle and pentose phosphate pathways as well as FFA synthesis/consumption), signaling and gene regulatory networks throughout the entire methodology. All the mathematical symbols used in this article are defined in Table 1. However, each of these symbols has again been described whenever it appears first.

**Table 1:** Different mathematical symbols and their description.

Symbols	Description
x,y,z and u	Vectors representing gene expression levels, protein/signaling molecule expression levels, concentration of metabolites and external inputs (Supplementary Table S11) respectively.
p,s,m and *c*	Dimension of x,y,z and u respectively.
i,j,k and *l*	Index for gene, signaling molecule, metabolite and external inputs respectively.
$t,\tau_{1}$ and $\tau_{2}$	Timescales for gene regulatory, signaling and metabolic networks respectively.
$\overset{˙}{x},\overset{˙}{y}$ and $\overset{˙}{z}$	Derivatives of x,y and z respectively, with respect to time *t*.
$\omega_{1}$ and $\omega_{2}$	Slow to fast and Fast to ultrafast timescale ratios.
f(.),g(.) and h(.)	Activities corresponding to gene regulatory, signaling and metabolic networks respectively.
g^(.) and h^(.)	Fast and ultrafast subsystems with respect to $\tau_{1}$.
h^^(.)	Ultrafast subsystem with respect to $\tau_{2}$.
G(.) and H(.)	Quasi-steady state equilibrium of fast and ultrafast subsystems.
$H^{\prime }(.)$	Quasi-steady state equilibrium of ultrafast subsystem.
$e_{i},b_{i}$ and $d_{i}$	Expression, basal production and decay rates respectively for $i^{\mathrm{th}}$ gene.
$K_{{\mathrm{ij}}^{\prime }}^{\left(g\right)}$	Binding rate constant for $j^{\prime \mathrm{th}}$ transcription factor required to express $i^{\mathrm{th}}$ gene.
$F_{\mathrm{ik}}^{\left(g\right)},F_{\mathrm{il}}^{\left(g\right)}$ and $F_{{\mathrm{ij}}^{\prime \prime }}^{\left(g\right)}$	Binding constants for $i^{\mathrm{th}}$ gene corresponding to $k^{\mathrm{th}}$ metabolite, $l^{\mathrm{th}}$ external input and $j^{\prime \prime \mathrm{th}}$ signaling molecule/transcription factor respectively.
$N^{\left(s\right)},r^{\left(s\right)}$ and *q*	Interaction matrix, interaction rate vector and number of interactions for signal transduction pathway.
$r_{\sigma }^{\left(s\right)}$	$\sigma^{\mathrm{th}}$ element of $r^{\left(s\right)}$.
$K_{{\mathrm{jj}}^{\prime }}^{\left(s\right)}$	Strength of binding constant of $j^{\prime \mathrm{th}}$ signaling molecule with $j^{\mathrm{th}}$ signaling molecule.
$F_{\mathrm{jk}}^{\left(s\right)},F_{\mathrm{jl}}^{\left(s\right)}$ and $F_{{\mathrm{jj}}^{\prime \prime }}^{\left(s\right)}$	Binding constants for $j^{\mathrm{th}}$ signaling molecule corresponding to $k^{\mathrm{th}}$ metabolite, $l^{\mathrm{th}}$ external input and $j^{\prime \prime \mathrm{th}}$ signaling molecule respectively.
$N^{\left(m\right)},r^{\left(m\right)}$ and *n*	Stoichiometric matrix, metabolic flux vector and number of reactions for metabolic pathway.
$r_{\rho }^{\left(m\right)}$	$\rho^{\mathrm{th}}$ element of $r^{\left(m\right)}$.
$K_{\rho }^{\left(m\right)}$ and $K_{\rho }^{M^{\left(m\right)}}$	The kinetic rate constant and Michaelis Menten constant respectively for $\rho^{\mathrm{th}}$ metabolic reaction.
$F_{{\mathrm{kk}}^{\prime \prime }}^{\left(m\right)}$ and $F_{\mathrm{kl}}^{\left(m\right)}$	Feedback constants for $k^{\mathrm{th}}$ metabolite corresponding to $k^{\prime \prime \mathrm{th}}$ metabolite and $l^{\mathrm{th}}$ external input respectively.
θ,τ,v,d,D and ϕ	Time, number of past inputs, input vector for SVR, dimension of input vector v, dataset and number of samples respectively.
μ and *a* or *b*	Index for output and v respectively.
$d^{\prime },F,w_{\mu },\delta (.)$ and $\beta_{\mu }$	Dimension of projected feature space, projected feature space, the orientation of the hyperplane in *F*, mapping from *d* dimensional input space to $d^{\prime }$ dimensional transformed feature space *F* and the bias respectively.
γμ,γ^μ,Pμ and $Q_{\mu }$	$\mu^{\mathrm{th}}$ element of actual output, $\mu^{\mathrm{th}}$ element of predicted output, convex optimization problem and quadratic programming problem respectively.
$\varepsilon_{\mu },\varphi_{a},\varphi_{a}^{*}$ and C	Tolerable error, slack variables and a trade-off respectively.
$\zeta_{a},\zeta_{a}^{*},\zeta_{b}$ and $\zeta_{b}^{*}$	Lagrange multipliers.
$\alpha_{b}$	$\left(\zeta_{b}-\zeta_{b}^{*}\right)$.
*C* and *G*	Number of chromosomes and generations respectively.

### 2.1 Selection of three timescales and the basic dynamics of an integrated pathway system

In an integrated biochemical pathway system, involving metabolic, signaling and gene regulatory networks, the rate of a metabolic reaction is controlled by an enzyme/protein, which is originated from an expressed gene participating in a gene regulatory network. The expression of this gene is in turn regulated by one or more transcription factors (proteins) which are expressed/activated by signaling molecules in a cascade of signal transduction pathway.

Let $x_{i}$ be the expression level of $i^{\mathrm{th}}\;\;(i=1,2,\ldots ,p)$ gene generating $i^{\mathrm{th}}$ enzyme involved in a certain metabolic pathway. Similarly, let $y_{j}$ be the expression level of $j^{\mathrm{th}}\;\;(j=1,2,\ldots ,s)$ protein/signaling molecule, involved in a signal transduction pathway. Let $z_{k}$ be the concentration of $k^{\mathrm{th}}\;\;(k=1,2,\ldots ,m)$ metabolite involved in the metabolic pathway under consideration. Besides, there are *c* external inputs $u_{l}\;\;(l=1,2,\ldots ,c)$ applied to the system, depicting perturbation from environment and other pathways to the current integrated pathway system. The terms x,y,z and u are p,s,m and *c* dimensional vectors, respectively, for the pathway system.

Thus the proposed nonlinear state space model for the integrated pathway can be defined as

(1) $$\overset{˙}{x}=f(x,y,z,u)$$

(2) $$\overset{˙}{y}=g(x,y,z,u)$$

(3) $$\overset{˙}{z}=h(x,y,z,u)$$

Here f(.),g(.) and h(.) represent activities corresponding to gene regulatory, signaling and metabolic networks. The time (*t*) derivatives of x,y and z are represented by $\overset{˙}{x},\;\overset{˙}{y}$ and $\overset{˙}{z}$ respectively. State variable x is slow, whereas y and z are fast and ultrafast state variables respectively. The symbols $\omega_{1}$ and $\omega_{2}$ correspond to slow to fast and fast to ultrafast timescale ratios. Here, $\omega_{1}$ and $\omega_{2}$ are small positive quantities. According to previous investigation [21], proteins in *Escherichia coli* take minutes to express. It will be longer in the case of mammalian systems. However, metabolite concentrations can alter in seconds [22]. On the other hand, gene regulatory events take hours [20]. Based on these assumptions, we have considered $\omega_{1}=1/60$ and $\omega_{2}=1/60$, and thereby the stretched timescales as $\tau_{1}$ = $t/\omega_{1}$ and $\tau_{2}$ = $\tau_{1}/\omega_{2}$ = $t/(\omega_{1}\omega_{2})$. Thus the timescales for gene regulatory, signaling and metabolic networks are $t,\;\tau_{1}$ and $\tau_{2}$ respectively.

We now obtain the fast (g^(.)) and ultrafast (h^(.)) subsystems corresponding to signaling and metabolic networks from the top down timescale decomposition [73], [74] as

(4) ω1y˙=dydτ1=g^(x,y(τ1),z(τ1),u)

and

(5) ω1ω2z˙=ω2dzdτ1=h^(x,y(τ1),z(τ1),u)

Here, $y(\tau_{1})$ and $z(\tau_{1})$ are instantaneous y and z-values, respectively, in $\tau_{1}$ timescale. Moreover, considering $\omega_{1}\to 0$, if we solve Eqs. (4), (5) algebraically for y and z, we get

(6) y=G(x,u)

and

(7) z=H(x,u)

Two vector functions G(x,u) and H(x,u) represent the quasi-steady state equilibrium [75] of the fast (g^(.)) and ultrafast (h^(.)) subsystems. Moreover, being a slow state variable in timescale t,x does not change significantly with respect to fast timescale $\tau_{1}$. Thus, the reduced slow gene regulatory subsystem can be defined using top-down timescale decomposition [73], [74] as

(8) $$\overset{˙}{x}=f(x,G(x,u),H(x,u),u)$$

Again, the fast and ultrafast subsystems corresponding to signaling and metabolic networks can be treated as a two timescale system. The ultrafast subsystem corresponding to metabolic network (h^^(.)) is given by

(9) ω2dzdτ1=dzdτ2=h^^(x,y,z(τ2),u)

Now, if we solve Eq. (9) algebraically considering $\omega_{2}\to 0$, we can get

(10) 0=h^^(x,y,z,u)⇒z=H′(x,y(τ1),u)

The term $H^{\prime }$(x,y($\tau_{1}$),u) represents the quasi steady state equilibrium [75] of the ultrafast subsystem h^^(.). Here, x and y do not change appreciably with respect to $\tau_{2}$. Thus, the reduced fast signaling network can be defined as

(11) dydτ1=g^(x,y(τ1),H′(x,y(τ1),u),u)

Thus,

(12) $$\overset{˙}{x}=f(x,y,z,u)$$

(13) y˙=1ω1g^(x,y,z,u)

(14) z˙=1ω1ω2h^^(x,y,z,u)

Here we can determine the value of u, in terms of x,y and z, considering the equilibrium state of the system.

### 2.2 Formulation of state equations of integrated biochemical pathways

Here we have shown how state equations for gene regulatory, signaling and metabolic networks are formed. Let a gene $x_{i}$ be expressed by $s^{\prime }$ ($1⩽s^{\prime }<s$) transcription factors $y_{j^{\prime }}$ ($j^{\prime }=1,\ldots ,s^{\prime }$) included in a signaling pathway. Thus, $\overset{˙}{x_{i}}$ can be written as

(15) $$\overset{˙}{x_{i}}=\left\{\begin{array}{cc} e_{i}+b_{i}-d_{i}, & \mathrm{if}\;\overset{s^{\prime }}{\underset{j^{\prime }=1}{\prod }}y_{j^{\prime }}>0\\ b_{i}-d_{i}, & \mathrm{if}\;\overset{s^{\prime }}{\underset{j^{\prime }=1}{\prod }}y_{j^{\prime }}=0 \end{array}\right)$$

The terms $e_{i}$ and $b_{i}$ represent the expression and basal production (of mRNA) rates [76] of $x_{i}$ respectively, whereas $d_{i}$ denotes the decay rate. The expression rate $e_{i}$ of a particular gene $x_{i}$ depends on the binding of many required transcription factors. Absence of any of those transcription factors, the expression of that particular gene is driven by its basal production and decay rate only. Now, $e_{i}$ can be defined as

(16) $$e_{i}=\overset{s^{\prime }}{\underset{j^{\prime }=1}{\prod }}K_{{\mathrm{ij}}^{\prime }}^{\left(g\right)}y_{j^{\prime }}$$

where $K_{{\mathrm{ij}}^{\prime }}^{\left(g\right)}$ is the binding rate constant for $j^{\prime \mathrm{th}}$ transcription factor required to express the gene $x_{i}$.

Let us consider $m^{\prime }$ ($1⩽m^{\prime }<m$) metabolites $z_{k}$ and $c^{\prime }$ ($1⩽c^{\prime }<c$) external inputs $u_{l}$ acting as the cofactors [77] to activate an $i^{\mathrm{th}}$ gene and $\prod_{j^{\prime }=1}^{s^{\prime }}y_{j^{\prime }}>0$. As a result, the expression rate $e_{i}$ of the gene $x_{i}$ is enhanced by a multiplication factor. Thus, $\overset{˙}{x_{i}}$ can be rewritten as

(17) $$\overset{˙}{x_{i}}=\overset{m^{\prime }}{\underset{k=1}{\prod }}(1+F_{\mathrm{ik}}^{\left(g\right)}z_{k})\overset{c^{\prime }}{\underset{l=1}{\prod }}(1+F_{\mathrm{il}}^{\left(g\right)}u_{l})\overset{s^{\prime }}{\underset{j^{\prime }=1}{\prod }}K_{{\mathrm{ij}}^{\prime }}^{\left(g\right)}y_{j^{\prime }}+b_{i}-d_{i}$$

Here $F_{\mathrm{ik}}^{\left(g\right)}$ and $F_{\mathrm{il}}^{\left(g\right)}$ represent the constants corresponding to $z_{k}$ and $u_{l}$ to take care of the relative strength of binding with $i^{\mathrm{th}}$ gene [13], [78], [14]. We have considered binding constants to be in [0,1). Zero binding constant signifies no binding corresponding to a molecule. Whereas, higher value of a binding constant indicates stronger binding corresponding to the molecule. On the other hand, if $m^{\prime }$ metabolites $z_{k},\;s^{''}$ ($1⩽s^{\prime \prime }<s$) signaling molecules/transcription factors $y_{j^{\prime \prime }}$ ($j^{''}\;\neq \;j^{'},\;j^{''}=1,\ldots ,s^{''}$) and $c^{\prime }$ external inputs $u_{l}$ slow down the activation of the $i^{\mathrm{th}}$ gene and $\prod_{1}^{s^{\prime }}y_{j^{\prime }}>0$, the expression rate $e_{i}$ is decreased by a fraction. Thus, Eq. (17) can be modified as

(18) $$\begin{array}{c} \overset{˙}{x_{i}}=\frac{\overset{s^{\prime }}{\underset{j^{\prime }=1}{\prod }}K_{{\mathrm{ij}}^{\prime }}^{\left(g\right)}y_{j^{\prime }}+b_{i}}{\overset{m^{\prime }}{\underset{k=1}{\prod }}(1+F_{\mathrm{ik}}^{\left(g\right)}z_{k})\overset{c^{\prime }}{\underset{l=1}{\prod }}(1+F_{\mathrm{il}}^{\left(g\right)}u_{l})\overset{s^{\prime \prime }}{\underset{j^{\prime \prime }=1}{\prod }}(1+F_{{\mathrm{ij}}^{\prime \prime }}^{\left(g\right)}y_{j^{\prime \prime }})}-d_{i}\\ \left(0<F_{\mathrm{ik}}^{\left(g\right)}z_{k},F_{\mathrm{il}}^{\left(g\right)}u_{l},F_{{\mathrm{ij}}^{\prime \prime }}^{\left(g\right)}y_{j^{\prime \prime }}<1\right) \end{array}$$

The term $F_{{\mathrm{ij}}^{\prime \prime }}^{\left(g\right)}$ represent the binding constant corresponding to $y_{j^{\prime \prime }}$ with $i^{\mathrm{th}}$ gene.

A signal transduction pathway can be defined by its interaction matrix $N^{\left(s\right)}$ which contains the information about interactions among the signaling molecules including transcription factors. If there are *q* interactions with initial rate vector $r^{\left(s\right)}$ (in timescale $\tau_{1}$) of dimension *q* involving *s* signaling molecules including transcription factors, the order of $N^{\left(s\right)}$ becomes s×q. It may be mentioned here that $r^{\left(s\right)}$ is analogous to a flux vector in a metabolic pathway. The ${\left(j,\sigma \right)}^{\mathrm{th}}$ element of $N^{\left(s\right)}$ becomes -1 if $j^{\mathrm{th}}$ signaling molecule participates in $\sigma^{\mathrm{th}}$ interaction. If $j^{\mathrm{th}}$ signaling molecule is produced/activated by $\sigma^{\mathrm{th}}$ interaction, the ${\left(j,\sigma \right)}^{\mathrm{th}}$ element of $N^{\left(s\right)}$ becomes +1.

Let a signaling molecule $y_{j}$ be activated by certain signaling molecules $y_{j^{\prime }}$ ($j^{\prime }\;\neq \;j$) in a signaling interaction with initial rate $r_{\sigma }^{\left(s\right)},\;\sigma^{\mathrm{th}}$ (σ=1,…,q) element of signaling interaction rate vector $r^{\left(s\right)}$. Consequently, the initial interaction rate $r_{\sigma }^{\left(s\right)}$ depends on the binding of such signaling molecules activating $y_{j}$. If some metabolites $z_{k}$, signaling molecules $y_{j^{\prime \prime }}$ ($j^{\prime \prime }\;\neq \;j^{\prime }$) and external inputs $u_{l}$ slow down the activation of $y_{j}$, a certain fraction can be multiplied with the initial interaction rate. Thus, we can write using modified mass action law [78] as

(19) $$r_{\sigma }^{\left(s\right)}=\frac{\overset{s^{\prime }}{\underset{j^{\prime }=1}{\prod }}K_{{\mathrm{jj}}^{\prime }}^{\left(s\right)}y_{j^{\prime }\neq j}}{\overset{m^{\prime }}{\underset{k=1}{\prod }}(1+F_{\mathrm{jk}}^{\left(s\right)}z_{k})\overset{c^{\prime }}{\underset{l=1}{\prod }}(1+F_{\mathrm{jl}}^{\left(s\right)}u_{l})\overset{s^{\prime \prime }}{\underset{j^{\prime \prime }=1}{\prod }}(1+F_{{\mathrm{jj}}^{\prime \prime }}^{\left(s\right)}y_{j^{\prime \prime }})}$$

where σ=σ(j) is the index for the reaction through which $y_{j}$ is activated. The term $K_{{\mathrm{jj}}^{\prime }}^{\left(s\right)}$ represents the strength of binding of $y_{j^{\prime }}$ with $y_{j}$, whereas $F_{\mathrm{jk}}^{\left(s\right)},\;F_{\mathrm{jl}}^{\left(s\right)}$ and $F_{{\mathrm{jj}}^{\prime \prime }}^{\left(s\right)}$ represent binding constants corresponding to $z_{k},\;u_{l}$ and $y_{j^{\prime \prime }}$ for the interaction activating $y_{j}$. Again, if some metabolites $z_{k}$ and external inputs $u_{l}$ accelerate the activation of $y_{j}$, the initial interaction rate is increased by a multiplication factor. Thus, Eq. (19) becomes

(20) $$r_{\sigma }^{\left(s\right)}=\overset{m^{\prime }}{\underset{k=1}{\prod }}(1+F_{\mathrm{jk}}^{\left(s\right)}z_{k})\overset{c^{\prime }}{\underset{l=1}{\prod }}(1+F_{\mathrm{jl}}^{\left(s\right)}u_{l})\overset{s^{\prime }}{\underset{j^{\prime }=1}{\prod }}K_{{\mathrm{jj}}^{\prime }}^{\left(s\right)}y_{j^{\prime }\neq j}$$

Thus, based on Eq. (13), the differential equation representing the dynamics of the signaling network can be rewritten as

(21) $$\overset{˙}{y}=\frac{1}{\omega_{1}}(N^{\left(s\right)}r^{\left(s\right)})$$

Similar to the signal transduction pathway, a specific stoichiometry is also associated with a metabolic pathway. Thus, we can consider a stoichiometric matrix $N^{\left(m\right)}$ of order m×n for the metabolic network under consideration, where *m* is number of metabolites participating in *n* metabolic reactions in the network with metabolic flux vector $r^{\left(m\right)}$ (in timescale $\tau_{2}$) of dimension *n*. The ${\left(k,\rho \right)}^{\mathrm{th}}$ element of $N^{\left(m\right)}$ becomes +1 if $k^{\mathrm{th}}$ metabolite is produced through $\rho^{\mathrm{th}}$ reaction. On the other hand, if $k^{\mathrm{th}}$ metabolite is consumed in $\rho^{\mathrm{th}}$ reaction, the ${\left(k,\rho \right)}^{\mathrm{th}}$ element of $N^{\left(m\right)}$ becomes -1.

Let us consider a metabolite $z_{k}$ be produced by $m^{\prime }$ ($1⩽m^{\prime }<m$) substrates $z_{k^{\prime }}$ ($k^{'}\;\neq \;k,\;k^{'}=1,\ldots ,m^{'}$) in a metabolic reaction with initial rate $r_{\rho }^{\left(m\right)},\;\rho^{\mathrm{th}}$ (ρ=1,…,n) element of $r^{\left(m\right)}$. This reaction is catalyzed by an enzyme/protein $E_{\rho }$ (representing the expression level) produced from an expressed gene. Furthermore, let us assume that $m^{\prime \prime }$ ($1⩽m^{\prime \prime }<m$) metabolites $z_{k^{\prime \prime }}$ ($k^{\prime \prime }\;\neq \;k^{\prime },k^{\prime \prime }=1,\ldots ,m^{\prime \prime }$) and $c^{\prime }$ external inputs $u_{l}$ accelerate (noncompetitively or allosterically) the production of $z_{k}$. Thus, the initial reaction rate $r_{\rho }^{\left(m\right)}$ is increased by a multiplication factor. Then, we can write using modified Michaelis Menten kinetic equation [13], [78], [14] as

(22) $$r_{\rho }^{\left(m\right)}=\frac{\overset{m^{\prime \prime }}{\underset{k^{\prime \prime }=1}{\prod }}(1+F_{{\mathrm{kk}}^{\prime \prime }}^{\left(m\right)}z_{k^{\prime \prime }})\overset{c^{\prime }}{\underset{l=1}{\prod }}(1+F_{\mathrm{kl}}^{\left(m\right)}u_{l})K_{\rho }^{\left(m\right)}E_{\rho }\overset{m^{\prime }}{\underset{k^{\prime }=1}{\prod }}z_{k^{\prime }}}{K_{\rho }^{M^{\left(m\right)}}+(\overset{m^{\prime }}{\underset{k^{\prime }=1}{\prod }}z_{k^{\prime }})}$$

where ρ=ρ(k) (could be one-to-many mapping) is the index for the reaction through which $z_{k}$ is produced. The terms $K_{\rho }^{\left(m\right)}$ and $K_{\rho }^{M^{\left(m\right)}}$ represent the kinetic rate constant and Michaelis Menten constant respectively. Besides, the terms $F_{{\mathrm{kk}}^{\prime \prime }}^{\left(m\right)}$ and $F_{\mathrm{kl}}^{\left(m\right)}$, for a $k^{\mathrm{th}}$ metabolite, represent feedback constants corresponding to $z_{k^{\prime \prime }}$ and $u_{l}$. Again, if $m^{\prime \prime }$ metabolites $z_{k^{\prime \prime }}$ and $c^{\prime }$ external inputs $u_{l}$ slow down (noncompetitively or allosterically) the production of $z_{k}$, the initial reaction rate $r_{\rho }^{\left(m\right)}$ is decreased by a fraction. Thus, we can modify Eq. (22) as

(23) $$r_{\rho }^{\left(m\right)}=\frac{K_{\rho }^{\left(m\right)}E_{\rho }\overset{m^{\prime }}{\underset{k^{\prime }=1}{\prod }}z_{k^{\prime }}}{\left(K_{\rho }^{M^{\left(m\right)}}+(\overset{m^{\prime }}{\underset{k^{\prime }=1}{\prod }}z_{k^{\prime }}))\overset{m^{\prime \prime }}{\underset{k^{\prime \prime }=1}{\prod }}(1+F_{{\mathrm{kk}}^{\prime \prime }}^{\left(m\right)}z_{k^{\prime \prime }})\overset{c^{\prime }}{\underset{l=1}{\prod }}(1+F_{\mathrm{kl}}^{\left(m\right)}u_{l}\right)}$$

Now, based on Eq. 14, the differential equation representing the dynamics of the metabolic network can be rewritten as

(24) $$\overset{˙}{z}=\frac{1}{\omega_{1}\omega_{2}}(N^{\left(m\right)}r^{\left(m\right)})$$

### 2.3 Design of a MIMO plant

The initial concentrations/expressions of metabolites, signaling molecules and genes have been considered in (0, 1) randomly (Supplementary Table S9). Moreover, the kinetic parameter values have also been initialized randomly within the same interval (Supplementary Tables S12–S15). Based on these initial values, we have simulated the proposed three timescale state space model of an integrated biochemical pathway by solving ODEs in Eqs. 17 (or 18), 21 and 24 with proper timescale selection, and solved for u based on x,y and z values under equilibrium condition. If the model fails to mimic the known behavioral pattern of the integrated biochemical pathway under consideration, we have altered slightly the initial values of kinetic parameters in (0, 1) with the help of previous knowledgebase by trial and error until satisfactory known behavior is captured. Once the proposed model has mimicked the known nonlinear dynamics of the integrated biochemical pathway (as described in SubSection 3.1), we have considered it as normal MIMO plant with the selected values of initial concentrations/expressions and kinetic parameters. This MIMO plant mimics the normal behavior of the concerned integrated pathway.

**Fig. 3:**
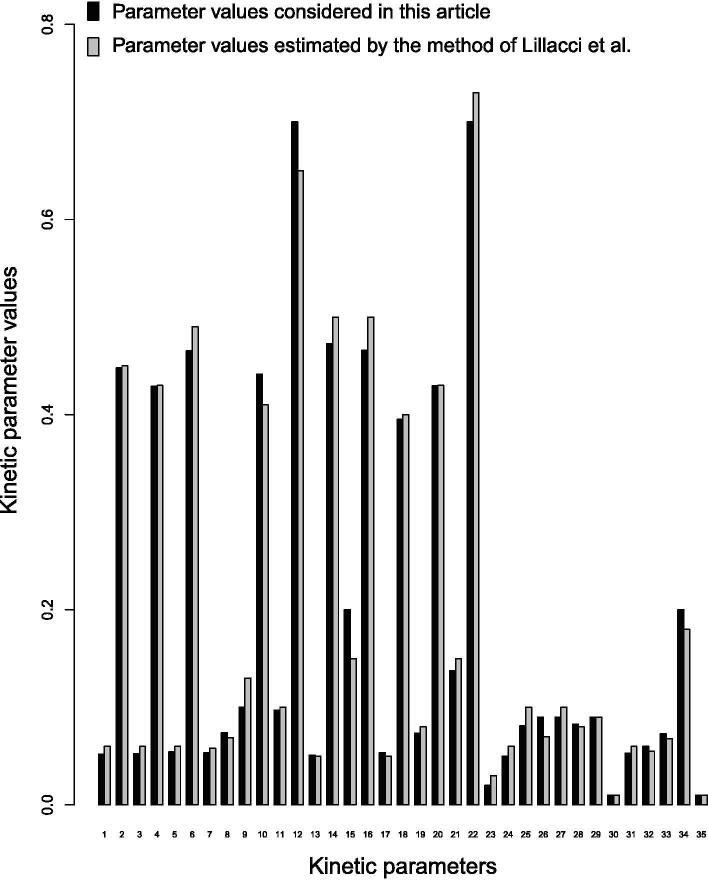
Comparison of kinetic parameter values considered in the present article with those estimated by the method of Lillacci et al. [70]. Here first 22 kinetic parameters represent glycolytic Michaelis Menten constants, whereas last 13 kinetic parameters correspond to glycolytic kinetic rate constants.

**Fig. 4:**
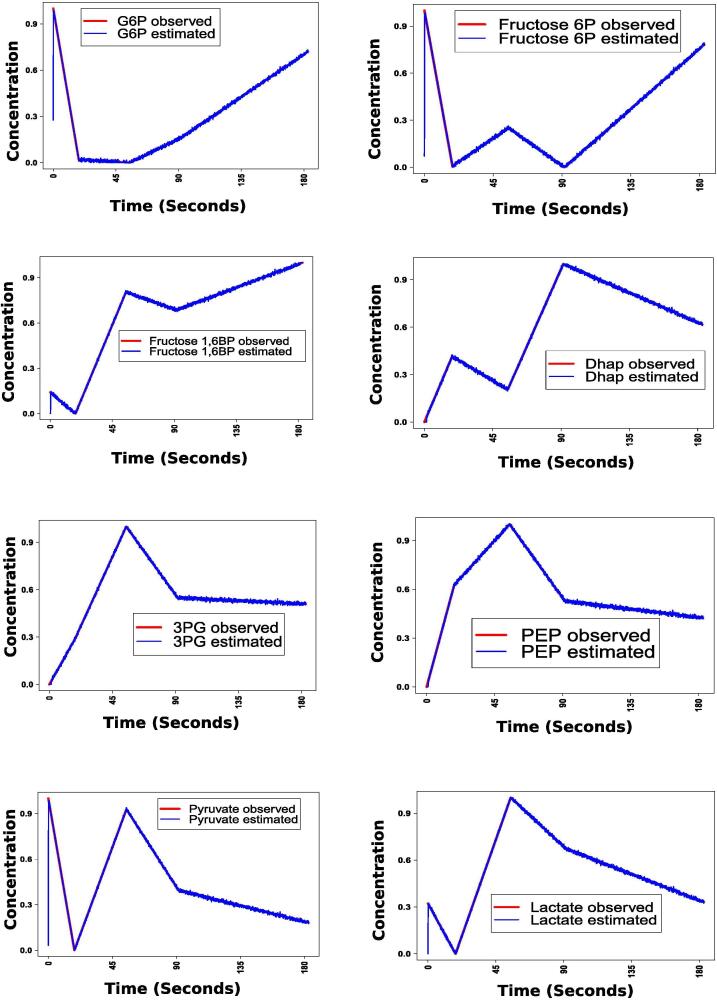
Estimated states have been overlapped with observed true values of the corresponding states during the estimation of 35 kinetic parameters using hybrid extended Kalman filtering based method [70].

In this context, we have checked whether trial and error based parameter estimation resembles other previous method. For this purpose, we have compared the values of 35 kinetic parameters considered here with those estimated by a method based on hybrid extended Kalman filtering [70]. Fig. 3 depicts that the parameter values in (0, 1) considered in this article are quite similar with those estimated by hybrid extended Kalman filtering based method [70]. To estimate these 35 kinetic parameter values in (0, 1) using hybrid extended Kalman filtering, time dependent true values in (0, 1) of some observed states need to be provided. Consequently, we have used capillary electrophoresis mass spectrometry (CE-MS) measured concentration values (normalized in (0, 1)) of eight metabolites in hypoxia-induced metabolic alterations in human erythrocytes [71] as observed states. Here we have externally applied appropriate signal in (0, 1) for corresponding enzyme activities related to hypoxia in human erythrocytes (as described in SubSection 3.1). During the estimation of these 35 parameter values using hybrid extended Kalman filtering, we have noticed that estimated states have been overlapped with observed states as depicted in Fig. 4. However, we could not verify the other parameter values using this way due to lack of required observed states.

Now, we perturb the normal MIMO plant by knocking out genes (in this study, genes producing the enzymes pyruvate dehydrogenase and pyruvate carboxylase in carbon metabolism) to capture the nonlinear dynamics associated with a particular biological phenomenon (in this study, “Warburg effect” [24], [25]). As a result, the normal plant becomes the desired perturbed MIMO plant under consideration.

### 2.4 Generation of training dataset

So far we have developed the desired perturbed MIMO plant with consideration of appropriate three timescales. It does not reflect the mutated regulations of a mammalian cancer cell. However, it contains the perturbations associated with “Warburg effect”. In order to capture the altered dynamics of a cancer cell, we need a model predictive controller to be applied on an approximate model corresponding to the MIMO plant under consideration [79]. We are going to develop such an approximate model using support vector regression. Thus, the past input and output data simulated from the MIMO plant under investigation are collected to serve as a training dataset for the SVR-based approximation. Here we have found that consideration of τ (=19) number of past inputs and corresponding outputs along with current input (at time θ), from the simulated data as an input sample of SVR, and current output (at time θ) as corresponding supervised signal of the regression model, has generated maximum test accuracy (99.4%) with mean squared error (MSE) threshold of 0.05. Now, we are going to discuss how such training data have been generated.

Let us consider the MIMO plant under consideration takes an instance $\langle x,y,z,u\rangle_{\left(\theta -\tau \right)}^{\left(\mathrm{in}\right)}$ as input at time (θ-τ) and generate corresponding output instance $\langle x,y,z\rangle_{\left(\theta -\tau \right)}^{\left(\mathrm{out}\right)}$. Using the output instance $\langle x,y,z\rangle_{\left(\theta -\tau \right)}^{\left(\mathrm{out}\right)}$ at time (θ-τ), a new instance of u has been determined, followed by formation of the new input instance $\langle x,y,z,u\rangle_{\left(\theta -(\tau -1)\right)}^{\left(\mathrm{in}\right)}$ for the MIMO plant at time (θ-(τ-1)). Likewise, we have generated 19 (*i.e.*, τ=19) past inputs and outputs to form a current input instance $\langle x,y,z,u\rangle_{\theta }^{\left(\mathrm{in}\right)}$ and corresponding output instance $\langle x,y,z\rangle_{\theta }^{\left(\mathrm{out}\right)}$ at time θ. In general, the total number of elements in $\langle x,y,z,u\rangle^{\left(\mathrm{in}\right)}$ is (p+s+m+c), while that in $\langle x,y,z\rangle^{\left(\mathrm{out}\right)}$ is (p+s+m). Here we have considered p=37,s=29,m=41 and c=27 for carbon metabolic pathway obtained from KEGG.

The output of the MIMO plant depends not only on the current input but also on its past inputs and outputs. We have, therefore, considered an input vector v to be applied to SVR model as.

$\left(\langle x,y,z,u\rangle_{\left(\theta -19\right)}^{\left(\mathrm{in}\right)}\;\;\langle x,y,z\rangle_{\left(\theta -19\right)}^{\left(\mathrm{out}\right)}\;\;\langle x,y,z,u\rangle_{\left(\theta -18\right)}^{\left(\mathrm{in}\right)}\;\;\langle x,y,z\rangle_{\left(\theta -18\right)}^{\left(\mathrm{out}\right)}\;\ldots \;\;\langle x,y,z,u\rangle_{\theta }^{\left(\mathrm{in}\right)}\right)$.

Thus, the number of inputs to SVR model becomes (19×(2×(p+s+m)+c)+(p+s+m+c))=4713, which is the dimension (*d*) of v. Now, the current output instance $\langle x,y,z\rangle_{\theta }^{\left(\mathrm{out}\right)}$ has been attached to v, as its supervised signal, to form one sample to be included in a dataset D. If there are ϕ such samples in D, let us call it as $D_{\phi }$. Here we have considered ϕ=60000. We have selected 65% data from $D_{\phi }$ as training data and remaining 35% data as test data to design the SVR model to be discussed in the following section. During training of the SVR model, we aim to utilize a minimum number of a training pattern in order to improve generalization performance. To assess the best possible ratio between train and test data, we have observed the performance with different ratios. The accuracy of such cases is given in Table 2. Here, it can be concluded that the train to test ratio as 65:35 may be the best trade-off between the number of patterns used for training and the test accuracy. Thus we have considered train to test data ratio accordingly.

**Table 2:** Test accuracy of SVR model with different train to test data ratio.

Serial Number	Train:test	Test accuracy
1	90:10	99.90%
2	80:20	99.50%
3	70:30	99.50%
4	65:35	99.40%
5	60:40	95.25%

### 2.5 Design of SVR model

From control theoretic point of view, it is difficult to apply online optimization on actual nonlinear MIMO plant with small sampling periods [80], [81]. Moreover, due to large number of variables involved in an optimization problem, and requirement of high sampling rate, it may not be possible to manage online optimization. However, it will be easier if we can provide a model that suitably approximates the nonlinear dynamics of actual MIMO plant. We also think that such implementation may be relevant for future study to determine drug dosages in real time. Even this kind of modelling will be effective in future when actual input–output mapping of the MIMO plant is unknown, whereas the inputs and corresponding outputs with time are known. The training dataset $D_{\phi }$ helps in developing the approximate MIMO SVR model to mimic the nonlinear dynamics of the MIMO plant/system under consideration. In this context, it should be mentioned that according to some recent studies [82], [83], [84], SVR has better performance accuracy than an artificial neural network (ANN) (particularly, multi-layer perceptron (MLP)) and genetic programming (GP). In general, the computational complexity can be evaluated depending mainly on training time. SVR depends on solving a quadratic problem, where a vector and bias need to be calculated. Even during training, only support vectors are selected regarding the kernel. Besides, SVR considers that the number of support vectors is governed by very limited number of training samples. In addition, most of the kernels compute simple dot product. Even SVR may act better than ANN as per the consistency with physical behavior [85]. Moreover, it has been claimed that the SVR technique not only achieves high consistency but also shows a greater degree of generalization to unseen test data compared to some other methods [86]. Besides, a less number of parameters involved in the training phase results in less computation time than that of an ANN [87].

Now, we are going to discuss how a MIMO SVR model can be designed using support vector regression [88], [89].

An approximate MIMO SVR model has been developed by combining multiple input single output (MISO) SVR models for each $\mu^{\mathrm{th}}$ (1⩽μ⩽(p+s+m)) element of actual output $\langle x,y,z\rangle_{\theta }^{\left(\mathrm{out}\right)}$. Let $a^{\mathrm{th}}$ input be $v_{a}$ (1⩽a⩽ϕ) of dimension *d*. For an $a^{\mathrm{th}}$ input, the MISO SVR model for $\mu^{\mathrm{th}}$ element of $\langle x,y,z\rangle_{\theta }^{\left(\mathrm{out}\right)}$ in a projected feature space *F* of dimension $d^{\prime }$ ($d^{\prime }>d$) can be written as

(25) γ^μ=wμTδ(va)+βμ

Here γ^μ represents $\mu^{\mathrm{th}}$ element of predicted output 〈x^,y^,z^〉θ(out). The term $w_{\mu }$ represents a vector perpendicular to the hyperplane to be approximated for the regression problem under consideration in the projected feature space *F* corresponding to $\mu^{\mathrm{th}}$ element of $\langle x,y,z\rangle_{\theta }^{\left(\mathrm{out}\right)}$. In other words, $w_{\mu }$ determines the orientation of the hyperplane in *F*. On the other hand, δ(.) is a mapping from *d* dimensional input space to $d^{\prime }$ dimensional feature space *F*. The bias term is represented by $\beta_{\mu }$ determining the position of the hyperplane in *F* corresponding to $\mu^{\mathrm{th}}$ element of $\langle x,y,z\rangle_{\theta }^{\left(\mathrm{out}\right)}$.

Based on Vapnik’s ε-insensitive loss function, the convex optimization problem corresponding to Eq. (25) can be formulated as [90]

(26) $$\underset{w_{\mu },\beta_{\mu },\varphi ,\varphi^{*}}{\min}P_{\mu }=\frac{1}{2}\|w_{\mu }{\|}^{2}+C\overset{\phi }{\underset{a=1}{\sum }}(\varphi_{a}+\varphi_{a}^{*})$$

subject to:

$$\begin{array}{c} \gamma_{\mu }-w_{\mu }^{T}\delta (v_{a})-\beta_{\mu }⩽\varepsilon_{\mu }+\varphi_{a},\;\;\mathrm{for}\;\;a=1,\cdots ,\phi \\ w_{\mu },\delta (v_{a})+\beta_{\mu }-\gamma_{\mu }⩽\varepsilon_{\mu }+\varphi_{a}^{*},\;\;\mathrm{for}\;\;a=1,\cdots ,\phi \\ \varphi_{a},\varphi_{a}^{*}⩾0,\;\;\mathrm{for}\;\;a=1,\cdots ,\phi \end{array}$$

Here, the upper limit of tolerable error is a very small positive real quantity $\varepsilon_{\mu }$ for $\mu^{\mathrm{th}}$ element of $\langle x,y,z\rangle_{\theta }^{\left(\mathrm{out}\right)}$, whereas $\varphi_{a}$ and $\varphi_{a}^{*}$ are slack variables. The term $\gamma_{\mu }$ represents the actual value of $\mu^{\mathrm{th}}$ element of $\langle x,y,z\rangle_{\theta }^{\left(\mathrm{out}\right)}$ generated by the MIMO plant taking $\langle x,y,z,u\rangle_{\theta a}^{\left(\mathrm{in}\right)}$ (extracted from $v_{a}$) as an $a^{\mathrm{th}}$ input. The trade-off between flatness of γ^μ and tolerance level of deviations ($>\varepsilon_{\mu }$) is represented by the constant C (>0).

Based on the notion of Lagrange multipliers and Karush Kuhn Tucker (KKT) condition [91], the dual corresponding to Eq. (26), in the form of a quadratic programming problem with incorporation of kernel function, can be written as

(27) $$\underset{\zeta ,\zeta^{*}}{\min}Q_{\mu }=\frac{1}{2}\overset{\phi }{\underset{a=1}{\sum }}\overset{\phi }{\underset{b=1}{\sum }}(\zeta_{a}-\zeta_{a}^{*})(\zeta_{b}-\zeta_{b}^{*})\kappa (v_{a},v_{b})+\varepsilon_{\mu }\overset{\phi }{\underset{a=1}{\sum }}(\zeta_{a}+\zeta_{a}^{*})-\overset{\phi }{\underset{a=1}{\sum }}\gamma_{\mu }(\zeta_{a}-\zeta_{a}^{*})$$

subject to:

$$\begin{array}{c} 0⩽\zeta_{a},\;\zeta_{a}^{*}⩽C,\\ \overset{\phi }{\underset{a=1}{\sum }}(\zeta_{a}-\zeta_{a}^{*})=0,\;\;\mathrm{for}\;\;a=1,\cdots ,\phi \end{array}$$

Here the term $\kappa (v_{a},v_{b})$ denotes the kernel function. Besides, $\zeta_{a},\;\zeta_{a}^{*},\;\zeta_{b}$ and $\zeta_{b}^{*}$ stand for Lagrange multipliers. The solution of Eq. (27) provides the optimum values of $\zeta_{a},\;\zeta_{a}^{*},\;\zeta_{b}$ and $\zeta_{b}^{*}$. Moreover, $w_{\mu }-\sum_{b=1}^{\phi }(\zeta_{b}-\zeta_{b}^{*})\delta (v_{b})=0$ must hold for optimality. Thus, for an $a^{\mathrm{th}}$ input, the support vector kernel expansion of Eq. (25) can be obtained from

(28) γ^μ=∑bαbκ(va,vb)+βμ

The summation has been carried out over all the support vectors $v_{b}$. Here $\alpha_{b}=(\zeta_{b}-\zeta_{b}^{*})$. An input vector $v_{b}$ corresponding to a non-zero $\alpha_{b}$ is called a support vector. The value of $\beta_{\mu }$ is determined when |γμ-γ^μ|=εμ and $0⩽\zeta_{b}-\zeta_{b}^{*}⩽C$ hold for each support vector $v_{b}$. Thus, Eq. (28) provides the required $\mu^{\mathrm{th}}$ element of predicted output 〈x^,y^,z^〉θ(out), taking $v_{a}$ as input, corresponding to $\mu^{\mathrm{th}}$ element of actual output $\langle x,y,z\rangle_{\theta }^{\left(\mathrm{out}\right)}$.

Here, we have experimentally chosen radial basis kernel function with kernel coefficient as 10, regularization parameter as 100 and $\varepsilon_{\mu }$=0.0025 to achieve maximum test accuracy.

### 2.6 Design of a controller using a genetic algorithm (GA)

In this study, we have applied a GA [89] on the SVR model as a model predictive controller to control some outputs of the MIMO plant at specific values. There are many reasons behind choosing GA as an optimization technique. Firstly, a genetic algorithm (GA) [92], [93] is a stochastic process. Secondly, it is a vigorous search technique, which does not require any information about the structure of the function to be optimized. Such a situation is very common to address a biological system, particularly during mutation. Thirdly, GA is very efficient in handling highly complicated non-linear problems, such as an integrated biochemical pathway system. Besides, due to its inherent parallelism, GA can easily be implemented in a distributed environment. Such a characteristic is very much helpful in handling a large number of parameters/variables in a biological system. Fourthly, GA may be able to avoid being trapped in a locally optimal solution, unlike traditional methods. Besides, GA can efficiently be applied to different large scale real-world problems with the requirement of multi-objective optimization, such as the particular problem addressed in this article.

**Fig. 5:**
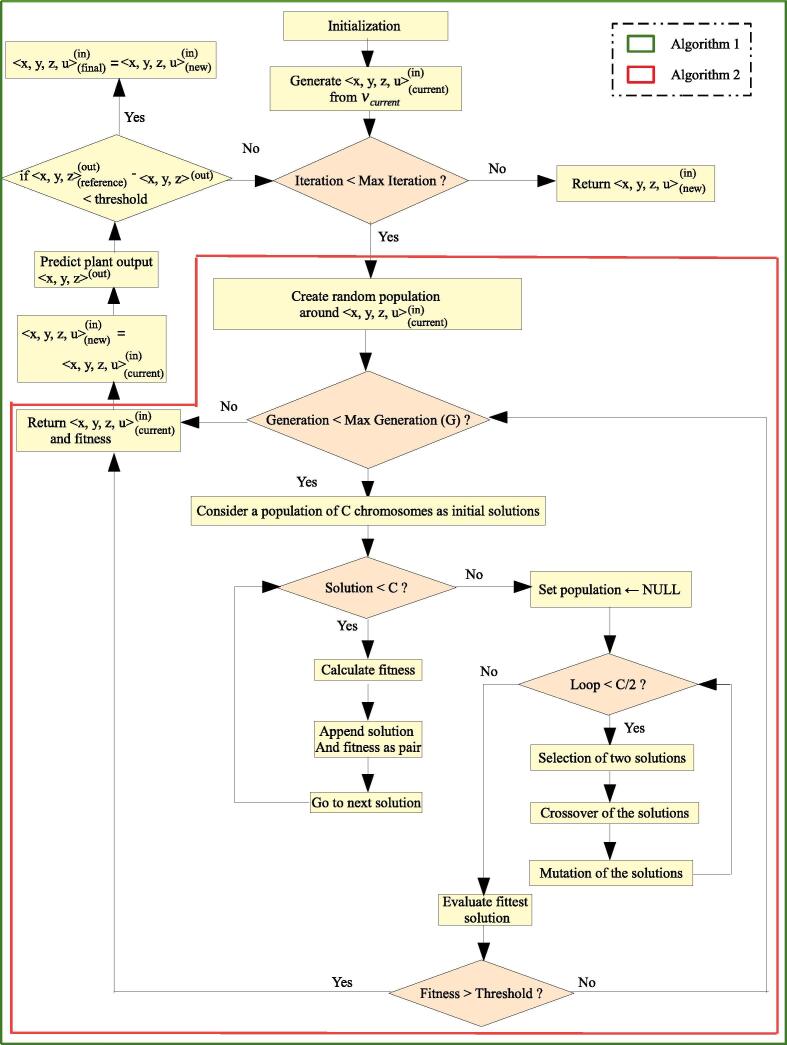
Illustrating the flowchart showing the functionalities of two Algorithms S1 and S2 for designing GA-based controller.

Such a GA based control mechanism has been depicted in Fig. 2 (Step 6). Here the error between SVR generated predicted output and (user-specified) reference output drives the GA based controller to produce a controlled input based on some cost functions and constraints. The integrated biochemical network (*i.e.*, the MIMO plant) accepts the controlled input to compute actual output. The GA controller has been developed using two algorithms (Supplementary Algorithms S1 and S2). Supplementary Algorithm S1 deals with the working principles of a GA controller. On the other hand, the Supplementary Algorithm S2 drives some outputs of the MIMO plant to target values using Supplementary Algorithm S1 iteratively. Now, we are going to discuss Supplementary Algorithms S1 and S2 in brief. Here, Fig. 5 depicts the flowchart indicating the role and sequence of two Algorithms S1 and S2 for designing GA-based controller.

• Supplementary Algorithm S1 shows how proposed GA controller works. Here the GA controller accepts $\langle x,y,z,u\rangle_{\mathrm{current}}^{\left(\mathrm{in}\right)}$ and $v_{\mathrm{current}}$ as input. At first, it generates *C* chromosomes (set of possible solutions) randomly, forming a population. However, it creates chromosomes around $\langle x,y,z,u\rangle_{\mathrm{current}}^{\left(\mathrm{in}\right)}$ after getting an optimal solution. For each generation, the fitness value of each chromosome has been computed. Thereafter, the chromosomes are modified by selection, crossover and mutation. In this context, a set of chromosomes is selected according to fitness values greater than a weight value. Subsequently, the crossover is performed by swapping a part of chromosome with corresponding part of another chromosome according to a crossover index. In this context, it should be mentioned that as the size of the output vector is 107 (=(p+s+m) as mentioned earlier), we have considered crossover index as 53. This index is nearly half of the size of the output vector. It is followed by mutation to modify chromosomes with mutation probability 0.7. Here, we have used uniform crossover [94], [95], *i.e.*, multi-point crossover, to reduce the mutation bias. These steps are carried out for *G* generations until the fittest chromosome, whose fitness value is greater than a predefined threshold value, has been evolved as an optimal solution $\langle x,y,z,u\rangle_{\mathrm{new}}^{\left(\mathrm{in}\right)}$.
• Supplementary Algorithm S2 depicts how some outputs of the MIMO plant are controlled to get desired responses with repetitive use of GA controller (Supplementary Algorithm S1). The execution of Supplementary Algorithm S2 needs the trained SVR model, actual MIMO plant, initial SVR input $v_{\mathrm{current}}$ and reference output $\langle x,y,z\rangle_{\mathrm{reference}}^{\left(\mathrm{out}\right)}$. As per aforesaid discussion, the GA controller receives $\langle x,y,z,u\rangle_{\mathrm{current}}^{\left(\mathrm{in}\right)}$ (extracted from $v_{\mathrm{current}}$) and $v_{\mathrm{current}}$ itself to compute an optimal solution $\langle x,y,z,u\rangle_{\mathrm{new}}^{\left(\mathrm{in}\right)}$. However, during iteration > 1 of Supplementary Algorithm S2, if the fitness value of an optimal solution (chromosome) becomes less than that of previous iteration, the GA controller tries again to find a better optimal solution using the same $\langle x,y,z,u\rangle_{\mathrm{current}}^{\left(\mathrm{in}\right)}$. After obtaining an optimal solution $\langle x,y,z,u\rangle_{\mathrm{new}}^{\left(\mathrm{in}\right)}$, the initial SVR input has been updated to $v_{\mathrm{new}}$. Subsequently, we have computed predicted output 〈x^,y^,z^〉(out) and actual output $\langle x,y,z\rangle^{\left(\mathrm{out}\right)}$ from the SVR model and MIMO plant respectively, using $v_{\mathrm{new}}$ and $\langle x,y,z,u\rangle_{\mathrm{new}}^{\left(\mathrm{in}\right)}$. Here we have considered the reference output $\langle x,y,z\rangle_{\mathrm{reference}}^{\left(\mathrm{out}\right)}$ containing target values for some specific elements (for example, 0.95 for ATP and 0.7 for ribose 5P), whereas others remain at the same values as computed in $\langle x,y,z\rangle^{\left(\mathrm{out}\right)}$. We have finally obtained the final solution $\langle x,y,z,u\rangle_{\mathrm{final}}^{\left(\mathrm{in}\right)}$ when the L_1_-norm of the difference between $\langle x,y,z\rangle_{\mathrm{reference}}^{\left(\mathrm{out}\right)}$ and $\langle x,y,z\rangle^{\left(\mathrm{out}\right)}$ becomes less than a predefined threshold value after several iterations.

Finally, it should be mentioned that the average value of G is 10 for convergence of Algorithm S1, whereas 30 iterations on average are needed for Algorithm S2 to converge.

## 3 Results and discussion

This section has been divided into three subsections. Firstly, we are going to discuss about MIMO plant validation through analysis of both normal and perturbed behavior of the integrated biochemical pathways under consideration. Secondly, the altered behavior of the integrated biochemical pathways in mammalian cancer cells compared to normal ones will be discussed. Finally, the third subsection deals with the description of the effects of six possible drug targets in mammalian cancer cells.

### 3.1 MIMO plant validation and comparison with other mathematical prediction

Here, we have monitored the concentrations of key molecules during simulation of the proposed three timescale state space model for normal as well as perturbed integrated biochemical pathways related to carbon metabolism. In normal scenario (Fig. S2 in Supplementary material), both glucose consumption and production increase. Glucose is broken down into glucose-6P (G6P). Subsequently, the energy in the form of ATP is consumed. Thus, increasing glucose consumption signifies enhancement of G6P production. The production of ribose 5P and NADPH increases by utilizing higher amount of G6P through PPP. On the other hand, slowing down glyceraldehyde-3P production indicates higher flux through later phase of glycolysis by consuming higher amount of glyceraldehyde-3P. Higher concentration of phosphoenolpyruvate (PEP) supports our claim. Similarly, slowing down the production of pyruvate and acetyl CoA indicates higher flux through TCA cycle and fatty acid synthesis. Enhanced concentrations of oxaloacetate (OAA), citrate and FFA are in conformity with our observation. ATP production increases with higher flux through TCA cycle and later phase of glycolysis. However, after a while ATP decreases due to its higher consumption at early phase of glycolysis. Previous investigations [11], [96], [97] validate the aforesaid behavior of the proposed model under normal scenario.

**Fig. 6:**
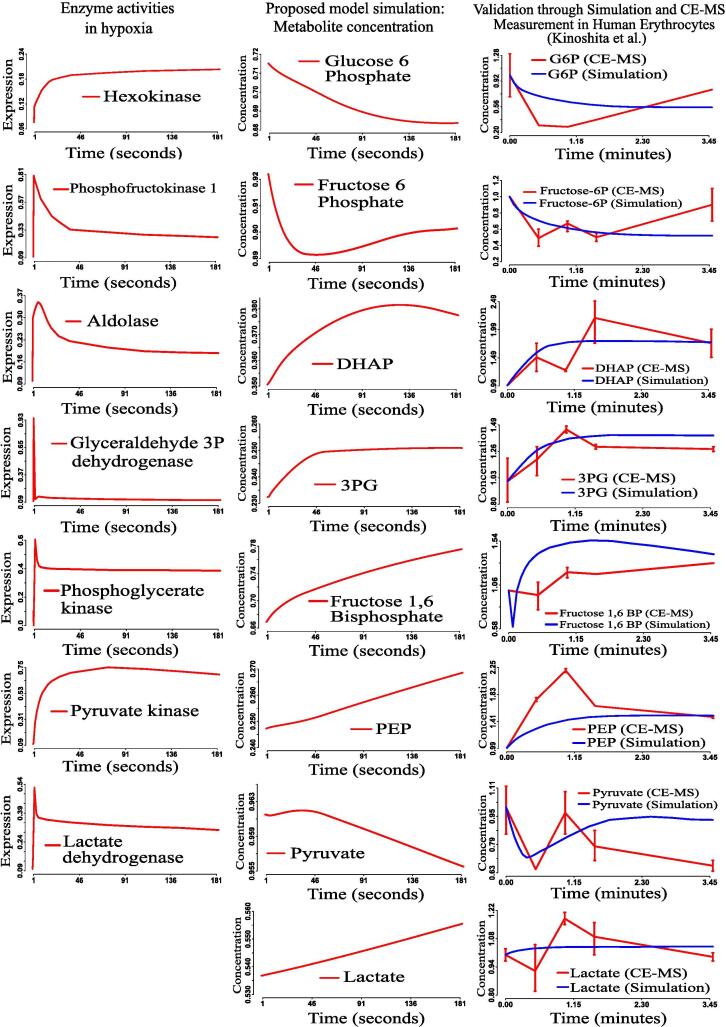
Validation of the proposed model during hypoxia through CE-MS measurement and simulation in human erythrocytes (Kinoshita et al. [71]).

We have further compared the model with a previous simulation result [71] for an environment in cancer cells, particularly, hypoxia condition [98]. Here, we have perturbed the present model to incorporate the mutation exhibiting hypoxia condition. Moreover, the simulation results have been compared with CE-MS measurements during hypoxia in human erythrocytes [71]. As hypoxia in human erythrocytes enhances the expression of certain glycolytic enzymes including hexokinase, aldolase and pyruvate kinase [71], we have applied these enzyme expression values in (0, 1) to the model following their activities as described in the previous investigation [71]. Fig. 6 depicts that our simulation results almost follow not only the CE-MS measurements but also the simulation results obtained by the study [71] in response to the enzyme activities due to hypoxia in human erythrocytes. We have calculated mean squared error (MSE) between our simulated concentrations of eight metabolites (G6P, fructose-6P, fructose 1, 6 bisphosphate, Dhap, 3PG, PEP, pyruvate, and lactate) with time, and the corresponding CE-MS measurements of the same due to aforementioned enzyme activities during hypoxia in human erythrocytes. The MSE values, corresponding to these eight metabolites, are (0.07337, 0.06031, 0.074389, 0.12327, 0.13879, 0.14829, 0.34182, and 0.05727). In addition, we have calculated the MSE between the simulation results of Kinoshita et al. [71] and the same CE-MS measurements as before. Here, we have found MSE values as (0.05610, 0.027992, 0.10074, 0.12518, 0.12673, 0.15056, 0.15697, and 0.17443). It indicates that MSE values, corresponding to our simulation results, are slightly higher for G6P, Fructose-6P, 3PG, and Pyruvate than the same corresponding to Kinoshita et al. On the other hand, our simulation results are closer to CE-MS measurements than that of Kinoshita et al. for Fructose 1, 6 bisphosphate, Dhap, PEP, and Lactate in terms of the MSE values.

In comparison with some other mathematical models in analyzing energy management in mammalian cancer cells, we have found that a constraint-based flux balance model [66] of CCM pathways in cancer cells has found a set of enzymes, such as lactate dehydrogenase, playing important roles in cancer growth. Similar results have been found using our model. We have elaborately discussed the results in the following sections. However, such a flux balance analysis based modeling does not depict the transient behavior of the molecules involved in the cancer pathway. Another differential equation based model [67] has explored a quantitative relationship between the hypoxia intensity and the intracellular lactate levels in cancer cells. Besides, it has predicted some important regulators of the glycolysis pathway only. Similar type of model [68] has been developed for targeting energy metabolism in pancreatic cancer. Another model [69] based on optimization technique has shown the lactate-driven coupling for fulfilling energy requirements in cancer cells. However, unlike the proposed model, they do not consider three kinds of pathways with different response time. Moreover, they do not explore the effects of activating/deactivating the probable drug targets to control energy metabolism in cancer cells.

**Fig. 7:**
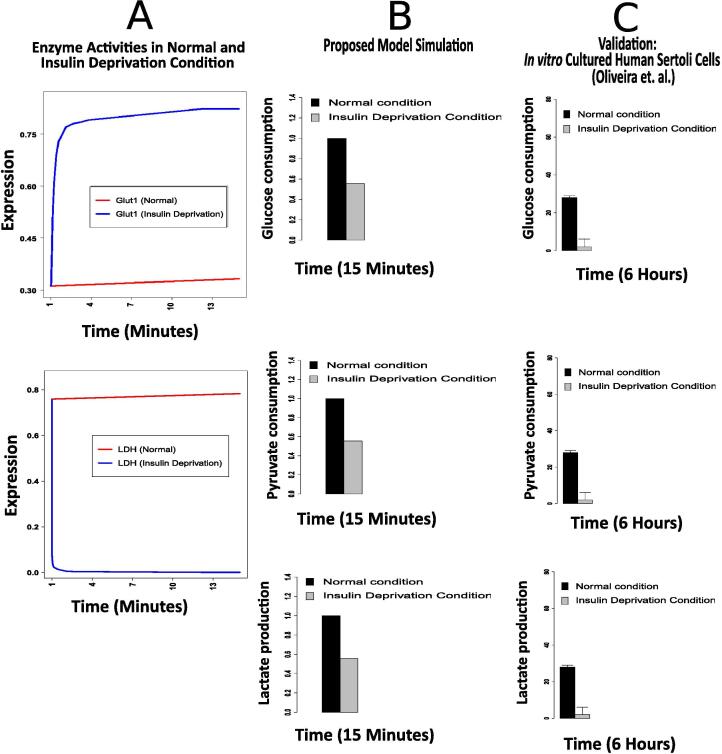
Validation of the proposed model during insulin deprivation through *in vitro* observations from cultured human sertoli cells (Oliveira et al. [72]).

For more validation of the proposed model, we have compared our simulation results with *in vitro* observations from cultured human sertoli cells during insulin deprivation [72]. Such a condition in human sertoli cells reduces the expression level of lactate dehydrogenase (LDH). Besides, the expression level of glut1 has increased [72]. Incorporation of these two enzyme expressions in (0, 1) into our model, we can notice similar behavior of initial glucose consumption, pyruvate consumption and lactate production with the corresponding behavior in cultured human sertoli cells during insulin deprivation (Fig. 7).

In the context of present investigation, we have finally simulated the situation of knocking out certain enzymes, *viz.*, pyruvate dehydrogenase, pyruvate carboxylase, acyl-CoA synthetase, fatty acid synthase, phosphoenolpyruvate carboxykinase 1 and succinyl CoA synthetase, to slow down oxidative phosphorylation through mitochondria leading to “Warburg effect” [24], [25]. In order to knock out these enzymes, we have considered the kinetic rate constants as zero for the reactions catalyzed by the above enzymes. Considering the aforesaid perturbed condition, we have monitored the behavioral alteration of different molecules compared to that in normal situation as depicted in Figures S3 and S4 in Supplementary material. Enhancement of pyruvate and lactate production as well as slowing down the production of FFA, citrate and succinate compared to that under normal condition indicates low oxidative phosphorylation. Subsequently, consumption of glucose increases to compensate less ATP production, which leads to reduction of glucose production compared to that under normal condition. In spite of this situation, ATP production decreases significantly. As most of the glucose is utilized through glycolytic flux, NADPH production decreases compared to that in normal mammalian cells. However, production of fructose 6P, acetyl CoA, ribose 5P, fructose 2,6 bisphosphate, PEP and protein kinase B (AKT) does not show any significant difference compared to that under normal condition. Interestingly, expression level of p53 increases to inhibit glucose transporter 1 (glut1) [99], which is responsible for transportation of glucose across the plasma membranes of mammalian cells [100]. Thus, higher expression of p53 leads to suppressing the production of glucose in glycolysis pathway. As a result, glucose concentration decreases rapidly (Fig. S3 in Supplementary material) leading to cell apoptosis [101].

Nevertheless, mammalian cancer cells somehow manage both energy, in the form of ATP, and cell proliferation to survive [102]. In this study, we aim at investigating the probable mutated regulation that drives mammalian cancer cells to survive despite “Warburg effect”. In this context, we have considered the aforesaid perturbed state space model of integrated biochemical pathways related to carbon metabolism as a MIMO plant of our interest. Thereafter, we have applied the GA controller to achieve high concentrations of ATP (0.95) and ribose 5P (0.70). Here, high ATP will supply constant energy in mammalian cancer cells to survive, while ribose 5P plays an important role in nucleotide synthesis promoting cell growth and proliferation [27]. Thus, the proposed MIMO plant along with GA controller mimics the altered nonlinear dynamics of mammalian cancer cells. We are now going to describe the probable mutated regulations of cancer cells in the following subsection.

### 3.2 Analysis of regulations in mammalian cancer cells

**Fig. 8:**
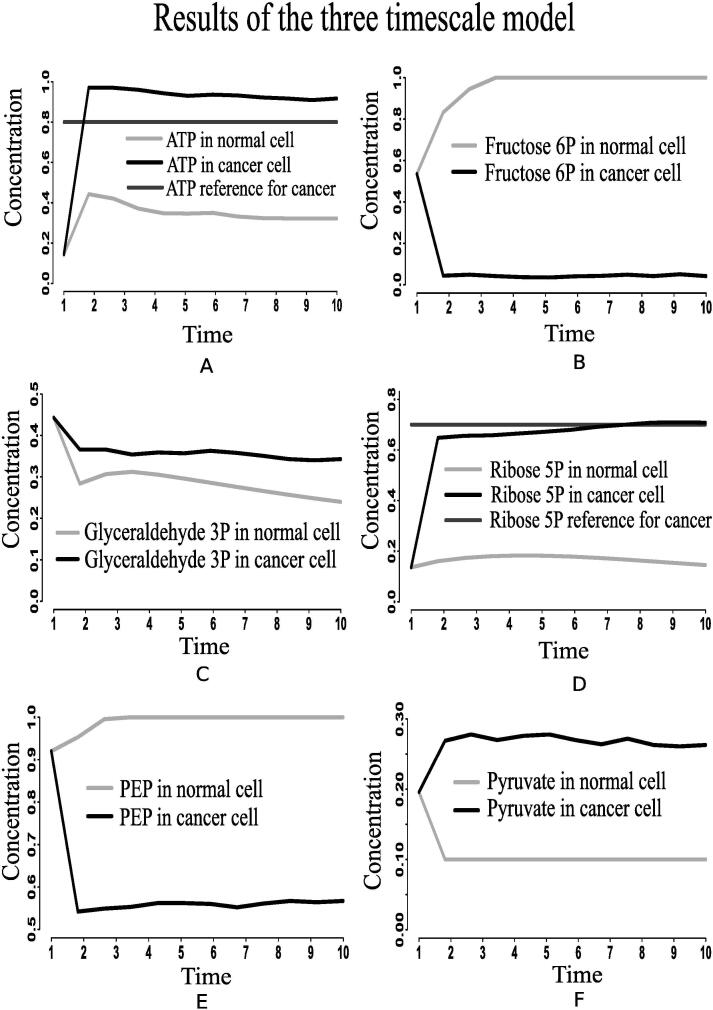
Alterations of (A) ATP, (B) fructose 6P, (C) glyceraldehyde-3P (GA3P), (D) ribose 5P, (E) PEP, and (F) Pyruvate in the integrated biochemical pathway related to carbon metabolism in mammalian cancer cells compared to that in normal ones.

**Fig. 9:**
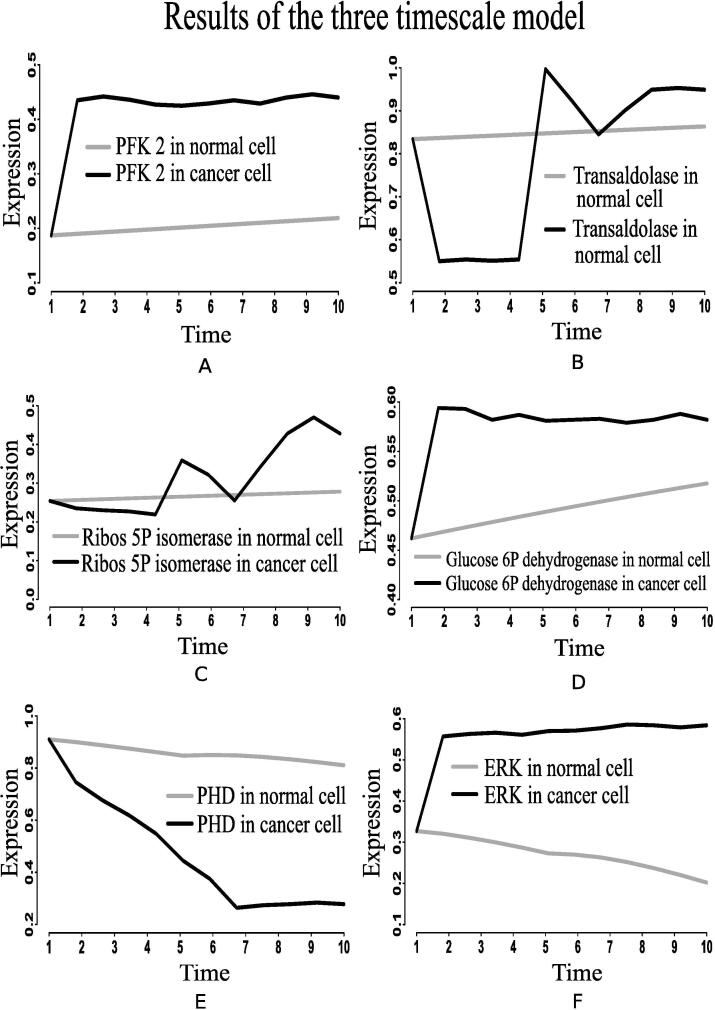
Alterations of (A) PFK2, (B) transaldolase, (C) ribose 5P isomerase, (D) glucose-6-phosphate dehydrogenase, (E) PHD, and (F) ERK in the integrated biochemical pathway related to carbon metabolism in mammalian cancer cells compared to that in normal ones.

**Fig. 10:**
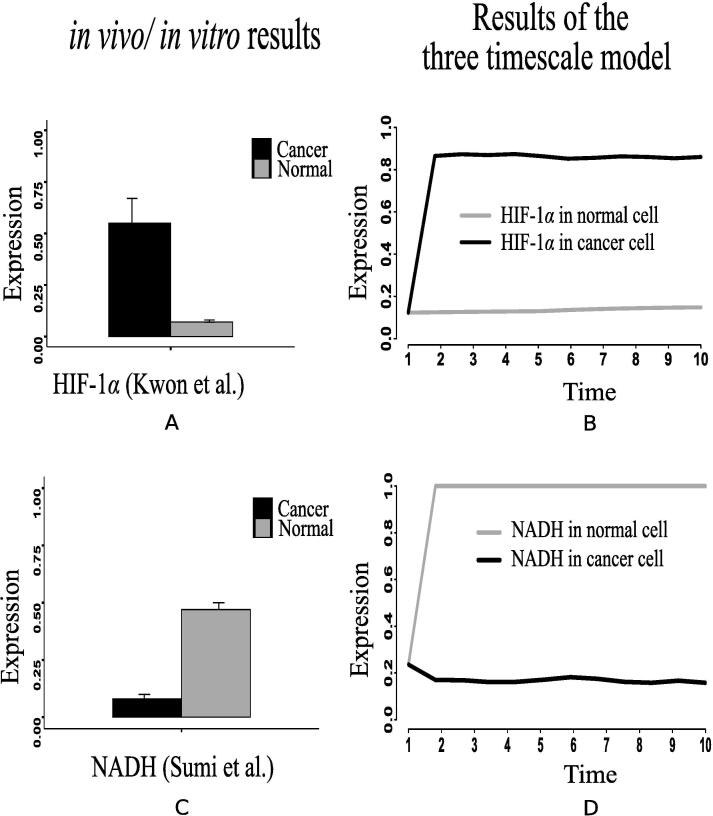
It depicts: (A) Higher expression level of HIF-1α measured in human hepatocellular carcinoma (HCC) specimens compared to the non-cancerous tissue specimens [54], (B) Similar behavior of HIF-1α expression shown by our simulation results, (C) Lower concentration of NADH in cancer cells compared to normar ones as per the *in vivo*/*in vitro* experiment performed by Sumi et al. [103], (D) Similar behavior of NADH depicted by our computational results.

**Fig. 11:**
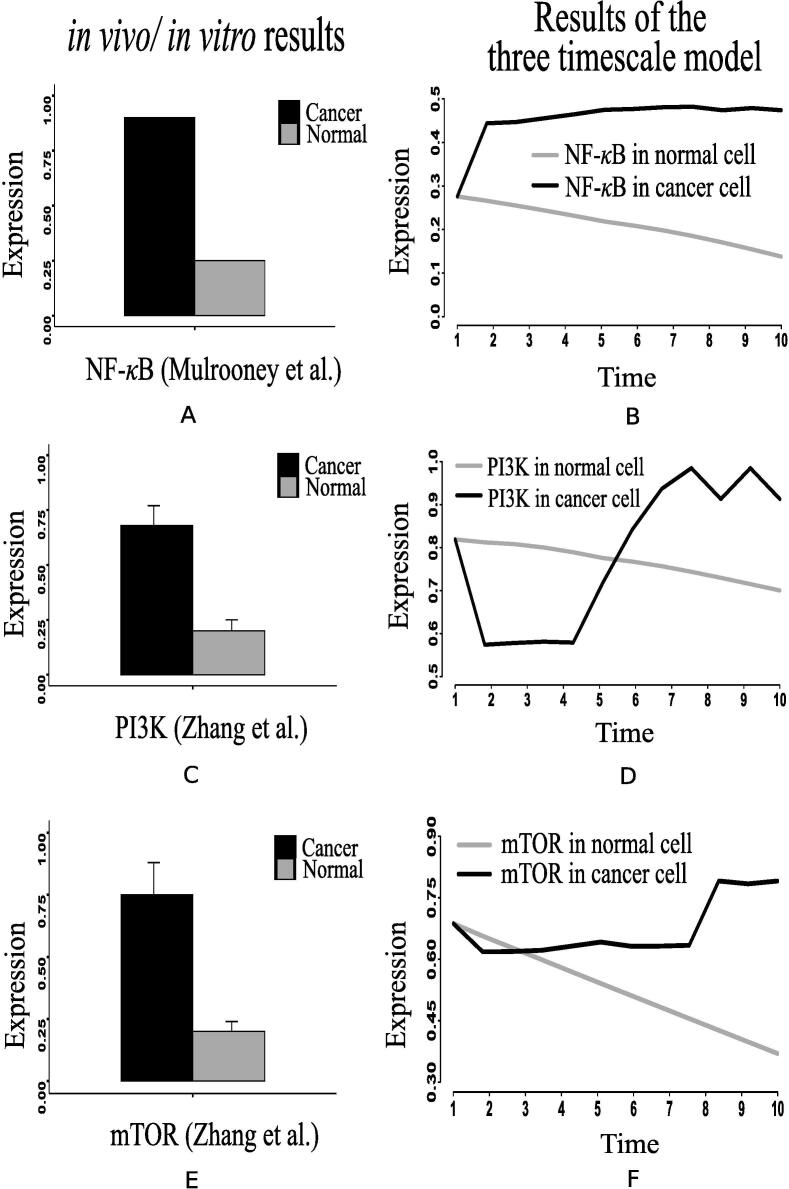
(A) Mulrooney et al. [63] has depicted that relative expression of NF-κB significantly increases in cancer (CT-2A astrocytoma) compared to normal ones. (B) Our computational results show similar behavior of NF-κB in cancer cells compared to that in normal ones. (C) Higher expression of PI3K has been captured in the *in vivo*/*in vitro* experiment performed by Zhang et al. [55] in the case of colon cancer as compared to the normal tissue samples, (D) Similar difference in the expression levels of PI3K in cancer and normal cells have been captured by the proposed computational model, (E) mTOR expression is higher in colon cancer compared to normal one as per the *in vivo*/*in vitro* experiment performed by Zhang et al. [55], and (F) Similar altered behavior of mTOR can be found in our computational results.

**Fig. 12:**
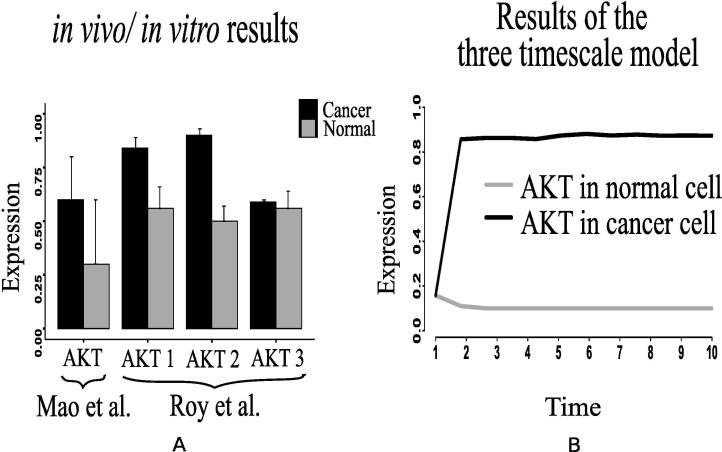
(A) Immunohistochemical scores measured by Mao et al. [56] has depicted that AKT expression is significantly higher in pancreatic cancer (91 cases) than that in normal pancreas (51 cases). Besides, Roy et al. [57] has shown the deferentially expressed AKT isoforms in normal and malignant oral tissues through immunohistochemical analysis of the human samples, (B) Similar altered behavior of AKT has been shown by the computational results generated from the proposed model.

**Fig. 13:**
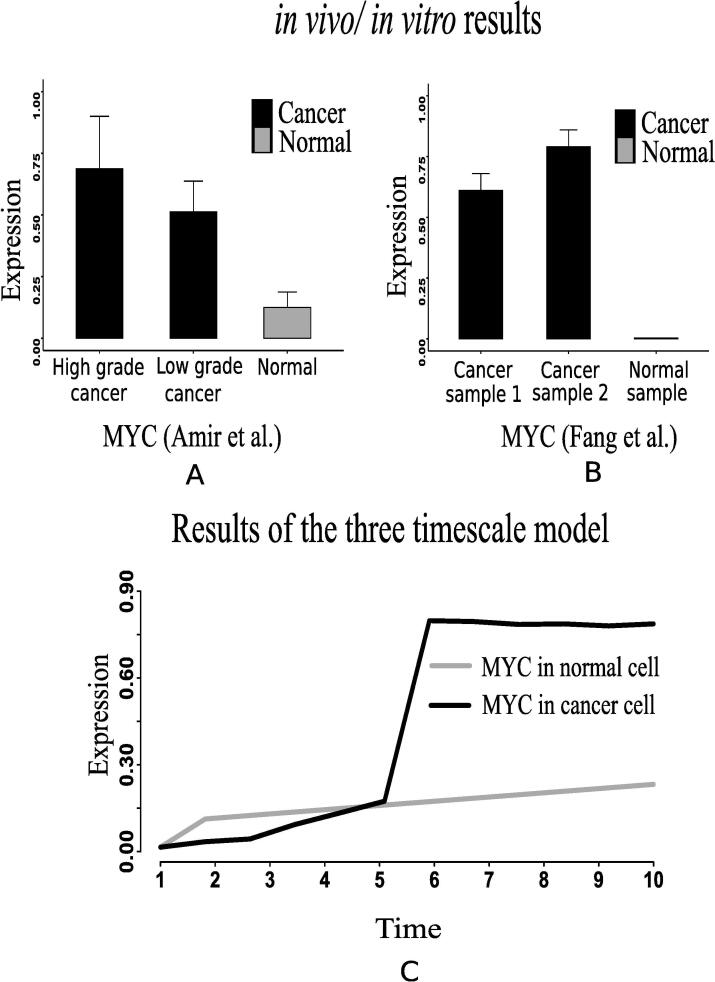
(A) & (B) depict that the relative m-RNA level protein expression [58], [59] of MYC is significantly higher in cancer cell than normal ones. (C) depicts a similar behavior of MYC generated by the proposed computational model.

**Fig. 14:**
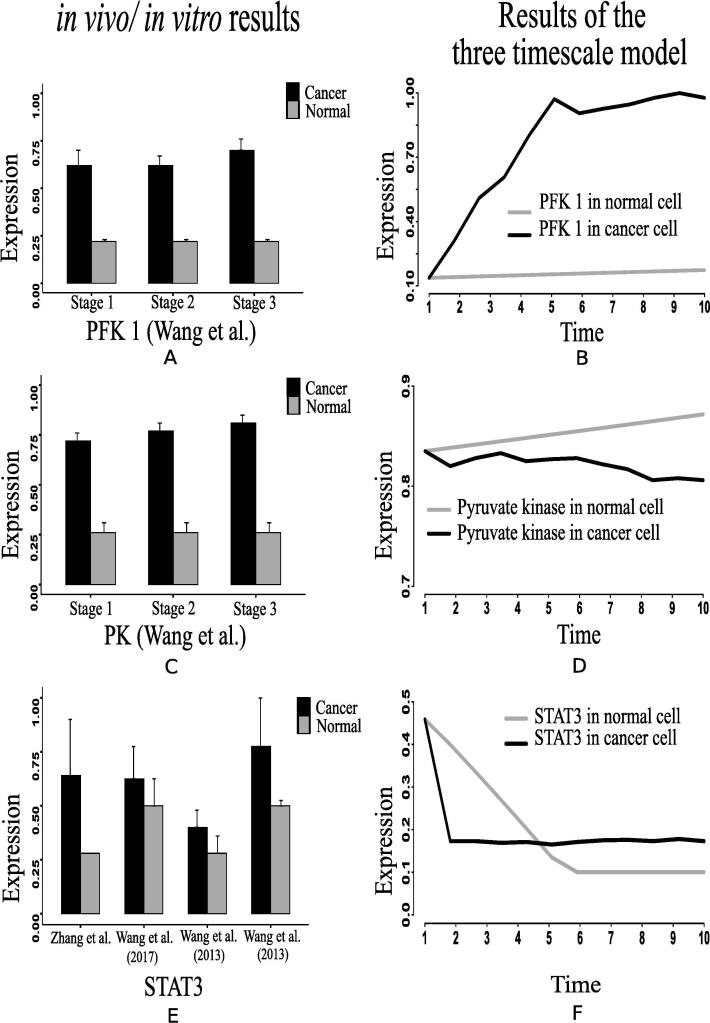
Activities of (A) PFK1 [104] and (C) pyruvate kinase in breast cancer and paracancer tissues, expressed as units per gram of protein (U/gprot) [104]. Such altered behaviors of (B) PFK1 and (D) pyruvate kinase have been captured by the proposed computational model. The previous *in vivo/ in vitro* studies have illustrated that (E) STAT3 is over expressed in cancer cells, such as breast cancer [60], gastric cancer [61] and colon cancer [62] cells compared to normal ones. Similar observation regarding (F) STAT3 can be found in our computational results.

**Fig. 15:**
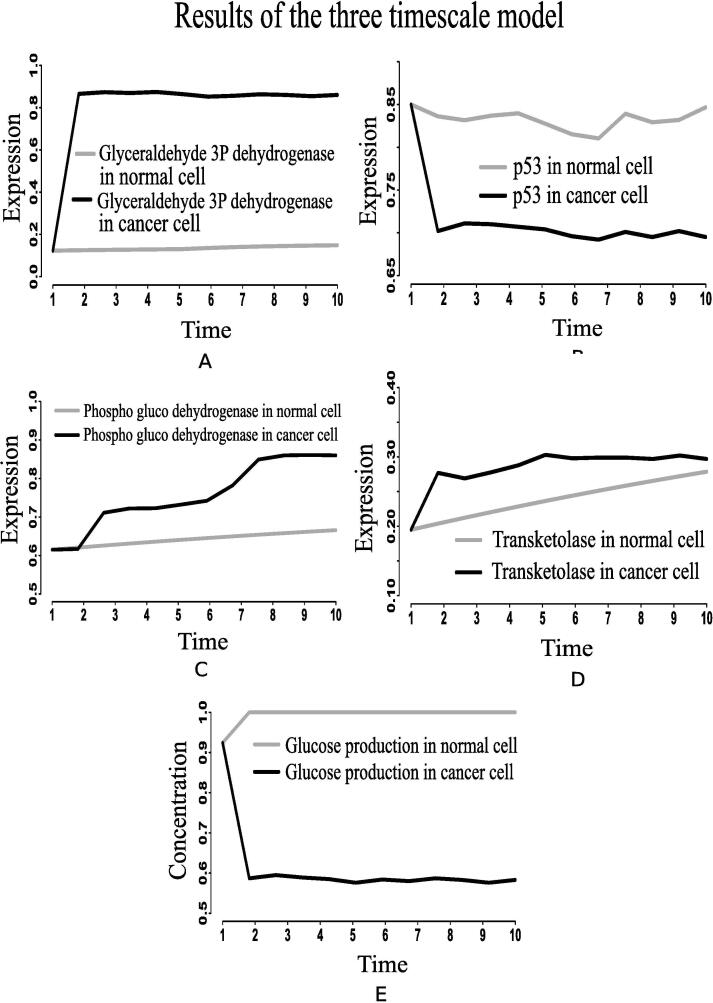
Alterations of (A) glyceraldehyde-3-phosphate dehydrogenase, (B) p53, (C) phospho-gluco dehydrogenase, (D) transketolase, and (E) glucose production in the integrated biochemical pathway related to carbon metabolism in mammalian cancer cells compared to that in normal ones.

Here, we have considered the integrated CCM pathway with incorporation of possible mutation responsible for energy management in cancer cells. The necessary pathway and information have been obtained from KEGG database [23] and literature. As a result, oncogenic somatic and germline mutation have automatically been taken care. The simulation results have found that ATP and ribose 5P production in mammalian cancer cells becomes sufficiently higher than in normal ones following the reference outputs to GA controller (Fig. 8, Fig. 8D). In this situation, we have monitored the altered behavior of other molecules that assist cancer cells to survive and grow. We have found that the expression level of PHD decreases significantly (Fig. 9E) compared to that in normal cells. As PHD accelerates the degradation of hypoxia-inducible factor-1α (HIF-1α) [108], [78], the expression level of HIF-1α is quite higher (Fig. 10B) due to less expressed PHD. Subsequently, the expression levels of phosphatidylinositol-4,5-bisphosphate 3-kinase (PI3K) (Fig. 11D), AKT (Fig. 12B), mammalian target of rapamycin (mTOR) (Fig. 11F), MYC (Fig. 13C), and extracellular signal-regulated kinases (ERK) (Fig. 9F) increase significantly in cancer cells compared to that in normal ones. Besides, signal transducer and activator of transcription 3 (STAT3) and nuclear factor kappa-light-chain-enhancer of activated B cells (NF-κB) are highly expressed (Fig. 14, Fig. 11B). On the other hand, p53 (Fig. 15B) shows under expression compared to normal mammalian cells.

In support of our simulation results, we have found some previous investigations, including *in vivo* and *in vitro* experiments [28], [29], [30], [31], [32], [33], [34], [35], [36], [37], [38], [39], [40], [41], [42], [43], [44], [64], [65], which show similar kinds of behavior. Besides, we have collected the recent *in vivo*/*in vitro* data [54], [56], [57], [55], [58], [59], [60], [61], [62], [63] of human cancer cells to compare different mutations verified in experimental laboratories with our simulation results. Here, we have normalized the experimental data in [0,1]. According to a clinical study [28], the data, collected from patients with breast, cervical and endometrial cancers at early stage, show high mortality rate of the patients having tumors with over expressed HIF-1α transcription factor. PHD inhibition promotes more stabilization of HIF-1α in cancer cells than in normal ones [29], [30]. In this context, Kwon et al. [54] has found higher expression level of HIF-1α measured in human hepatocellular carcinoma (HCC) specimens compared to the non-cancerous tissue specimens (Fig. 10A). Previous studies [31], [32], [33] have shown that PI3K, AKT and mTOR express significantly higher in tumor tissues of patients suffering from ovarian, gastric and prostate cancer compared to normal individuals. Experiment [55] with human colon cancer sample has depicted higher expression of PI3K compared to normal ones (Fig. 11C). Immunohistochemical scores measured by Mao et al. [56] has depicted that AKT expression is significantly higher in pancreatic cancer (91 cases) than that in normal pancreas (51 cases) (Fig. 12A). Besides, Roy et al. [57] has shown the deferentially expressed AKT isoforms in normal and malignant oral tissues through immunohistochemical analysis of the human samples (Fig. 12A). On the other hand, higher mTOR expression has been found in colon cancer compared to normal one as per the *in vivo*/*in vitro* experiment performed by Zhang et al. [55] (Fig. 11E).

Evidences [34], [35], [65] suggest that over expression of MYC triggers certain genes to promote growth and proliferation of cancer cells. Similar behavior of MYC in cancer cells has been found in two recent *in vivo*/*in vitro* experiments [58], [59] (Fig. 13, Fig. 13B). It is reported that tumor suppressor p53 is under expressed [36] in cancer cells due to lysine methylation [37]. Moreover, evidences [38], [39], [40] have shown that highly expressed ERK in cancer cells promotes over expression of N-cadher protein involved in metastasis. Subsequently, previous investigations [41], [42] have demonstrated that enhanced expression of STAT3 signaling protein either inhibits apoptosis or accelerates cell proliferation, angiogenesis, and finally metastasis. Consequently, it leads to cancer initiation and progression. Besides, over expression of NF-κB plays an important role in this context [39], [43], [44]. The previous *in vivo*/*in vitro* studies have supported the aforementioned claim by illustrating over expressed STAT3 in certain cancer cells, such as breast cancer [60], gastric cancer [61] and colon cancer [62] cells compared to normal ones (Fig. 14E). Besides, the evidence [63] has also depicted that relative expression of NF-κB significantly increases in cancer cells (CT-2A astrocytoma) compared to normal ones (Fig. 11A).

**Fig. 16:**
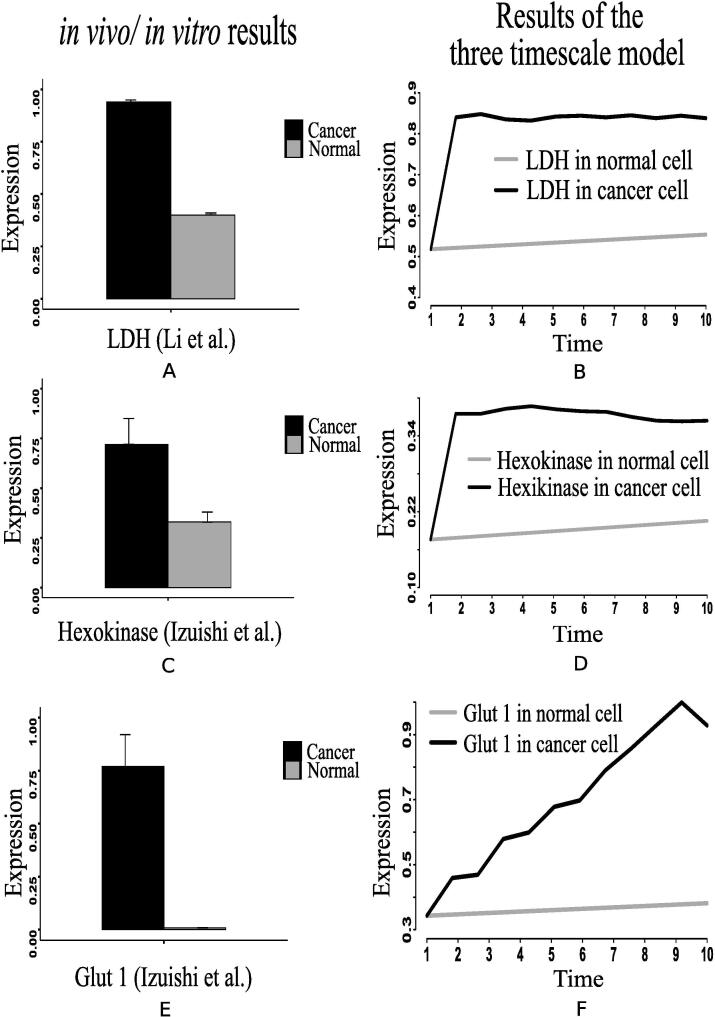
Li et al. [105] has shown that (A) LDH has significantly been up regulated in Dppa4 overexpressed cancer cell, (C) Expression of hexokinase (HK) mRNA levels have been found higher in cancer [106] compared to normal liver tissue, (E) Higher glut1 mRNA levels has been found as 92-fold higher in Meta specimens [106] compared to normal liver tissue. Similar altered behaviors of (B) LDH, (D) HK and (F) glut1 have been found in our computational results.

**Fig. 17:**
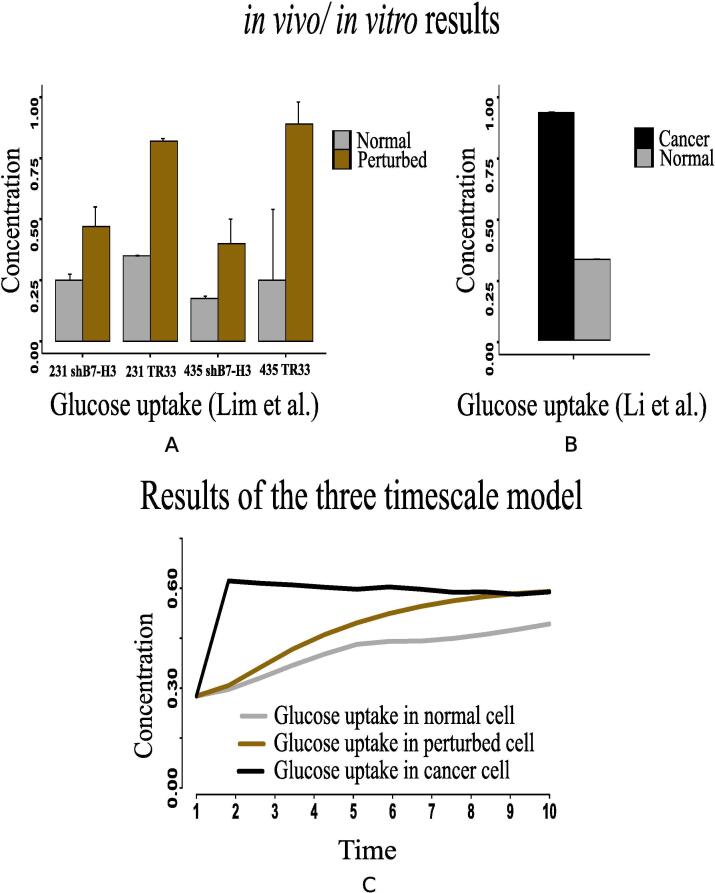
(A) Higher glucose uptake has been measured by Lim et al. [107] in B7-H3 knockdown cells grown in normoxic or perturbed conditions (hypoxia) for 24 h, (B) Li et al. [105] has shown that glucose uptake increases significantly in Dppa4 overexpressed cancer cell, and (C) Our computational results depict similar behavior of glucose uptake in cancer cells compared to that in normal and perturbed ones.

**Fig. 18:**
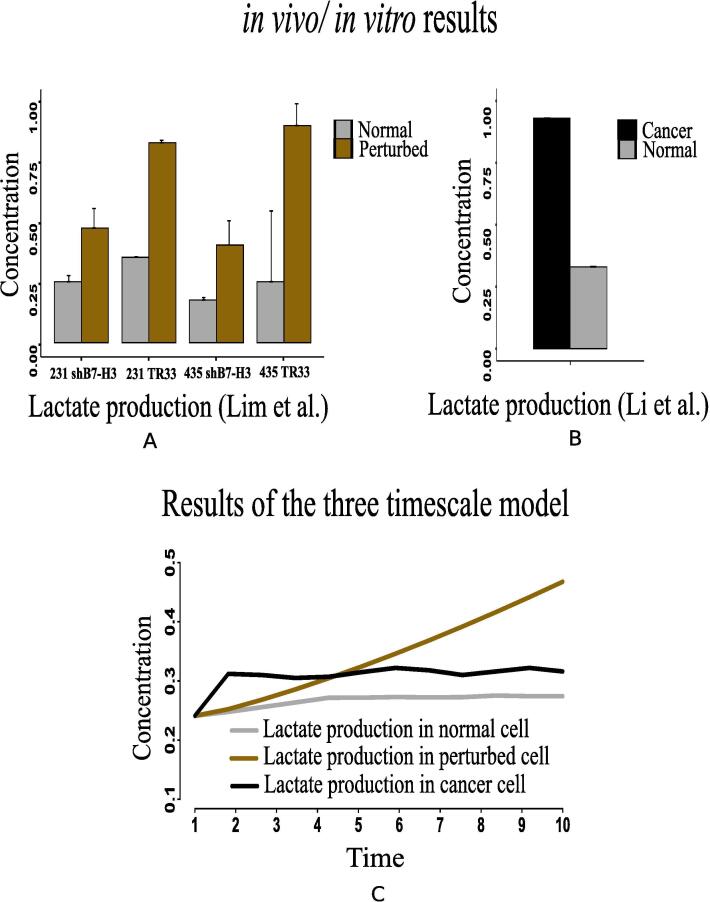
(A) Higher Lactate production has been captured by the measurement of Lim et al. [107] in B7-H3 knockdown cells grown in normoxic or perturbed conditions (hypoxia) for 24 h. (B) Higher lactate production has been shown by Li et al. [105] in Dppa4 overexpressed cancer cell, and (C) Similar altered behavior of lactate production has been depicted by our computational results in cancer cells compared to that in normal and perturbed ones.

The present simulation results demonstrate higher expression of hexokinase (HK) and glut1 (Figs. 16D and F) in cancer cells. Previous *in vivo*/*in vitro* result [106] depicting higher expression of HK mRNA levels (Fig. 16C) in cancer compared to normal liver tissue has validated the computational result. This experiment [106] has also shown higher glut1 mRNA levels (Fig. 16E) as 92-fold higher in Meta specimens compared to normal liver tissue. Here, enhanced expressions of HIF-1α, PI3K, AKT, MYC and mTOR are responsible for activating HK and glut1 more in cancer cells. As a result, glucose consumption increases (Fig. 17C). Consequently, glucose production decreases as depicted in Fig. 15E. Previous investigations [27], [45] support our claim. They have claimed that enhanced glut1 increases the utilization of glucose by anabolic pathways. Besides, highly expressed MYC activates lactate dehydrogenase (LDH) more in cancer cells. It leads to fermentation of glucose [109], [25] through enhanced glycolysis [46]. Consequently, lactate production increases. The present results have followed the above claims by showing higher expression of LDH (Fig. 16B) as well as enhanced lactate production (Fig. 18C). In this context, Lim et al. [107] has measured higher glucose uptake (Fig. 17A) in B7-H3 knockdown cells grown in normoxic or perturbed conditions (hypoxia) for 24 h. Besides, Li et al. [105] has shown that glucose uptake increases significantly in Dppa4 overexpressed cancer cells (Fig. 17B). This experiment [105] has also shown that LDH has significantly been up regulated (Fig. 16A) in Dppa4 overexpressed cancer cells. As a result, higher lactate production (Fig. 18B) has been found here [105]. Lim et al. [107] has also found similar higher lactate production (Fig. 18A) in B7-H3 knockdown cells as mentioned before. These experimental results provide a strong support to the present computational results.

Higher expression levels of phosphofructokinase 1 (PFK1) (Fig. 14B), phosphofructokinase 2 (PFK2) (Fig. 9A), and glyceraldehyde-3-phosphate dehydrogenase (Fig. 15A) also confirm enhanced glycolysis in mammalian cancer cells. In support of the computational results, a previous *in vivo*/*in vitro* result [104] has depicted higher PFK1 expression (Fig. 14A) in breast cancer and paracancer tissues, expressed as units per gram of protein (U/gprot). Consequently, higher amount of fructose 6P is utilized to produce more fructose 2,6 bisphosphate and fructose 1,6 bisphosphate than in normal cells. Besides, higher amount of fructose 2,6 bisphosphate accelerates the break down of fructose 6P into fructose 1,6 bisphosphate [96]. That is why simulation result has shown reduction of fructose 6P production (Fig. 8B). Similarly, the concentrations of PEP drops (Fig. 8E) due to its over consumption to produce higher amount of pyruvate and ATP (Fig. 8, Fig. 8A) compared to normal cells. Here, expression of pyruvate kinase switches alternatively from low to high and vice versa as depicted in Fig. 14D. Evidences [47], [48], [49], [64] have shown that pyruvate kinase (M2 isoform) switches to its inactive dimer form or active tetrameric form according to the requirements of mammalian cancer cells. When pyruvate kinase (M2 isoform) is in tetrameric form, the flux through glycolysis enhances with sufficient amount of ATP production. As a result, production of intermediate glycolytic metabolites, such as glyceraldehyde 3P (Fig. 8C and fructose 1,6 bisphosphate, increases. Conversely, dimer form of pyruvate kinase (M2 isoform) promotes macromolecular synthesis from glycolytic intermediate metabolites to continue cell growth and proliferation. An evidence [104] has also reported about higher pyruvate kinase expression (Fig. 14C) in breast cancer and paracancer tissues, expressed as units per gram of protein (U/gprot). Thus, cancer cells manage energy in the form of ATP to survive in spite of slow oxidative phosphorylation under consideration. Here, reduction of reduced nicotinamide adenine dinucleotide (NADH) production (Fig. 10D) indicates successful incorporation of slow oxidative phosphorylation [50] into the proposed model. An *in vivo*/*in vitro* experiment, performed by Sumi et al. [103] depicting lower concentration of NADH (Fig. 10C) in cancer cells compared to normar ones, supports the computational result.

According to the simulation results, the enzymes (proteins) catalyzing PPP, such as glucose-6-phosphate dehydrogenase (Fig. 9D), phospho-gluco dehydrogenase (Fig. 15C), ribose 5P isomerase (Fig. 9C), transketolase and transaldolase (Fig. 15D and 9B), have shown over expression in cancer cells for macromolecular precursors required for cell growth and proliferation. Under expressed p53 plays an important role in this context. High concentration of ribose 5P (Fig. 8D) confirms enhanced production of cell building material, including DNA, RNA, nucleic acids and histidine, in mammalian cancer cells. Evidences [51], [52], [53] have shown that relatively higher expressions of glucose-6-phosphate dehydrogenase, phospho-gluco dehydrogenase, ribose 5P isomerase, transketolase and transaldolase help cancer cells in generating high amount of NADPH and ribose 5P, which are responsible for reactive oxygen species (ROS) reduction as well as the production of high levels of nucleotides for DNA synthesis and repair. It leads to resistance against certain cancer therapies resulting in enhancement of oxidative stress or DNA damage. In this context, it should be mentioned that higher amount of ROS can frequently be observed in cancer cells helping in activation of oncogenes and metastasis. However, further enhancement of ROS beyond a certain threshold induces cell death [110]. Finally, we have summarized the altered regulations of different metabolites, transcription factors and genes in cancer cells compared to that in normal ones in Table 3.

### 3.3 Analysis of certain rational drug targets in terms of management of energy and cell proliferation in mammalian cancer cells

Here, we are going to discuss the effects of certain rational drug targets on cancer cells in terms of management of energy and cell proliferation from simulation point of view. These results may have significant implications during *in vivo* and/or *in vitro* experiments. When the proposed model computationally meets the reference target concentrations of ATP and ribose 5P for GA controller, we have observed that the model has mimicked the altered regulation of cancer cells as previously discussed. At that moment, we have set reference target expression levels (high or low) of certain proteins/enzymes as drug targets using GA controller. Although the model is quite capable of analyzing the effect of any drug target, we have considered only six drug targets among others for our study to restrict the size of present article. Here, we have monitored the effects of deactivating pyruvate kinase (reference expression level 0.02), glucose-6-phosphate dehydrogenase (reference expression level 0.02), transketolase (reference expression level 0.09), ribose 5P isomerase (reference expression level 0.09) and glucose-6-phosphate isomerase (reference expression level 0.03). Finally, the effect of activating pyruvate kinase (reference expression level 0.98) has also been observed and analyzed. Table 4 summarizes the significance of the drug targets under consideration in energy supply and cell proliferation.

**Fig. 19:**
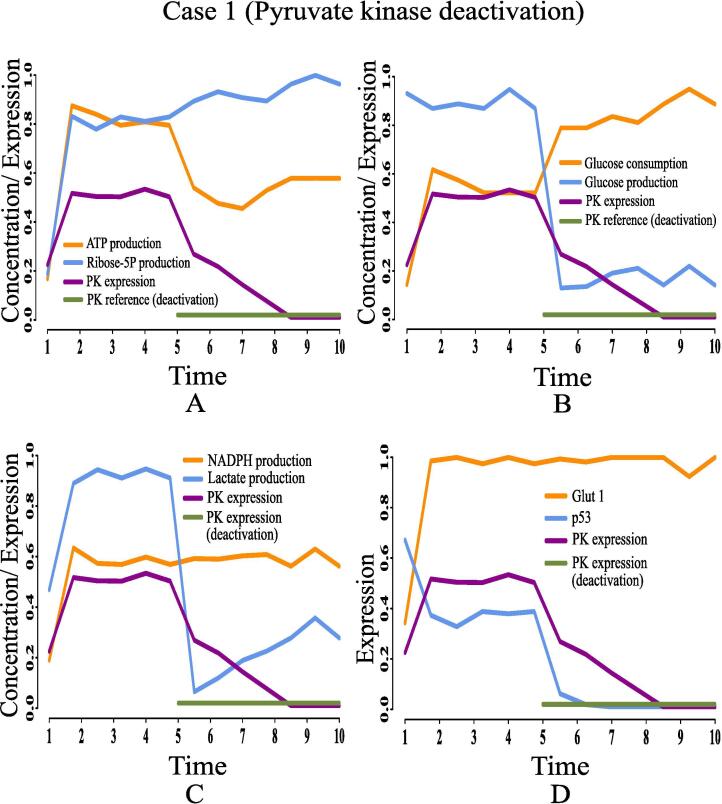
Case 1: Deactivation of pyruvate kinase as a drug target. After time labeled 5.0 along X-axis, effects of pyruvate kinase deactivation can be noticed.
• **Case 2 (Glucose-6-phosphate dehydrogenase deactivation):** Glucose-6-phosphate dehydrogenase is the key enzyme (protein) to utilize G6P through PPP. It leads to producing high amount of NADPH and ribose 5P so that nucleotides and fatty acid can sufficiently be synthesized to maintain intracellular redox homeostasis [112]. The present results (Fig. 20) have shown that the concentrations of ribose 5P (Fig. 20A) and NADPH (Fig. 20, Fig. 21B) decrease due to deactivation of glucose-6-phosphate dehydrogenase. In support of this result, a recent *in vivo*/*in vitro* study has shown how the relative activity (expression level) of glucose-6-phosphate dehydrogenase enzyme controls the NADPH production [111]. Lower expression of glucose-6-phosphate dehydrogenase leads to reduction of the concentration of NADPH (Fig. 21A). Here, expression of glucose-6-phosphate dehydrogenase and NADPH concentration have been assessed in A549/DDP cells according to various concentrations of 6-Aminonicotinamide (6-AN). Besides, expression level of p53 increases (Fig. 20D), whereas glut1 expression decreases (Fig. 20D). These results indicate reduction of cell growth and proliferation due to glucose-6-phosphate dehydrogenase deactivation. However, high amount of glucose (Fig. 20B) is utilized through glycolysis to generate sufficient ATP (Fig. 20A) for cell survival. Subsequently, cell fermentation continues through high amount of lactate production (Fig. 20C). A previous study [113] has shown reduction of cell proliferation by silencing glucose-6-phosphate dehydrogenase in the human breast cancer cell line MCF7. Thus, deactivation of glucose-6-phosphate dehydrogenase as a possible drug target may be efficient to reduce cell growth and proliferation but not be effective in reduction of energy supply and fermentation.

• **Case 1 (Pyruvate kinase deactivation):** According to the simulation results as depicted in Fig. 19, we have found that deactivation of pyruvate kinase cannot reduce the concentration of ribose 5P (Fig. 19A) and NADPH (Fig. 19C). In other words, high proliferation of mammalian cancer cells continues. Besides, enhanced NADPH still prevents from ROS production helping cancer cells to survive in spite of oxidative stress. Although the concentrations of ATP (Fig. 19A) and lactate (Fig. 19C) reduce at the beginning, after a while they increase again significantly. Even pyruvate kinase deactivation cannot reduce high glucose utilization (*i.e.*, high consumption and less production) (Fig. 19B) in mammalian cancer cells. Moreover, high expression of glut1 and under expression of tumor suppression protein p53 (Fig. 19D) cannot be reversed in this case. Thus, pyruvate kinase deactivation may not be a good choice as a drug target for mammalian cancer therapy.

**Fig. 20:**
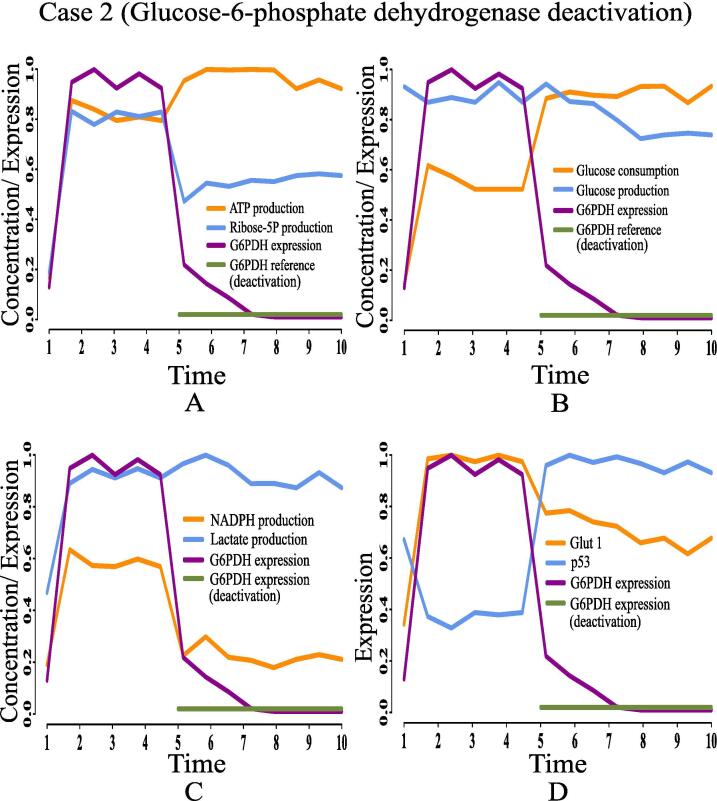
Case 2: Deactivation of glucose-6-phosphate dehydrogenase as a drug target. After time labeled 5.0 along X-axis, effects of glucose-6-phosphate dehydrogenase deactivation can be noticed.

**Fig. 21:**
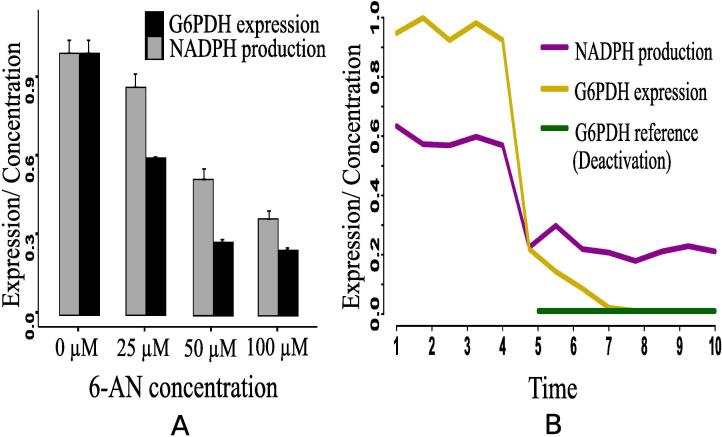
Effect of glucose-6-phosphate dehydrogenase deactivation (case 2): (A) The relative enzymatic activity (expression level) of glucose-6-phosphate dehydrogenase controls the NADPH production [111]. Lower expression of glucose-6-phosphate dehydrogenase leads to reduce the concentration of NADPH. Here, expression of glucose-6-phosphate dehydrogenase and NADPH concentration have been assessed in A549/DDP cells according to various concentrations of 6-Aminonicotinamide (6-AN) (unit in μM), (B) The proposed computational model depicts similar behavior of NADPH due to the deactivation of glucose-6-phosphate dehydrogenase.
• **Case 3 (Transketolase deactivation):** Transketolase has similar importance as glucose 6-phosphate dehydrogenase to maintain cell growth and proliferation leading to metastasis [114]. Simulation results (Fig. 22) demonstrate that the concentration of ribose 5P (Fig. 22A) decreases significantly in accordance with the deactivation of transketolase. Subsequently, enhanced expression of p53 (Fig. 22D) conveys that cell growth and proliferation are inhibited in this case. Reduction of NADPH concentration (Fig. 22C) signifies enhancement of ROS preventing from survival of cancer cells by oxidative stress. Moreover, ATP (Fig. 22A) and lactate (Fig. 22C) production also decrease through reduced glucose utilization (Fig. 22B). Besides, expression level of glut1 decreases as depicted in Fig. 22D. Previous investigations [113], [115], [116] have shown similar effects due to transketolase silencing. Thus, it is clear that targeting transketolase may be a potential choice for future cancer therapy.

**Fig. 22:**
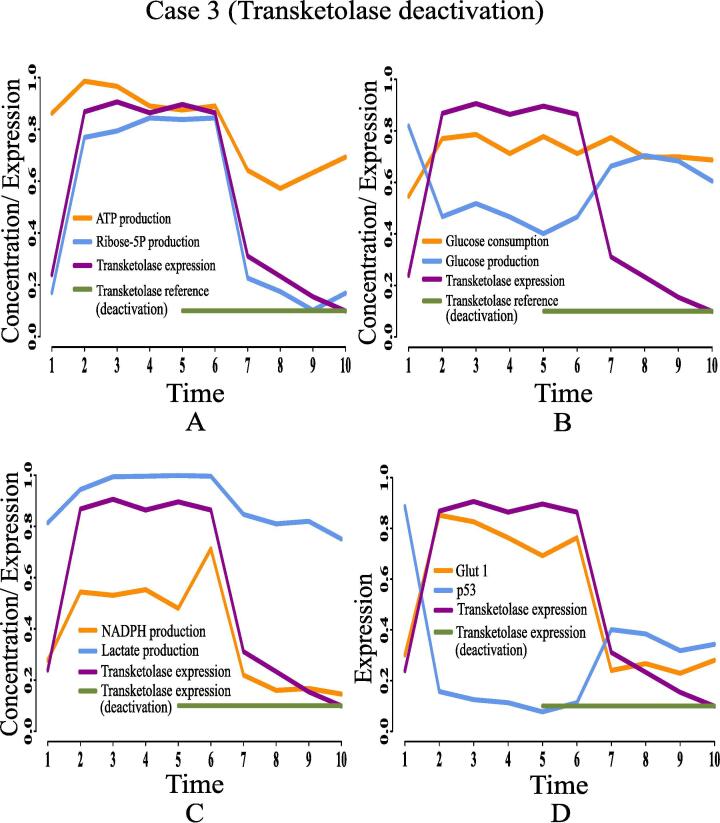
Case 3: Deactivation of transketolase as a drug target. After time labeled 5.0 along X-axis, effects of transketolase deactivation can be noticed.
• **Case 4 (Ribose 5P isomerase deactivation):** We have already discussed that up regulation of ribose 5P isomerase plays an important role in cell growth and proliferation of a cancer patient. A previous study [117] has claimed that expression level of ribose 5P isomerase is enhanced in colorectal cancer. Our simulation results (Fig. 23) have shown the effect of silencing ribose 5P isomerase in cancer cells. We have observed that enhanced p53 expression and decreased glut1 expression (Fig. 23D) indicate slowing down of proliferation and growth of cancer cells. In this context, reduction in NADPH (Fig. 23C) and ribose 5P (Fig. 23A) concentration confirm our claim. Subsequently, glucose utilization (Fig. 23B) and cell fermentation (*i.e.*, lactate production as depicted in Fig. 23C) decrease. However, energy supply in the form of ATP (Fig. 23A) in cancer cells is somehow managed in spite of the deactivation of ribose 5P isomerase. Consequently, ribose 5P isomerase might be considered as a biomarker for targeted cancer therapy and prediction.

**Fig. 23:**
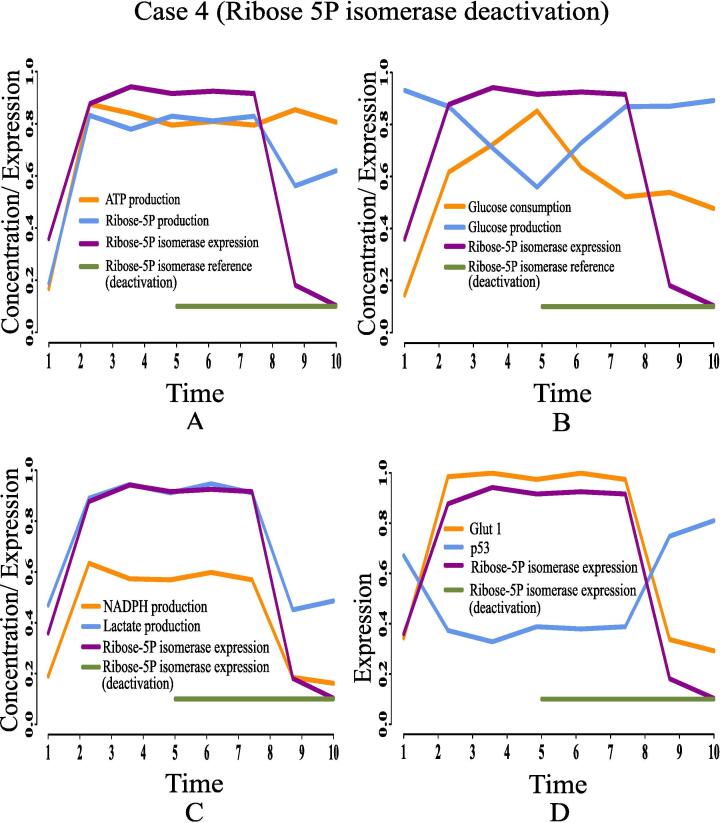
Case 4: Deactivation of ribose 5P isomerase as a drug target. After time labeled 5.0 along X-axis, effects of ribose 5P isomerase deactivation can be noticed.
• **Case 5 (Glucose-6-phosphate isomerase deactivation):** Glucose-6-phosphate isomerase or phosphoglucose isomerase (PGI) is a glycolytic enzyme that directs G6P flux into the glycolysis branch. As a result, G6P breaks down into fructose 6P. Deactivation of Glucose-6-phosphate isomerase results in slowing down of glucose utilization (Fig. 24B) as well as lactate (Fig. 24C) and ATP (Fig. 24A) production. Thus “Warburg effect” is somehow reversed. Besides, reduced glut1 expression level along with enhanced p53 expression (Fig. 24D) may slow down growth and proliferation rate of cancer cells. In this context, reduction of NADPH (Fig. 24C) and ribose 5P (Fig. 24A) concentration supports our claim. Here, a previous investigation [118] has shown that down regulation of Glucose-6-phosphate isomerase may suppress “Warburg effect”. Besides, oxidative phosphorylation is activated. However, its impact on tumor growth is minimal except in the case of hypoxia. Thus, glucose-6-phosphate isomerase can be a potential drug target for future cancer therapy.

**Fig. 24:**
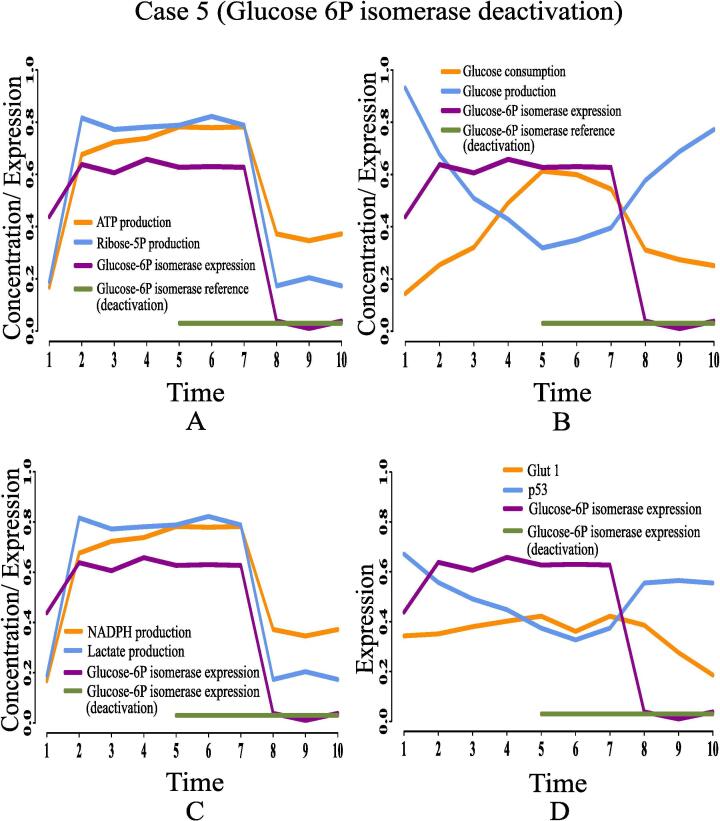
Case 5: Deactivation of glucose-6-phosphate isomerase as a drug target. After time labeled 5.0 along X-axis, effects of glucose-6-phosphate isomerase deactivation can be noticed.
• **Case 6 (Pyruvate kinase activation):** Activation of pyruvate kinase (Fig. 25) may slow down cell growth and proliferation as well as cell fermentation. Decreased concentrations of NADPH, lactate (Fig. 25C) and ribose 5P (Fig. 25A) confirm our claim. Besides, decreased glucose utilization (Fig. 25B) and glut1 expression (Fig. 25D) support the fact. Here, expression level of p53 (Fig. 25D) also increases to slow down cell growth and proliferation. However, ATP is produced sufficiently in this case as depicted in Fig. 25A. Evidence [47] has claimed that cancer cell growth and proliferation are inhibited by activation of pyruvate kinase (M2 isoform). Thus, pyruvate kinase (M2 isoform) activators may be considered as possible significant drugs for oncogenic treatment.

**Fig. 25:**
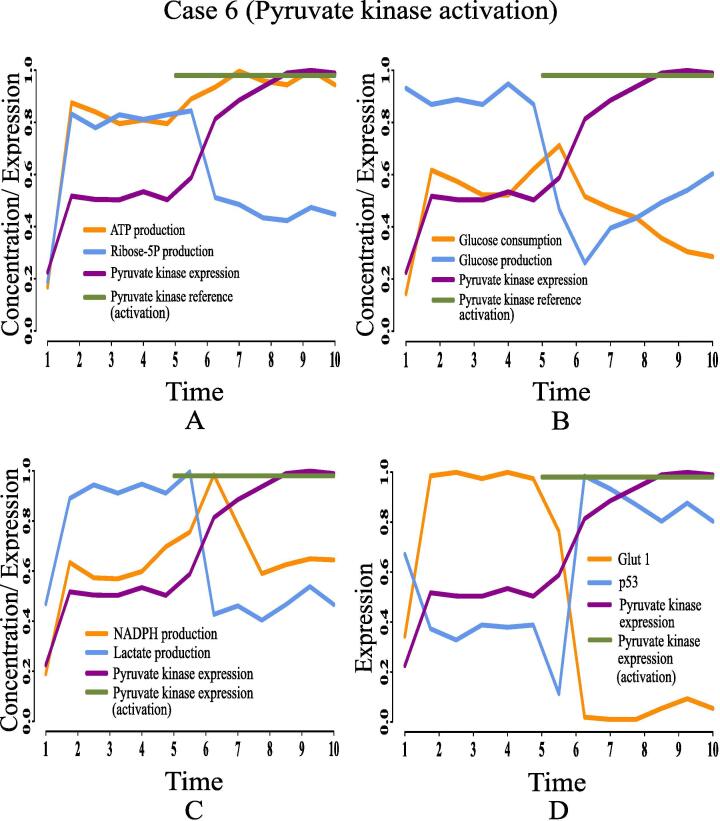
Case 6: Activation of pyruvate kinase as a drug target. After time labeled 5.0 along X-axis, effects of pyruvate kinase activation can be noticed.

**Table 4:** Illustrating the significance of certain rational drug targets in terms of management of energy and cell proliferation in mammalian cancer cells.

Drug target	Energy (ATP) production	Cell proliferation indicated by the production of ribose 5P and NADPH	Glucose utilization	p53 expression	Glut1 expression	Remarks	References for validation
Deactivation of pyruvate kinase	Moderately decreases	Higher production of ribose 5P and NADPH continue	Significantly increases	Significantly decreases	No significant change	Probably not a good choice	-
Deactivation of glucose-6-phosphate dehydrogenase	Minutely increases	Both ribose 5P and NADPH significantly decrease	Increases	Significantly increases	Decreases	May be efficient to reduce cell proliferation	[113], [111]
Deactivation of transketolase	Moderately decreases	Both ribose 5P and NADPH significantly decrease	No significant change	Moderately increases	Significantly decreases	Probable significant	[113], [114], [115], [116]
Deactivation of ribose 5P isomerase	No significant change	Both ribose 5P and NADPH decrease	Moderately decreases	Significantly increases	Significantly decreases	May be a good choice	[117]
Deactivation of glucose-6-phosphate isomerase	Significantly decreases	Both ribose 5P and NADPH significantly decrease	Decreases	Increases	Decreases	Probable effective choice	[118]
Activation of pyruvate kinase	Moderately increases	Both ribose 5P and NADPH significantly decrease	Moderately decreases	Significantly increases	Significantly decreases	May be efficient to reduce cancer progression	[47]

## 4 Conclusion

In this study, we have successfully integrated three types of biochemical pathways, *viz.*, metabolic, signaling and gene regulatory networks, keeping their three timescale nature intact. Here, we have developed the integrated state equations considering appropriate timescales as well as all possible perturbations present in the contemplated integrated biochemical pathway. Besides, depending on the training dataset generated by solving the pathway ODEs, SVR based MIMO model has been developed. The MIMO model can mimic the transient nonlinear dynamic behavior of the integrated biochemical pathway under consideration. Moreover, with the help of the GA controller, the model can predict the effect of drug targets applied to complex diseased cells. In order to investigate the effectiveness of the model, we have used our model to explore how mammalian cancer cells are able to manage their growth, proliferation and energy supply to survive. In this context, “Warburg effect” [24], [25] has been taken into account. The simulation results have depicted that the model has not only captured the key regulations, but also has been able to predict certain possible drug effects in terms of energy and cell proliferation management in mammalian cancer cells.

According to the results, the proteins or genes HIF-1α, HK, glut1, AKT, glyceraldehyde-3-phosphate dehydrogenase, phospho-gluco dehydrogenase, ERK, ribose 5P isomerase, mTOR, glucose-6-phosphate dehydrogenase, STAT3, NF-κB, PI3K, MYC, LDH, PFK1, PFK2, transketolase and transaldolase are up regulated in cancer cells. Besides, PHD and p53 are down regulated. Switching of pyruvate kinase (M2 isoform) between its two oligomeric form, *viz.*, inactive dimer and active tetramer, plays an important role in managing proliferation, growth and energy in mammalian cancer cells. These results have been validated through previous investigations involving *in vivo* and *in vitro* experiments [24], [27], [26], [28], [29], [30], [31], [32], [33], [34], [35], [36], [37], [38], [39], [40], [41], [42], [43], [44], [45], [25], [46], [47], [48], [49], [50], [51], [52], [53], [54], [56], [57], [55], [58], [59], [60], [61], [62], [63], [64], [65]. Besides, other mathematical models [66], [67], [68], [69] support the results derived by the proposed model. Among six drug targets under consideration, deactivation of transketolase and glucose-6-phosphate isomerase may be the most potential to slow down cancer progression by reducing cell proliferation, growth, fermentation and energy supply. On the other hand, pyruvate kinase (M2 isoform) activation and ribose 5P isomerase deactivation may reduce cell growth, proliferation and fermentation during cancer. However, they may not be able to stop energy supply in mammalian cancer cells. Although deactivation of glucose-6-phosphate dehydrogenase may slow down cell growth and proliferation, it may fail to stop fermentation and energy supply in the malignant cells. In this context, pyruvate kinase deactivation may be an awful choice as a rational drug target for future cancer therapy.

Finally, it may be mentioned that the experimental values of different kinetic parameters are still unknown or poorly documented. In this study, we have estimated these values by trial and error based on previous knowledgebase. Some of the parameter values considered here have been seen to be close enough with those estimated by the method of Lillacci et al. [70]. The other parameter values could not be checked due to unavailability of required experimental observations. However, trial and error based technique is time consuming and difficult to perform because of large number of such kinetic parameters. This is a drawback of the present methodology. Thus more *in vivo*/*in vitro* parameter values are needed, and/or appropriate parameter estimation methods based on the theory of machine learning and control theory can be developed for designing more accurate MIMO plant for an integrated biochemical pathway.

## Authors’ Contributions

AD and RKD initiated control theoretic modeling of metabolic pathways. AD conceptualized the basic idea of three timescale modeling. AD and AB formulated the methodology and implemented it. RKD gave crucial theoretical input. AD and AB wrote the first draft of the manuscript. RKD corrected it. NC read the article and gave fruitful suggestions to edit the manuscript.

## Funding

No funding agency has funded this work.

## Declaration of Competing Interest

The authors declare that they have no known competing financial interests or personal relationships that could have appeared to influence the work reported in this paper.
