# Supplementary material for: A control theoretic three timescale model for analyzing energy management in mammalian cancer cells
Source: Comput Struct Biotechnol J. 2020 Dec 29;19:477–508. doi: 10.1016/j.csbj.2020.12.019 (PMC7809419; doi:10.1016/j.csbj.2020.12.019)
Supplement: Supplementary file 1 [file mmc1.pdf]

A Control Theoretic Three Timescale Model for Analyzing Energy Management in Mammalian Cancer Cells

Abhijit Dasgupta [2], Abhisek Bakshi [4], Nirmalya Chowdhury [3], and Rajat K. De [1*]

[1] Machine Intelligence Unit, Indian Statistical Institute, 203 B.T. Road, Kolkata 700108, India; [2] Department of Data Science, School of Interdisciplinary Studies, University of Kalyani, Kalyani, Nadia 741235, West Bengal, India; [3] Department of Computer Science & Engineering, Jadavpur University, Kolkata 700032, India; [4] Department of Information Technology, Bengal Institute of Technology, Basanti Highway, Kolkata 700150, India

Supplementary Material

A Supplementary Tables

Table S-1 Activation and inhibition of different genes under consideration in the main article.

Gene no.	Gene name	Activated by	Inhibited by	Basal production rate	Decay rate	Feedback constants
1	glut1	HIF_1, P13K, AKT, MYC, mTOR	p53	b_{glut1}	d_{glut1}	$\mathcal{F}_1^{(g)}$
2	pgm_1	HIF_1		b_{pgm_1}	d_{pgm_1}	
3	hk	HIF_1, P13K, AKT, MYC, mTOR		b_{hk}	d_{hk}	
4	g6Pase	HIF_1	AKT, AMPK	b_{g6Pase}	d_{g6Pase}	$\mathcal{F}_2^{(g)}, \mathcal{F}_3^{(g)}$
5	pgi	HIF_1, AKT, MYC, mTOR		b_{pgi}	d_{pgi}	
6	pfk1	HIF_1, P13K, AKT, MYC, mTOR, AMPK	p53	b_{pfk1}	d_{pfk1}	$\mathcal{F}_4^{(g)}$
7	f16Bpase	ERK		$b_{f16Bpase}$	$d_{f16Bpase}$	
8	pfk2	HIF_1, P13K, MYC, AKT, AMPK	p53	b_{pfk2}	d_{pfk2}	$\mathcal{F}_5^{(g)}$
9	f26Bpase	E2F1, ERK, MYC		$b_{f26Bpase}$	$d_{f26Bpase}$	
10	ald	HIF_1, MYC, AKT, mTOR		b_{ald}	d_{ald}	
11	tpi	mTOR		b_{tpi}	d_{tpi}	
12	g3Pdh	HIF_1, MYC, AKT		b_{g3Pdh}	d_{g3Pdh}	
13	pglck	HIF_1, MYC, AKT, mTOR		b_{pglck}	d_{pglck}	
14	pglcm	HIF_1, mTOR		b_{pglcm}	d_{pglcm}	
15	enl	HIF_1, MYC		b_{enl}	d_{enl}	
16	pk	HIF_1	GF	b_{pk}	d_{pk}	$\mathcal{F}_6^{(g)}$
17	ldh	HIF_1, MYC, mTOR		b_{ldh}	d_{ldh}	
18	pyrd	MYC, AKT	HIF_1, p53	b_{pyrd}	d_{pyrd}	$\mathcal{F}_7^{(g)}, \mathcal{F}_8^{(g)}$
19	Acyl_coa_syn	ERK, MYC, STAT3		$b_{Acyl\_coa\_syn}$	$d_{Acyl\_coa\_syn}$	
20	fa_synthase	ERK, MYC, E2F1, AKT	AMPK	$b_{fa\_synthase}$	$d_{fa\_synthase}$	$\mathcal{F}_9^{(g)}$
21	pyr_crbxylase	STAT3, NF-κB, p53, MYC, E2F1		$b_{pyr\_crbxylase}$	$d_{pyr\_crbxylase}$	
22	pep_crbxykinase1	NF-κB, ERK	AKT, AMPK	$b_{pep\_crbxykinase1}$	$d_{pep\_crbxykinase1}$	$\mathcal{F}_{10}^{(g)}, \mathcal{F}_{11}^{(g)}$
23	cit_synthase	RORa		$b_{cit\_synthase}$	$d_{cit\_synthase}$	
24	actnase	HIF_1		$b_{actnase}$	$d_{actnase}$	
25	isocit_deh	MYC		b_{isocit_deh}	d_{isocit_deh}	
26	KG_deh_cmp	MYC, STAT3		$b_{KG\_deh\_cmp}$	$d_{KG\_deh\_cmp}$	
27	succ_coa_synthase	MYC, E2F1, ERK		$b_{succ\_coa\_synthase}$	$d_{succ\_coa\_synthase}$	
28	succ_deh	MYC, E2F1, p53, AKT	LPS	b_{succ_deh}	d_{succ_deh}	$\mathcal{F}_{13}^{(g)}$
29	frmase	MYC, E2F1, p53, N-κFB		b_{frmase}	d_{frmase}	
30	mal_deh	E2F1, ERK		b_{mal_deh}	d_{mal_deh}	
31	g6Pdeh	mTOR	p53	b_{g6Pdeh}	d_{g6Pdeh}	$\mathcal{F}_{12}^{(g)}$
32	pglc6	p53		b_{pglc6}	d_{pglc6}	
33	phglc_deh	p53, mTOR		b_{phglc_deh}	d_{phglc_deh}	
34	r5Piso_A	ERK, AKT		b_{r5Piso_A}	d_{r5Piso_A}	
35	rbls_5P_3ep	mTOR		$b_{rbls\_5P\_3ep}$	$d_{rbls\_5P\_3ep}$	
36	trkl	mTOR		b_{trkl}	d_{trkl}	
37	trsdl1	E2F1, STAT3, MYC		b_{trsdl1}	d_{trsdl1}	

Table S-2 State equations for the genes considered in Table S-1.

Gene no.	Final equation generated for each gene
1	glut1 = ($\mathcal{K}_1^{(g)}$ *[HIF_1]*[P13K]*[AKT]*[MYC]*[mTOR])/(1 + $\mathcal{F}_1^{(g)}$ *[p53]) + b_{glut1} - d_{glut1};
2	pgm_1 = $\mathcal{K}_2^{(g)}$ *[HIF_1] + b_{pgm_1} - d_{pgm_1};
3	hk = $\mathcal{K}_3^{(g)}$ *[HIF_1]*P13K]*[AKT]*[MYC]*[mTOR] + b_{hk} - d_{hk};
4	g6Pase = $\mathcal{K}_4^{(g)}$*[HIF_1]) / ((1 + $\mathcal{F}_2^{(g)}$*[AKT])*(1 + $\mathcal{F}_3^{(g)}$*[AMPK])) + b_{g6Pase} - d_{g6Pase};
5	pgi = $\mathcal{K}_5^{(g)}$*[HIF_1]*[MYC]*[AKT]*[mTOR] + b_{pgi} - d_{pgi};
6	pfk1 = ($\mathcal{K}_6^{(g)}$*[HIF_1]*[MYC]*[P13K]*[AKT]*[AMPK]*[mTOR])/(1 + $\mathcal{F}_4^{(g)}$*[p53]) + b_{pfk1} - d_{pfk1};
7	f16Bpase = ($\mathcal{K}_7^{(g)}$* [ERK]) + $b_{f16Bpase}$ - $d_{f16Bpase}$;
8	pfk2 = ($\mathcal{K}_8^{(g)}$*[HIF_1]*[MYC]*[P13K]*[AKT]*[AMPK])/(1 + $\mathcal{F}_5^{(g)}$*[p53]) + b_{pfk2} - d_{pfk2};
9	f26Bpase = ($\mathcal{K}_9^{(g)}$ * [E2F1]*[ERK]*[MYC]) + $b_{f26Bpase}$ - $d_{f26Bpase}$;
10	ald = $\mathcal{K}_{10}^{(g)}$*[HIF_1]*[MYC]*[AKT]*[mTOR] + b_{ald} - d_{ald};
11	tpi = $\mathcal{K}_{11}^{(g)}$*[mTOR] + b_{tpi} - d_{tpi};
12	g3Pdh = $\mathcal{K}_{12}^{(g)}$ *[HIF_1]*[MYC]*[AKT] + b_{g3Pdh} - d_{g3Pdh};
13	pglck = $\mathcal{K}_{13}^{(g)}$*[HIF_1]*[MYC]*[AKT]*[mTOR] + b_{pglck} - d_{pglck};
14	pglcm = $\mathcal{K}_{14}^{(g)}$*[HIF_1]*[mTOR] + b_{pglcm} - d_{pglcm};
15	enl = $\mathcal{K}_{15}^{(g)}$ *[HIF_1]*[MYC] + b_{enl} - d_{enl};
16	pk = ($\mathcal{K}_{16}^{(g)}$ *[HIF_1])/(1 + $\mathcal{F}_6^{(g)}$*[GF]) + b_{pk} - d_{pk};
17	ldh = $\mathcal{K}_{17}^{(g)}$*[HIF_1]*[MYC]*[mTOR]+ b_{ldh} - d_{ldh};
18	pyrd = ($\mathcal{K}_{18}^{(g)}$ * [MYC]*[AKT])/((1+ $\mathcal{F}_7^{(g)}$*[HIF_1])*(1+ $\mathcal{F}_8^{(g)}$*[p53])) + b_{pyrd} - d_{pyrd};
19	Acyl_coa_syn = ($\mathcal{K}_{19}^{(g)}$*[ERK]*[MYC]*[STAT3]) + $b_{Acyl\_coa\_syn}$ - $d_{Acyl\_coa\_syn}$;
20	fa_synthase = ($\mathcal{K}_{20}^{(g)}$* [ERK]*[MYC]*[E2F1]*[AKT])/(1+ $\mathcal{F}_9^{(g)}$*[AMPK]) + $b_{fa\_synthase}$ - $d_{fa\_synthase}$;
21	pyr_crbxylase = ($\mathcal{K}_{21}^{(g)}$*[STAT3]*[NF-κB]*[p53]*[MYC]*[E2F1]) + $b_{pyr\_crbxylase}$ - $d_{pyr\_crbxylase}$;
22	pep_crbxykinase1 = ($\mathcal{K}_{22}^{(g)}$ *[NF-κB]*[ERK]) /((1+ $\mathcal{F}_{10}^{(g)}$*[AKT])*(1+ $\mathcal{F}_{11}^{(g)}$*[AMPK])) + $b_{pep\_crbxykinase1}$ - $d_{pep\_crbxykinase1}$;
23	cit_synthase = ($\mathcal{K}_{23}^{(g)}$ *[RORa]) + $b_{cit\_synthase}$ - $d_{cit\_synthase}$;
24	actnase = ($\mathcal{K}_{24}^{(g)}$ * [HIF-1]) + $b_{actnase}$ - $d_{actnase}$;
25	isocit_deh = ($\mathcal{K}_{25}^{(g)}$ * [MYC]) + b_{isocit_deh} - d_{isocit_deh};
26	KG_deh_cmp = ($\mathcal{K}_{26}^{(g)}$ * [STAT3]*[MYC]) + $b_{KG\_deh\_cmp}$ - $d_{KG\_deh\_cmp}$;
27	succ_coa_synthase = ($\mathcal{K}_{27}^{(g)}$ * [MYC]*[E2F1]*[ERK]) + $b_{succ\_coa\_synthase}$ - $d_{succ\_coa\_synthase}$;
28	succ_deh = (($\mathcal{K}_{28}^{(g)}$ * [MYC]*[E2F1]*[p53]*[AKT])/(1 + $\mathcal{F}_{13}^{(g)}$* [LPS])) + b_{succ_deh} - d_{succ_deh};
29	frmase = ($\mathcal{K}_{29}^{(g)}$ * [MYC]*[E2F1]*[p53]*[NF-κB]) + b_{frmase} - d_{frmase};
30	mal_deh = ($\mathcal{K}_{30}^{(g)}$ * [E2F1]*[ERK]) + b_{mal_deh} - d_{mal_deh};
31	g6Pdeh = ($\mathcal{K}_{31}^{(g)}$*[mTOR])/(1 + $\mathcal{F}_{12}^{(g)}$*[p53]) + b_{g6Pdeh} - d_{g6Pdeh};
32	pglc6 = $\mathcal{K}_{32}^{(g)}$*[p53] + b_{pglc6} - d_{pglc6};
33	phglc_deh = $\mathcal{K}_{33}^{(g)}$*[p53]*[mTOR] + b_{phglc_deh} - d_{phglc_deh};
34	r5Piso_A = ($\mathcal{K}_{34}^{(g)}$ * [ERK]*[AKT]) + b_{r5Piso_A} - d_{r5Piso_A};
35	rbls_5P_3ep = $\mathcal{K}_{35}^{(g)}$*[mTOR] + $b_{rbls\_5P\_3ep}$ - $d_{rbls\_5P\_3ep}$;
36	trkl = $\mathcal{K}_{36}^{(g)}$*[mTOR] + b_{trkl} - d_{trkl};
37	trsdl1 = ($\mathcal{K}_{37}^{(g)}$ * [E2F1]*[STAT3]*[MYC]) + b_{trsdl1} - d_{trsdl1};

Table S-3 Reaction list of central carbon metabolic (CCM) pathway under consideration in the main article.

Pathway	Reaction no.	Reaction	Initial rate	Catalyzing enzyme
	1	Glusose_ext =>Glusoce	$r_0^{(m)}$	Glut1
	2	Glucose-1P =>Glucose-6P	$r_1^{(m)}$	Phosphoglucomutase_1
	3	Glucose-6P =>Glucose-1P	$r_2^{(m)}$	Phosphoglucomutase_1
	4	Glucose + ATP =>Glucose-6P+ ADP	$r_3^{(m)}$	Hexokinase
	5	Glucose-6P =>Glucose	$r_4^{(m)}$	Glucose-6-phosphatase
	6	Glucose-6P =>Fructose-6P	$r_5^{(m)}$	Phosphoglucoisomerase
	7	Fructose-6P =>Glucose-6P	$r_6^{(m)}$	Phosphoglucoisomerase
	8	Fructose-6P + ATP =>Fructose-1,6BP + ADP	$r_7^{(m)}$	Phosphofructokinase_1
	9	Fructose-1,6BP =>Fructose-6P	$r_8^{(m)}$	Fructose-1,6-bisphosphatase
G	10	Fructose-6P + ATP =>Fructose-2,6BP + ADP	$r_9^{(m)}$	Phosphofructokinase_2
L	11	Fructose-2,6BP =>Fructose-6P	$r_{10}^{(m)}$	Fructose-2,6-bisphosphatase
Y	12	Fructose-1,6BP =>DHAP + GA-3P	$r_{11}^{(m)}$	Aldolase
C	13	DHAP + GA-3P =>Fructose-1,6BP	$r_{12}^{(m)}$	Aldolase
O	14	DHAP =>GA-3P	$r_{13}^{(m)}$	Triose_phosphate_isomerase
L	15	GA-3P =>DHAP	$r_{14}^{(m)}$	Triose_phosphate_isomerase
Y	16	GA-3P + NAD =>1,3_BPG + NADH	$r_{15}^{(m)}$	Glyceraldehyde-3-phosphate_dehydrogenase
S	17	1,3_BPG + NADH =>GA-3P + NAD	$r_{16}^{(m)}$	Glyceraldehyde-3-phosphate_dehydrogenase
I	18	1,3_BPG + ADP =>3-PG + ATP	$r_{17}^{(m)}$	Phosphoglycerate_kinase
S	19	3-PG =>1,3BPG	$r_{18}^{(m)}$	Phosphoglycerate_kinase
	20	3-PG =>2-PG	$r_{19}^{(m)}$	Phosphoglycerate_mutase
	21	2-PG =>3-PG	$r_{20}^{(m)}$	Phosphoglycerate_mutase
	22	2-PG =>PEP	$r_{21}^{(m)}$	Enolase
	23	PEP=>2-PG	$r_{22}^{(m)}$	Enolase
	24	PEP + ADP =>PYR + ATP	$r_{23}^{(m)}$	Pyruvate_kinase
	25	PYR + NADH =>LACTATE + NAD	$r_{24}^{(m)}$	Lactate_dehydrogenase
	26	PYR + CoA + NAD =>Acetyle-CoA + NADH + CO2	$r_{25}^{(m)}$	Pyruvate_dehydrogenase
Fatty Acid	27	FFA + ATP + FAD + NAD =>AcetylCoA + NADH + FADH + ADP	$r_{26}^{(m)}$	Acyl-CoA_synthetase
	28	AcetylCoA + NADH + FADH + ADP =>FFA + ATP + FAD + NAD	$r_{27}^{(m)}$	Fatty_acid_synthase
	29	PYR + ATP =>OAA + ADP	$r_{28}^{(m)}$	Pyruvate_carboxylase
	30	OAA + ATP =>PEP + ADP	$r_{29}^{(m)}$	Phosphoenolpyruvate_carboxykinase_1
	31	Acetyle- CoA + OAA =>Citrate + CoA-SH	$r_{30}^{(m)}$	Citrate_synthase
	32	Citrate =>Isocitrate	$r_{31}^{(m)}$	Aconitase_1
T	33	Isocitrate =>Citrate	$r_{32}^{(m)}$	Aconitase_1
C	34	Isocitrate + NAD =>α-Ketoglutarate + NADH	$r_{33}^{(m)}$	Isocitrate_dehydrogenase
A	35	α-Ketoglutarate + NAD + CoA-SH =>Succinyl-CoA + NADH	$r_{34}^{(m)}$	α-Ketoglutarate_dehydrogenase_complex
	36	Succinyl-CoA + ADP =>Succinate + ATP	$r_{35}^{(m)}$	Succinyl_CoA_synthetase
	37	Succinate =>Succinyl-CoA	$r_{36}^{(m)}$	Succinyl_CoA_synthetase
	38	Succinate + FAD =>Fumarate + FADH	$r_{37}^{(m)}$	Succinate_dehydrogenase
	39	Fumarate =>Malate	$r_{38}^{(m)}$	Fumarase
	40	Malate + NAD =>Oxaloacetate + NADH	$r_{39}^{(m)}$	Malate_dehydrogenase
	41	Gluose-6P + NADP =>Glucono-1,5-lactone-6P + NADPH	$r_{40}^{(m)}$	Glucose-6-phosphate_dehydrogenase
	42	Glucono-1,5-lactone-6P =>Gluconate-6P	$r_{41}^{(m)}$	6-phosphogluconolactonase
	43	Gluconate-6P + NADP =>Ribulose-5P + NADPH	$r_{42}^{(m)}$	Phosphogluconate_dehydrogenase
	44	Ribulose-5P =>Ribose-5P	$r_{43}^{(m)}$	Ribose 5-phosphate_isomerise_A
	45	Ribulose-5P =>Xylulose-5P	$r_{44}^{(m)}$	Ribulose-5-phosphate-3-epimerase
P	46	Ribose-5P + Xylulose-5P =>Sedoheptolose-7P + GA-3P	$r_{45}^{(m)}$	Transketolase
P	47	Sedoheptolose-7P + GA-3P =>Erythrose-4P + Fructose-6P	$r_{46}^{(m)}$	Transaldolase_1
P	48	Erythrose-4P + Xylulose-5P =>GA-3P + Fructose-6P	$r_{47}^{(m)}$	Transketolase
	49	GA-3P + Fructose-6P =>Erythrose-4P + Xylulose-5P	$r_{48}^{(m)}$	Transketolase

Table S-4 Activation and inhibition of the reactions considered in Table S-3.

Reaction no.	Activated by	Inhibited by	Kinetic rate constants	Michaelis Menten constants	Feedback constants
1			$\mathcal{K}_1^{(m)}$	$\mathcal{K}_0^{\mathcal{M}^{(m)}}$	
2	Glucose-1,6BP		$\mathcal{K}_2^{(m)}$	$\mathcal{K}_1^{\mathcal{M}^{(m)}}$	$\mathcal{F}_1^{(m)}$
3	Glucose-1,6BP		$\mathcal{K}_2^{(m)}$	$\mathcal{K}_2^{\mathcal{M}^{(m)}}$	$\mathcal{F}_2^{(m)}$
4	Insulin, ADP	G6P, Acetyl_CoA	$\mathcal{K}_3^{(m)}$	$\mathcal{K}_3^{\mathcal{M}^{(m)}}$	$\mathcal{F}_3^{(m)}$, $\mathcal{F}_4^{(m)}$, $\mathcal{F}_5^{(m)}$, $\mathcal{F}_6^{(m)}$
5	G6P		$\mathcal{K}_4^{(m)}$	$\mathcal{K}_4^{\mathcal{M}^{(m)}}$	$\mathcal{F}_7^{(m)}$
6		Fructose-1,6BP, Gluconate-6P, Erythrose-4P	$\mathcal{K}_5^{(m)}$	$\mathcal{K}_5^{\mathcal{M}^{(m)}}$	$\mathcal{F}_8^{(m)}$, $\mathcal{F}_9^{(m)}$, $\mathcal{F}_{10}^{(m)}$
7		Fructose-1,6BP, Gluconate-6P, Erythrose-4P	$\mathcal{K}_5^{(m)}$	$\mathcal{K}_6^{\mathcal{M}^{(m)}}$	$\mathcal{F}_{11}^{(m)}$, $\mathcal{F}_{12}^{(m)}$, $\mathcal{F}_{13}^{(m)}$
8	AMP, Fructose-2,6BP	ATP, Citrate	$\mathcal{K}_6^{(m)}$	$\mathcal{K}_7^{\mathcal{M}^{(m)}}$	$\mathcal{F}_{14}^{(m)}$, $\mathcal{F}_{15}^{(m)}$, $\mathcal{F}_{16}^{(m)}$, $\mathcal{F}_{17}^{(m)}$
9		F2,6BP, AMP	$\mathcal{K}_7^{(m)}$	$\mathcal{K}_8^{\mathcal{M}^{(m)}}$	$\mathcal{F}_{18}^{(m)}$, $\mathcal{F}_{19}^{(m)}$
10	Fructose-6P	cAMP	$\mathcal{K}_8^{(m)}$	$\mathcal{K}_9^{\mathcal{M}^{(m)}}$	$\mathcal{F}_{20}^{(m)}$, $\mathcal{F}_{21}^{(m)}$
11	cAMP	Fructose-6-phosphate	$\mathcal{K}_9^{(m)}$	$\mathcal{K}_{10}^{\mathcal{M}^{(m)}}$	$\mathcal{F}_{22}^{(m)}$, $\mathcal{F}_{23}^{(m)}$
12			$\mathcal{K}_{10}^{(m)}$	$\mathcal{K}_{11}^{\mathcal{M}^{(m)}}$	
13			$\mathcal{K}_{10}^{(m)}$	$\mathcal{K}_{12}^{\mathcal{M}^{(m)}}$	
14			$\mathcal{K}_{11}^{(m)}$	$\mathcal{K}_{13}^{\mathcal{M}^{(m)}}$	
15			$\mathcal{K}_{11}^{(m)}$	$\mathcal{K}_{14}^{\mathcal{M}^{(m)}}$	
16			$\mathcal{K}_{12}^{(m)}$	$\mathcal{K}_{15}^{\mathcal{M}^{(m)}}$	
17			$\mathcal{K}_{12}^{(m)}$	$\mathcal{K}_{16}^{\mathcal{M}^{(m)}}$	
18			$\mathcal{K}_{13}^{(m)}$	$\mathcal{K}_{17}^{\mathcal{M}^{(m)}}$	
19			$\mathcal{K}_{13}^{(m)}$	$\mathcal{K}_{18}^{\mathcal{M}^{(m)}}$	
20	2,3BPG		$\mathcal{K}_{14}^{(m)}$	$\mathcal{K}_{19}^{\mathcal{M}^{(m)}}$	$\mathcal{F}_{24}^{(m)}$
21	2,3BPG		$\mathcal{K}_{14}^{(m)}$	$\mathcal{K}_{20}^{\mathcal{M}^{(m)}}$	$\mathcal{F}_{25}^{(m)}$
22			$\mathcal{K}_{15}^{(m)}$	$\mathcal{K}_{21}^{\mathcal{M}^{(m)}}$	
23			$\mathcal{K}_{15}^{(m)}$	$\mathcal{K}_{22}^{\mathcal{M}^{(m)}}$	
24	Insulin, Fructose-1,6BP	ATP, Acetyl-CoA, Alanine, Glucagon , Fatty Acid	$\mathcal{K}_{16}^{(m)}$	$\mathcal{K}_{23}^{\mathcal{M}^{(m)}}$	$\mathcal{F}_{26}^{(m)}$, $\mathcal{F}_{27}^{(m)}$, $\mathcal{F}_{28}^{(m)}$, $\mathcal{F}_{29}^{(m)}$, $\mathcal{F}_{30}^{(m)}$, $\mathcal{F}_{31}^{(m)}$, $\mathcal{F}_{32}^{(m)}$
25			$\mathcal{K}_{17}^{(m)}$	$\mathcal{K}_{24}^{\mathcal{M}^{(m)}}$	
26	Ca, Insulin		$\mathcal{K}_{18}^{(m)}$	$\mathcal{K}_{25}^{\mathcal{M}^{(m)}}$	
27			$\mathcal{K}_{19}^{(m)}$	$\mathcal{K}_{26}^{\mathcal{M}^{(m)}}$	
28			$\mathcal{K}_{20}^{(m)}$	$\mathcal{K}_{27}^{\mathcal{M}^{(m)}}$	
29	Acetyl-CoA		$\mathcal{K}_{21}^{(m)}$	$\mathcal{K}_{28}^{\mathcal{M}^{(m)}}$	$\mathcal{F}_{33}^{(m)}$
30	Glucagon	Insulin	$\mathcal{K}_{22}^{(m)}$	$\mathcal{K}_{29}^{\mathcal{M}^{(m)}}$	$\mathcal{F}_{34}^{(m)}$, $\mathcal{F}_{35}^{(m)}$
31	acetyl-CoA, Oxaloacetate	ATP, Acetyle-CoA	$\mathcal{K}_{23}^{(m)}$	$\mathcal{K}_{30}^{\mathcal{M}^{(m)}}$	$\mathcal{F}_{36}^{(m)}$, $\mathcal{F}_{37}^{(m)}$, $\mathcal{F}_{38}^{(m)}$, $\mathcal{F}_{39}^{(m)}$
32		fluoro-acetate	$\mathcal{K}_{24}^{(m)}$	$\mathcal{K}_{31}^{\mathcal{M}^{(m)}}$	$\mathcal{F}_{40}^{(m)}$
33		fluoro-acetate	$\mathcal{K}_{24}^{(m)}$	$\mathcal{K}_{32}^{\mathcal{M}^{(m)}}$	$\mathcal{F}_{41}^{(m)}$
34	isocitrate, ADP, Ca	ATP, NADH	$\mathcal{K}_{25}^{(m)}$	$\mathcal{K}_{33}^{\mathcal{M}^{(m)}}$	$\mathcal{F}_{42}^{(m)}$, $\mathcal{F}_{43}^{(m)}$, $\mathcal{F}_{44}^{(m)}$, $\mathcal{F}_{45}^{(m)}$
35	Ca	ATP, Succniyle-CoA	$\mathcal{K}_{26}^{(m)}$	$\mathcal{K}_{34}^{\mathcal{M}^{(m)}}$	$\mathcal{F}_{46}^{(m)}$, $\mathcal{F}_{47}^{(m)}$
36			$\mathcal{K}_{27}^{(m)}$	$\mathcal{K}_{35}^{\mathcal{M}^{(m)}}$	
37			$\mathcal{K}_{27}^{(m)}$	$\mathcal{K}_{36}^{\mathcal{M}^{(m)}}$	
38		Carboxin, thenoyltrifluoroacetone, malonate	$\mathcal{K}_{28}^{(m)}$	$\mathcal{K}_{37}^{\mathcal{M}^{(m)}}$	$\mathcal{F}_{48}^{(m)}$
39			$\mathcal{K}_{29}^{(m)}$	$\mathcal{K}_{38}^{\mathcal{M}^{(m)}}$	
40		Oxaloacetate	$\mathcal{K}_{30}^{(m)}$	$\mathcal{K}_{39}^{\mathcal{M}^{(m)}}$	$\mathcal{F}_{49}^{(m)}$
41		NADPH	$\mathcal{K}_{31}^{(m)}$	$\mathcal{K}_{40}^{\mathcal{M}^{(m)}}$	$\mathcal{F}_{50}^{(m)}$
42			$\mathcal{K}_{32}^{(m)}$	$\mathcal{K}_{41}^{\mathcal{M}^{(m)}}$	
43			$\mathcal{K}_{33}^{(m)}$	$\mathcal{K}_{42}^{\mathcal{M}^{(m)}}$	
44			$\mathcal{K}_{34}^{(m)}$	$\mathcal{K}_{43}^{\mathcal{M}^{(m)}}$	
45			$\mathcal{K}_{35}^{(m)}$	$\mathcal{K}_{44}^{\mathcal{M}^{(m)}}$	
46			$\mathcal{K}_{36}^{(m)}$	$\mathcal{K}_{45}^{\mathcal{M}^{(m)}}$	
47			$\mathcal{K}_{37}^{(m)}$	$\mathcal{K}_{46}^{\mathcal{M}^{(m)}}$	
48			$\mathcal{K}_{36}^{(m)}$	$\mathcal{K}_{47}^{\mathcal{M}^{(m)}}$	
49			$\mathcal{K}_{36}^{(m)}$	$\mathcal{K}_{48}^{\mathcal{M}^{(m)}}$	

Table S-5 Rate equations of the reactions considered in Table S-3.

Reaction no.	Reaction equation
1	$r_0^{(m)}$ = ($\mathcal{K}_1^{(m)}$*[glut1]*[glc_ext]) / ($\mathcal{K}_0^{\mathcal{M}^{(m)}}$ + [glc_ext]);
2	$r_1^{(m)}$ = (($\mathcal{K}_2^{(m)}$ * [pgm_1] * [glc_1P])*(1 + $\mathcal{F}_1^{(m)}$*[glc_16BP])) /($\mathcal{K}_1^{\mathcal{M}^{(m)}}$ + [glc_1P]);
3	$r_2^{(m)}$ = (($\mathcal{K}_2^{(m)}$ * [pgm_1] * [glc_6P])*(1 + $\mathcal{F}_2^{(m)}$*[glc_16BP])) /($\mathcal{K}_2^{\mathcal{M}^{(m)}}$ + [glc_6P])
4	$r_3^{(m)}$ = (($\mathcal{K}_3^{(m)}$*[hk]* [Glc]*[ATP])*(1 + $\mathcal{F}_3^{(m)}$*[Insulin])*(1 + $\mathcal{F}_4^{(m)}$*[ADP]))/((Km3 + [Glc]*[ATP])*(1 + $\mathcal{F}_5^{(m)}$*[glc_6P])*(1 + $\mathcal{F}_6^{(m)}$*[ace_coa]));
5	$r_4^{(m)}$ = ($\mathcal{K}_4^{(m)}$ *[g6Pase]*[glc_6P]*(1+ $\mathcal{F}_7^{(m)}$*[glc_6P]))/($\mathcal{K}_4^{\mathcal{M}^{(m)}}$ + [glc_6P]);
6	$r_5^{(m)}$ = ($\mathcal{K}_5^{(m)}$*[pgi]*[glc_6P])/(($\mathcal{K}_5^{\mathcal{M}^{(m)}}$ + [glc_6P])*(1 + $\mathcal{F}_8^{(m)}$*[f_16_BP])*(1 + $\mathcal{F}_9^{(m)}$*[gl_6P])*(1 + $\mathcal{F}_{10}^{(m)}$*[eryt_4P]));
7	$r_6^{(m)}$ = ($\mathcal{K}_5^{(m)}$*[pgi]*[f_6P])/(($\mathcal{K}_6^{\mathcal{M}^{(m)}}$ + [f_6P])*(1 + $\mathcal{F}_{11}^{(m)}$*[f_16_BP])*(1 + $\mathcal{F}_{12}^{(m)}$*[gl_6P])*(1 + $\mathcal{F}_{13}^{(m)}$*[eryt_4P]));
8	$r_7^{(m)}$ = (($\mathcal{K}_6^{(m)}$ *[pfk1]*[f_6P]*[ATP])*(1 + $\mathcal{F}_{14}^{(m)}$*[AMP])*(1 + $\mathcal{F}_{15}^{(m)}$*[f_26_BP]))/ (($\mathcal{K}_7^{\mathcal{M}^{(m)}}$ + [f_6P]*[ATP])*(1 + $\mathcal{F}_{16}^{(m)}$*[ATP])*(1 + $\mathcal{F}_{17}^{(m)}$*[Citrate]));
9	$r_8^{(m)}$ = ($\mathcal{K}_7^{(m)}$ *[f16Bpase]*[f_16_BP])/ (($\mathcal{K}_8^{\mathcal{M}^{(m)}}$ + [f_16_BP])*(1 + $\mathcal{F}_{18}^{(m)}$*[AMP])*(1 + $\mathcal{F}_{19}^{(m)}$*[f_26_BP]));
10	$r_9^{(m)}$ = (($\mathcal{K}_8^{(m)}$ *[pfk2]*[f_6P]*[ATP])*(1 + $\mathcal{F}_{20}^{(m)}$*[f_6P]))/ (($\mathcal{K}_9^{\mathcal{M}^{(m)}}$ + [f_6P]*[ATP])*(1 + $\mathcal{F}_{21}^{(m)}$*[cAMP]));
11	$r_{10}^{(m)}$ = (($\mathcal{K}_9^{(m)}$ *[f26Bpase]*[f_26_BP])*(1 + $\mathcal{F}_{22}^{(m)}$*[cAMP]))/(($\mathcal{K}_{10}^{\mathcal{M}^{(m)}}$ + [f_26_BP])*(1 + $\mathcal{F}_{23}^{(m)}$*[f_6P]));
12	$r_{11}^{(m)}$ = ($\mathcal{K}_{10}^{(m)}$ *[ald]*[f_16_BP])/($\mathcal{K}_{11}^{\mathcal{M}^{(m)}}$ + [f_16_BP]);
13	$r_{12}^{(m)}$ = ($\mathcal{K}_{10}^{(m)}$ *[ald]*[dhap]*[ga_3P])/($\mathcal{K}_{12}^{\mathcal{M}^{(m)}}$ + [dhap]*[ga_3P]);
14	$r_{13}^{(m)}$ = ($\mathcal{K}_{11}^{(m)}$ *[tpi]*[dhap])/($\mathcal{K}_{13}^{\mathcal{M}^{(m)}}$ + [dhap]);
15	$r_{14}^{(m)}$ = ($\mathcal{K}_{11}^{(m)}$ *[tpi]*[ga_3P])/($\mathcal{K}_{14}^{\mathcal{M}^{(m)}}$ + [ga_3P]);
16	$r_{15}^{(m)}$ = ($\mathcal{K}_{12}^{(m)}$ *[g3Pdh]*[ga_3P]*[NAD])/($\mathcal{K}_{15}^{\mathcal{M}^{(m)}}$ + [ga_3P]*[NAD]);
17	$r_{16}^{(m)}$ = ($\mathcal{K}_{12}^{(m)}$ *[g3Pdh]*[1,3_BPG]*[NADH])/ ($\mathcal{K}_{16}^{\mathcal{M}^{(m)}}$ + [1,3_BPG]*[NADH]);
18	$r_{17}^{(m)}$ = ($\mathcal{K}_{13}^{(m)}$ *[pglck]*[1,3_BPG]*[ADP])/($\mathcal{K}_{17}^{\mathcal{M}^{(m)}}$ + [1,3_BPG]*[ADP]);
19	$r_{18}^{(m)}$ = ($\mathcal{K}_{13}^{(m)}$ *[pglck]*[PG_3])/($\mathcal{K}_{18}^{\mathcal{M}^{(m)}}$ + [PG_3]);
20	$r_{19}^{(m)}$ = (($\mathcal{K}_{14}^{(m)}$ *[pglcm]*[PG_3])*(1 +$\mathcal{F}_{24}^{(m)}$*[BPG_23]))/($\mathcal{K}_{19}^{\mathcal{M}^{(m)}}$ + [PG_3]);
21	$r_{20}^{(m)}$ = (($\mathcal{K}_{14}^{(m)}$ *[pglcm]*[PG_2])*(1 +$\mathcal{F}_{25}^{(m)}$*[BPG_23]))/($\mathcal{K}_{20}^{\mathcal{M}^{(m)}}$ + [PG_2]);
22	$r_{21}^{(m)}$ = ($\mathcal{K}_{15}^{(m)}$ *[enl]*[PG_2])/($\mathcal{K}_{21}^{\mathcal{M}^{(m)}}$ + [PG_2]);
23	$r_{22}^{(m)}$ = ($\mathcal{K}_{15}^{(m)}$ *[enl]*[PEP])/($\mathcal{K}_{22}^{\mathcal{M}^{(m)}}$ + [PEP]);
24	$r_{23}^{(m)}$ = (($\mathcal{K}_{16}^{(m)}$ *[pk]*[PEP]*[ADP])*(1 + $\mathcal{F}_{26}^{(m)}$*[Insulin])*(1 + $\mathcal{F}_{27}^{(m)}$*[f_16_BP]))/ (($\mathcal{K}_{23}^{\mathcal{M}^{(m)}}$ + [PEP]*[ADP])*(1 + $\mathcal{F}_{28}^{(m)}$*[ATP])*(1 + $\mathcal{F}_{29}^{(m)}$*[Ace_coa])*(1 + $\mathcal{F}_{30}^{(m)}$*[Alanine])* (1 + $\mathcal{F}_{31}^{(m)}$*[Glucagon])*(1 + $\mathcal{F}_{32}^{(m)}$*[FFA]));
25	$r_{24}^{(m)}$ = ($\mathcal{K}_{17}^{(m)}$ *[ldh]*[PYR]*[NADH])/($\mathcal{K}_{24}^{\mathcal{M}^{(m)}}$ + [PYR]*[NADH]);
26	$r_{25}^{(m)}$ = ($\mathcal{K}_{18}^{(m)}$ *[pyrd]*[PYR]*[COA]*[NAD]*(1+$\mathcal{F}_{51}^{(m)}$*[Ca]))/($\mathcal{K}_{25}^{\mathcal{M}^{(m)}}$ + [PYR] *[COA]*[NAD]);
27	$r_{26}^{(m)}$ = ($\mathcal{K}_{19}^{(m)}$ *[Acyl_coa_syn]*[FFA]*[ATP]*[FAD]*[NAD]) / ($\mathcal{K}_{26}^{\mathcal{M}^{(m)}}$ + [FFA]*[ATP]*[FAD]*[NAD]);
28	$r_{27}^{(m)}$ = ($\mathcal{K}_{20}^{(m)}$ *[fa_synthase]*[Acetyl-CoA]*[NADH]*[FADH]*[ADP]) / ($\mathcal{K}_{27}^{\mathcal{M}^{(m)}}$ + [Acetyl-CoA]*[NADH]*[FADH]*[ADP]);
29	$r_{28}^{(m)}$ = (($\mathcal{K}_{21}^{(m)}$ *[pyr_crbxylase]*[PYR]*[ATP])*(1 + $\mathcal{F}_{33}^{(m)}$*[Ace_coa]))/($\mathcal{K}_{28}^{\mathcal{M}^{(m)}}$ + [PYR]*[ATP]);
30	$r_{29}^{(m)}$ = (($\mathcal{K}_{22}^{(m)}$ *[pep_crbxykinase1]*[OAA]*[ATP])*(1 + $\mathcal{F}_{34}^{(m)}$*[Glucagon]))/(($\mathcal{K}_{29}^{\mathcal{M}^{(m)}}$ + [OAA]*[ATP])*(1 + $\mathcal{F}_{35}^{(m)}$*[Insulin]));
31	$r_{30}^{(m)}$ = ($\mathcal{K}_{23}^{(m)}$ *[cit_synthase]*[Ace_coa]*[OAA]*(1 + $\mathcal{F}_{36}^{(m)}$*[Ace_coa])*(1 + $\mathcal{F}_{37}^{(m)}$*[OAA]))/(($\mathcal{K}_{30}^{\mathcal{M}^{(m)}}$ + [Ace_coa]*[OAA])*(1 + $\mathcal{F}_{38}^{(m)}$*[ATP])*(1 + $\mathcal{F}_{39}^{(m)}$*[Ace_coa]));
32	$r_{31}^{(m)}$ = ($\mathcal{K}_{24}^{(m)}$ *[actnase]*[citrate])/(($\mathcal{K}_{31}^{\mathcal{M}^{(m)}}$ + [citrate])* (1 + $\mathcal{F}_{40}^{(m)}$*[fluoro_acetate]));
33	$r_{32}^{(m)}$ = ($\mathcal{K}_{24}^{(m)}$ *[actnase]*[Isocitrate])/(($\mathcal{K}_{32}^{\mathcal{M}^{(m)}}$ + [Isocitrate])* (1 + $\mathcal{F}_{41}^{(m)}$*[fluoro_acetate]));
34	$r_{33}^{(m)}$ = ($\mathcal{K}_{25}^{(m)}$ *[isocit_deh]*[isocitrate]*[NAD]*(1+$\mathcal{F}_{51}^{(m)}$*[Ca])*(1 + $\mathcal{F}_{42}^{(m)}$*[isocitrate])*(1 + $\mathcal{F}_{43}^{(m)}$*[ADP]))/ (($\mathcal{K}_{33}^{\mathcal{M}^{(m)}}$ + [isocitrate]*[NAD])*(1 + $\mathcal{F}_{44}^{(m)}$*[ATP])*(1 + $\mathcal{F}_{45}^{(m)}$*[NADH]));
35	$r_{34}^{(m)}$ = ($\mathcal{K}_{26}^{(m)}$ *[KG_deh_cmp]*[alpha_KG]*[NAD]*[COA_SH]*(1+$\mathcal{F}_{51}^{(m)}$*[Ca]))/ (($\mathcal{K}_{34}^{\mathcal{M}^{(m)}}$ + [alpha_KG]*[NAD]*[COA_SH])*(1 + $\mathcal{F}_{46}^{(m)}$*[ATP])*(1 + $\mathcal{F}_{47}^{(m)}$*[Succnyle-CoA]));
36	$r_{35}^{(m)}$ = ($\mathcal{K}_{27}^{(m)}$ *[succ_coa_synthase]*[Succ_coa]*[ADP])/($\mathcal{K}_{35}^{\mathcal{M}^{(m)}}$ + [Succ_coa]*[ADP]);
37	$r_{36}^{(m)}$ = ($\mathcal{K}_{27}^{(m)}$ *[succ_coa_synthase]*[Succinate])/($\mathcal{K}_{36}^{\mathcal{M}^{(m)}}$ + [Succinate]);
38	$r_{37}^{(m)}$ = ($\mathcal{K}_{28}^{(m)}$ *[succ_deh]*[succinate]*[FAD])/ (($\mathcal{K}_{37}^{\mathcal{M}^{(m)}}$ + [succinate]*[FAD])*(1 + $\mathcal{F}_{48}^{(m)}$*[Carboxin]));
39	$r_{38}^{(m)}$ = ($\mathcal{K}_{29}^{(m)}$ *[frmase]*[Furmarate])/($\mathcal{K}_{38}^{\mathcal{M}^{(m)}}$ + [Furmarate]);
40	$r_{39}^{(m)}$ = ($\mathcal{K}_{30}^{(m)}$ *[mal_deh]*[Malate]*[NAD])/(($\mathcal{K}_{39}^{\mathcal{M}^{(m)}}$ + [Malate]*[NAD])*(1 + $\mathcal{F}_{49}^{(m)}$*[OAA]));
41	$r_{40}^{(m)}$ = ($\mathcal{K}_{31}^{(m)}$ *[g6P_deh]*[glc_6P]*[NADP])/(($\mathcal{K}_{40}^{\mathcal{M}^{(m)}}$ + [glc_6P]*[NADP])*(1 + $\mathcal{F}_{50}^{(m)}$*[NADPH]));
42	$r_{41}^{(m)}$ = ($\mathcal{K}_{32}^{(m)}$ *[pglc6]*[gl_15_l6P])/($\mathcal{K}_{41}^{\mathcal{M}^{(m)}}$ + [gl_15_l6P]);
43	$r_{42}^{(m)}$ = ($\mathcal{K}_{33}^{(m)}$ *[phglc_deh]*[gl_6P]*[NADP])/($\mathcal{K}_{42}^{\mathcal{M}^{(m)}}$ + [gl_6P]*[NADP]);
44	$r_{43}^{(m)}$ = ($\mathcal{K}_{34}^{(m)}$ *[r5Piso_A]*[rbls_5P])/($\mathcal{K}_{43}^{\mathcal{M}^{(m)}}$ + [rbls_5P]);
45	$r_{44}^{(m)}$ = ($\mathcal{K}_{35}^{(m)}$ *[rbls_5P_3ep]*[rbls_5P])/($\mathcal{K}_{44}^{\mathcal{M}^{(m)}}$ + [rbls_5P]);
46	$r_{45}^{(m)}$ = ($\mathcal{K}_{36}^{(m)}$ *[trkl]*[Rbs_5P]*[Xyl_5P])/($\mathcal{K}_{45}^{\mathcal{M}^{(m)}}$ + [Rbs_5P]*[Xyl_5P]);
47	$r_{46}^{(m)}$ = (($\mathcal{K}_{37}^{(m)}$ *[trsdl_1]*[sdhl_7P]*[GA-3P]))/($\mathcal{K}_{46}^{\mathcal{M}^{(m)}}$ + [sdhl_7P]*[GA-3P]));
48	$r_{47}^{(m)}$ = ($\mathcal{K}_{36}^{(m)}$ *[trkl]*[eryt_4P]*[xyl_5P])/($\mathcal{K}_{47}^{\mathcal{M}^{(m)}}$ + [eryt_4P]*[xyl_5P]);
49	$r_{48}^{(m)}$ = ($\mathcal{K}_{36}^{(m)}$ *[trkl]*[GA-3P]*[f_6P])/($\mathcal{K}_{48}^{\mathcal{M}^{(m)}}$ + [GA-3P]*[f_6P]);

Table S-6 Stoichiometric matrix of the reactions considered in Table S-3.

	$r_0^{(m)}$	$r_1^{(m)}$	$r_2^{(m)}$	$r_3^{(m)}$	$r_4^{(m)}$	$r_5^{(m)}$	$r_6^{(m)}$	$r_7^{(m)}$	$r_8^{(m)}$	$r_9^{(m)}$	$r_{10}^{(m)}$	$r_{11}^{(m)}$	$r_{12}^{(m)}$	$r_{13}^{(m)}$	$r_{14}^{(m)}$	$r_{15}^{(m)}$	$r_{16}^{(m)}$	$r_{17}^{(m)}$	$r_{18}^{(m)}$	$r_{19}^{(m)}$	$r_{20}^{(m)}$	$r_{21}^{(m)}$	$r_{22}^{(m)}$	$r_{23}^{(m)}$	$r_{24}^{(m)}$
Glucose-1P	0	-1	1	0	0	0	0	0	0	0	0	0	0	0	0	0	0	0	0	0	0	0	0	0	0
Glucose-6P	0	1	-1	1	-1	-1	1	0	0	0	0	0	0	0	0	0	0	0	0	0	0	0	0	0	0
Glucose	1	0	0	-1	1	0	0	0	0	0	0	0	0	0	0	0	0	0	0	0	0	0	0	0	0
Fructose-6P	0	0	0	0	0	1	-1	-1	1	-1	1	0	0	0	0	0	0	0	0	0	0	0	0	0	0
Fructose-1,6BP	0	0	0	0	0	0	0	1	-1	0	0	-1	1	0	0	0	0	0	0	0	0	0	0	0	0
Fructose-2,6BP	0	0	0	0	0	0	0	0	0	1	-1	0	0	0	0	0	0	0	0	0	0	0	0	0	0
DHAP	0	0	0	0	0	0	0	0	0	0	0	1	-1	-1	1	0	0	0	0	0	0	0	0	0	0
GA-3P	0	0	0	0	0	0	0	0	0	0	0	1	-1	1	-1	-1	1	0	0	0	0	0	0	0	0
1,3-BPG	0	0	0	0	0	0	0	0	0	0	0	0	0	0	0	1	-1	-1	1	0	0	0	0	0	0
3-PG	0	0	0	0	0	0	0	0	0	0	0	0	0	0	0	0	0	1	-1	-1	1	0	0	0	0
2-PG	0	0	0	0	0	0	0	0	0	0	0	0	0	0	0	0	0	0	0	1	-1	-1	1	0	0
PEP	0	0	0	0	0	0	0	0	0	0	0	0	0	0	0	0	0	0	0	0	0	1	-1	-1	0
PYR	0	0	0	0	0	0	0	0	0	0	0	0	0	0	0	0	0	0	0	0	0	0	0	1	-1
LACTATE	0	0	0	0	0	0	0	0	0	0	0	0	0	0	0	0	0	0	0	0	0	0	0	0	1
Acetyle-CoA	0	0	0	0	0	0	0	0	0	0	0	0	0	0	0	0	0	0	0	0	0	0	0	0	0
OAA	0	0	0	0	0	0	0	0	0	0	0	0	0	0	0	0	0	0	0	0	0	0	0	0	0
Citrate	0	0	0	0	0	0	0	0	0	0	0	0	0	0	0	0	0	0	0	0	0	0	0	0	0
Isocitrate	0	0	0	0	0	0	0	0	0	0	0	0	0	0	0	0	0	0	0	0	0	0	0	0	0
α-Ketoglutarate	0	0	0	0	0	0	0	0	0	0	0	0	0	0	0	0	0	0	0	0	0	0	0	0	0
Succinyl-CoA	0	0	0	0	0	0	0	0	0	0	0	0	0	0	0	0	0	0	0	0	0	0	0	0	0
Succinate	0	0	0	0	0	0	0	0	0	0	0	0	0	0	0	0	0	0	0	0	0	0	0	0	0
Fumarate	0	0	0	0	0	0	0	0	0	0	0	0	0	0	0	0	0	0	0	0	0	0	0	0	0
Malate	0	0	0	0	0	0	0	0	0	0	0	0	0	0	0	0	0	0	0	0	0	0	0	0	0
Glucono-1,5-lactone-6P	0	0	0	0	0	0	0	0	0	0	0	0	0	0	0	0	0	0	0	0	0	0	0	0	0
Gluconate-6P	0	0	0	0	0	0	0	0	0	0	0	0	0	0	0	0	0	0	0	0	0	0	0	0	0
Ribulose-5P	0	0	0	0	0	0	0	0	0	0	0	0	0	0	0	0	0	0	0	0	0	0	0	0	0
Ribose-5P	0	0	0	0	0	0	0	0	0	0	0	0	0	0	0	0	0	0	0	0	0	0	0	0	0
Xylulose-5P	0	0	0	0	0	0	0	0	0	0	0	0	0	0	0	0	0	0	0	0	0	0	0	0	0
Sedoheptolose-7P	0	0	0	0	0	0	0	0	0	0	0	0	0	0	0	0	0	0	0	0	0	0	0	0	0
Erythrose-4P	0	0	0	0	0	0	0	0	0	0	0	0	0	0	0	0	0	0	0	0	0	0	0	0	0
ATP	0	0	0	-1	0	0	0	-1	0	-1	0	0	0	0	0	0	0	1	0	0	0	0	0	1	0
ADP	0	0	0	1	0	0	0	1	0	1	0	0	0	0	0	0	0	-1	0	0	0	0	0	-1	0
NADH	0	0	0	0	0	0	0	0	0	0	0	0	0	0	0	1	-1	0	0	0	0	0	0	0	-1
NAD	0	0	0	0	0	0	0	0	0	0	0	0	0	0	0	-1	1	0	0	0	0	0	0	0	1
CoA	0	0	0	0	0	0	0	0	0	0	0	0	0	0	0	0	0	0	0	0	0	0	0	0	0
CO2	0	0	0	0	0	0	0	0	0	0	0	0	0	0	0	0	0	0	0	0	0	0	0	0	0
FFA	0	0	0	0	0	0	0	0	0	0	0	0	0	0	0	0	0	0	0	0	0	0	0	0	0
FAD	0	0	0	0	0	0	0	0	0	0	0	0	0	0	0	0	0	0	0	0	0	0	0	0	0
FADH	0	0	0	0	0	0	0	0	0	0	0	0	0	0	0	0	0	0	0	0	0	0	0	0	0
CoA-SH	0	0	0	0	0	0	0	0	0	0	0	0	0	0	0	0	0	0	0	0	0	0	0	0	0
NADPH	0	0	0	0	0	0	0	0	0	0	0	0	0	0	0	0	0	0	0	0	0	0	0	0	0

	$r_{25}^{(m)}$	$r_{26}^{(m)}$	$r_{27}^{(m)}$	$r_{28}^{(m)}$	$r_{29}^{(m)}$	$r_{30}^{(m)}$	$r_{31}^{(m)}$	$r_{32}^{(m)}$	$r_{33}^{(m)}$	$r_{34}^{(m)}$	$r_{35}^{(m)}$	$r_{36}^{(m)}$	$r_{37}^{(m)}$	$r_{38}^{(m)}$	$r_{39}^{(m)}$	$r_{40}^{(m)}$	$r_{41}^{(m)}$	$r_{42}^{(m)}$	$r_{43}^{(m)}$	$r_{44}^{(m)}$	$r_{45}^{(m)}$	$r_{46}^{(m)}$	$r_{47}^{(m)}$	$r_{48}^{(m)}$
Glucose-1P	0	0	0	0	0	0	0	0	0	0	0	0	0	0	0	0	0	0	0	0	0	0	0	0
Glucose-6P	0	0	0	0	0	0	0	0	0	0	0	0	0	0	0	-1	0	0	0	0	0	0	0	0
Glucose	0	0	0	0	0	0	0	0	0	0	0	0	0	0	0	0	0	0	0	0	0	0	0	0
Fructose-6P	0	0	0	0	0	0	0	0	0	0	0	0	0	0	0	0	0	0	0	0	0	1	1	-1
Fructose-1,6BP	0	0	0	0	0	0	0	0	0	0	0	0	0	0	0	0	0	0	0	0	0	0	0	0
Fructose-2,6BP	0	0	0	0	0	0	0	0	0	0	0	0	0	0	0	0	0	0	0	0	0	0	0	0
DHAP	0	0	0	0	0	0	0	0	0	0	0	0	0	0	0	0	0	0	0	0	0	0	0	0
GA-3P	0	0	0	0	0	0	0	0	0	0	0	0	0	0	0	0	0	0	0	0	1	-1	-1	-1
1,3-BPG	0	0	0	0	0	0	0	0	0	0	0	0	0	0	0	0	0	0	0	0	0	0	0	0
3-PG	0	0	0	0	0	0	0	0	0	0	0	0	0	0	0	0	0	0	0	0	0	0	0	0
2-PG	0	0	0	0	0	0	0	0	0	0	0	0	0	0	0	0	0	0	0	0	0	0	0	0
PEP	0	0	0	0	1	0	0	0	0	0	0	0	0	0	0	0	0	0	0	0	0	0	0	0
PYR	-1	0	0	-1	0	0	0	0	0	0	0	0	0	0	0	0	0	0	0	0	0	0	0	0
LACTATE	0	0	0	0	0	0	0	0	0	0	0	0	0	0	0	0	0	0	0	0	0	0	0	0
Acetyle-CoA	1	1	-1	0	0	-1	0	0	0	0	0	0	0	0	0	0	0	0	0	0	0	0	0	0
OAA	0	0	0	1	-1	-1	0	0	0	0	0	0	0	0	1	0	0	0	0	0	0	0	0	0
Citrate	0	0	0	0	0	1	-1	1	0	0	0	0	0	0	0	0	0	0	0	0	0	0	0	0
Isocitrate	0	0	0	0	0	0	1	-1	-1	0	0	0	0	0	0	0	0	0	0	0	0	0	0	0
α-Ketoglutarate	0	0	0	0	0	0	0	0	1	-1	0	0	0	0	0	0	0	0	0	0	0	0	0	0
Succinyl-CoA	0	0	0	0	0	0	0	0	0	1	-1	1	0	0	0	0	0	0	0	0	0	0	0	0
Succinate	0	0	0	0	0	0	0	0	0	0	1	-1	-1	0	0	0	0	0	0	0	0	0	0	0
Fumarate	0	0	0	0	0	0	0	0	0	0	0	0	1	-1	0	0	0	0	0	0	0	0	0	0
Malate	0	0	0	0	0	0	0	0	0	0	0	0	0	1	-1	0	0	0	0	0	0	0	0	0
Glucono-1,5-lactone-6P	0	0	0	0	0	0	0	0	0	0	0	0	0	0	0	1	-1	0	0	0	0	0	0	0
Gluconate-6P	0	0	0	0	0	0	0	0	0	0	0	0	0	0	0	0	1	-1	0	0	0	0	0	0
Ribulose-5P	0	0	0	0	0	0	0	0	0	0	0	0	0	0	0	0	0	1	-1	-1	0	0	0	0
Ribose-5P	0	0	0	0	0	0	0	0	0	0	0	0	0	0	0	0	0	0	1	0	-1	0	0	0
Xylulose-5P	0	0	0	0	0	0	0	0	0	0	0	0	0	0	0	0	0	0	0	1	-1	0	-1	1
Sedoheptolose-7P	0	0	0	0	0	0	0	0	0	0	0	0	0	0	0	0	0	0	0	0	1	-1	0	0
Erythrose-4P	0	0	0	0	0	0	0	0	0	0	0	0	0	0	0	0	0	0	0	0	0	1	-1	1
ATP	0	-1	1	-1	-1	0	0	0	0	0	1	0	0	0	0	0	0	0	0	0	0	0	0	0
ADP	0	1	-1	1	1	0	0	0	0	0	-1	0	0	0	0	0	0	0	0	0	0	0	0	0
NADH	1	1	-1	0	0	0	0	0	1	1	0	0	0	0	1	0	0	0	0	0	0	0	0	0
NAD	-1	-1	1	0	0	0	0	0	-1	-1	0	0	0	0	-1	0	0	0	0	0	0	0	0	0
CoA	-1	0	0	0	0	0	0	0	0	0	0	0	0	0	0	0	0	0	0	0	0	0	0	0
CO2	1	0	0	0	0	0	0	0	0	0	0	0	0	0	0	0	0	0	0	0	0	0	0	0
FFA	0	-1	1	0	0	0	0	0	0	0	0	0	0	0	0	0	0	0	0	0	0	0	0	0
FAD	0	-1	1	0	0	0	0	0	0	0	0	0	-1	0	0	0	0	0	0	0	0	0	0	0
FADH	0	1	-1	0	0	0	0	0	0	0	0	0	1	0	0	0	0	0	0	0	0	0	0	0
CoA-SH	0	0	0	0	0	1	0	0	0	-1	0	0	0	0	0	0	0	0	0	0	0	0	0	0
NADPH	0	0	0	0	0	0	0	0	0	0	0	0	0	0	0	1	0	1	0	0	0	0	0	0

Table S-7 Details of signaling proteins/molecules and activating interaction equations under consideration in the main article.

Signaling proteins	Activated by	Inhibited by	Feedback constants	Binding constants	Activating interaction equations
STAT3	IL6, GF			$\mathcal{K}_1^{(s)}, \mathcal{K}_{31}^{(s)}$	$r_1^{(s)} = (\mathcal{K}_1^{(s)}*[IL6]), r_{31}^{(s)} = (\mathcal{K}_{31}^{(s)}*[GF])$
NF-κB	LPS, IFN-y, TNF, IL1, TRAF6, PI3K, AKT			$\mathcal{K}_{32}^{(s)}, \mathcal{K}_{33}^{(s)}, \mathcal{K}_{34}^{(s)}, \mathcal{K}_{35}^{(s)}, \mathcal{K}_2^{(s)}$	$r_{32}^{(s)} = (\mathcal{K}_{32}^{(s)}*[LPS]), r_{33}^{(s)} = (\mathcal{K}_{33}^{(s)}*[IFN\text{-}y]), r_{34}^{(s)} = (\mathcal{K}_{34}^{(s)}*[TNF]), r_{35}^{(s)} = (\mathcal{K}_{35}^{(s)}*[IL1]), r_2^{(s)} = (\mathcal{K}_2^{(s)}*[TRAF6]*[PI3K]*[AKT])$
ERK	GF, Raf-1, Ras	PTP, MKP	$\mathcal{F}_1^{(s)}, \mathcal{F}_2^{(s)}$	$\mathcal{K}_{36}^{(s)}, \mathcal{K}_3^{(s)}$	$r_{36}^{(s)} = (\mathcal{K}_{36}^{(s)}*[GF]), r_3^{(s)} = (\mathcal{K}_3^{(s)}*[Raf\text{-}1]*[Ras])/((1 + \mathcal{F}_1^{(s)}*[PTP])*(1 + \mathcal{F}_2^{(s)}*[MKP]))$
P13K	GF, GPCR, Ras	PTEN	$\mathcal{F}_3^{(s)}$	$\mathcal{K}_{37}^{(s)}, \mathcal{K}_{38}^{(s)}, \mathcal{K}_{52}^{(s)}, \mathcal{K}_4^{(s)}$	$r_{37}^{(s)} = (\mathcal{K}_{37}^{(s)}*[GF]), r_{38}^{(s)} = (\mathcal{K}_{38}^{(s)}*[GPCR]), r_{52}^{(s)} = (\mathcal{K}_{52}^{(s)}*[GF]), r_4^{(s)} = (\mathcal{K}_4^{(s)}*[Ras]) / (1 + \mathcal{F}_3^{(s)}*[PTEN])$
AKT	P13K, MTORC2	PTEN	$\mathcal{F}_4^{(s)}$	$\mathcal{K}_5^{(s)}$	$r_5^{(s)} = (\mathcal{K}_5^{(s)}*[P13K]*[MTORC2])/(1 + \mathcal{F}_4^{(s)}*[PTEN])$
mTOR	AKT, ROS, Rheb, P13K, GPCR	LKB1	$\mathcal{F}_5^{(s)}$	$\mathcal{K}_6^{(s)}, \mathcal{K}_{53}^{(s)}$	$r_6^{(s)} = (\mathcal{K}_6^{(s)}*[AKT]*[ROS]*[Rheb]*[P13K])/(1 + \mathcal{F}_5^{(s)}*[LKB1]), r_{53}^{(s)} = (\mathcal{K}_{53}^{(s)}*[GPCR]$
MNK	ERK			$\mathcal{K}_7^{(s)}$	$r_7^{(s)} = (\mathcal{K}_7^{(s)}*[ERK]$
HIF-1	ERK, mTOR,elf4E, STAT3, NF-κB	VHL, PHD	$\mathcal{F}_6^{(s)}, \mathcal{F}_7^{(s)}$	$\mathcal{K}_8^{(s)}$	$r_8^{(s)} = (\mathcal{K}_8^{(s)}*[ERK]*[mTOR]*[elf4E]*[STAT3]*[NF\text{-}\kappa B])/((1 + \mathcal{F}_6^{(s)}*[VHL])*(1 + \mathcal{F}_7^{(s)}*[PHD]))$
elf4E	MNK	ERK, mTOR	$\mathcal{F}_8^{(s)}, \mathcal{F}_9^{(s)}$	$\mathcal{K}_9^{(s)}$	$r_9^{(s)} = (\mathcal{K}_9^{(s)}*[MNK])/((1 + \mathcal{F}_8^{(s)}*[ERK])*(1 + \mathcal{F}_9^{(s)}*[mTOR]))$
Ras	GF, cAMP	GNP	$\mathcal{F}_{10}^{(s)}$	$\mathcal{K}_{39}^{(s)}, \mathcal{K}_{43}^{(s)}, \mathcal{K}_{10}^{(s)}$	$r_{39}^{(s)} = (\mathcal{K}_{39}^{(s)}*[GF]), r_{43}^{(s)} = (\mathcal{K}_{43}^{(s)}*[cAMP]), r_{10}^{(s)} = (\mathcal{K}_{10}^{(s)}/(1 + \mathcal{F}_{10}^{(s)}*[GNP]))$
Raf-1	Ras	AKT	$\mathcal{F}_{13}^{(s)}$	$\mathcal{K}_{11}^{(s)}$	$r_{11}^{(s)} = (\mathcal{K}_{11}^{(s)}*[Ras]) / (1 + \mathcal{F}_{13}^{(s)}*[AKT])$
AMPK	AMP, LKB1, ATP			$\mathcal{K}_{12}^{(s)}, \mathcal{K}_{44}^{(s)}, \mathcal{K}_{45}^{(s)}$	$r_{12}^{(s)} = (\mathcal{K}_{12}^{(s)}*[ATP]), r_{44}^{(s)} = (\mathcal{K}_{44}^{(s)}*[AMP]), r_{45}^{(s)} = (\mathcal{K}_{45}^{(s)}*[LKB1])$
Rheb	Wnt, TNF	AMPK, ERK	$\mathcal{F}_{14}^{(s)}, \mathcal{F}_{15}^{(s)}$	$\mathcal{K}_{46}^{(s)}, \mathcal{K}_{47}^{(s)}, \mathcal{K}_{13}^{(s)}$	$r_{47}^{(s)} = (\mathcal{K}_{47}^{(s)}*[TNF]), r_{46}^{(s)} = (\mathcal{K}_{46}^{(s)}*[Wnt]), r_{13}^{(s)} = \mathcal{K}_{13}^{(s)}/((1 + \mathcal{F}_{14}^{(s)}*[AMPK])*(1 + \mathcal{F}_{15}^{(s)}*[ERK]))$
PDK1	P13K, HIF-1	PTEN	$\mathcal{F}_{16}^{(s)}$	$\mathcal{K}_{14}^{(s)}$	$r_{14}^{(s)} = (\mathcal{K}_{14}^{(s)}*[P13K]*[HIF\text{-}1])/(1 + \mathcal{F}_{16}^{(s)}*[PTEN])$
PKCs	PDK1			$\mathcal{K}_{15}^{(s)}$	$r_{15}^{(s)} = (\mathcal{K}_{15}^{(s)}*[PDK1])$
TRAF6	TNF, IL1			$\mathcal{K}_{48}^{(s)}, \mathcal{K}_{49}^{(s)}$	$r_{48}^{(s)} = (\mathcal{K}_{48}^{(s)}*[TNF]), r_{49}^{(s)} = (\mathcal{K}_{49}^{(s)}*[IL1])$
BCL-X	NF-κB	P13K, AKT	$\mathcal{F}_{17}^{(s)}, \mathcal{F}_{18}^{(s)}$	$\mathcal{K}_{17}^{(s)}$	$r_{17}^{(s)} = (\mathcal{K}_{17}^{(s)}*[NF\text{-}\kappa B])/((1 + \mathcal{F}_{17}^{(s)}*[P13K])*(1 + \mathcal{F}_{18}^{(s)}*[AKT]))$
FASL	P13K	AKT	$\mathcal{F}_{19}^{(s)}$	$\mathcal{K}_{18}^{(s)}$	$r_{18}^{(s)} = (\mathcal{K}_{18}^{(s)}*[P13K])/(1 + \mathcal{F}_{19}^{(s)}*[AKT])$
MDM2	p53, MDMX, AKT	MYC, Ras, E2F1	$\mathcal{F}_{20}^{(s)}, \mathcal{F}_{21}^{(s)}, \mathcal{F}_{22}^{(s)}$	$\mathcal{K}_{19}^{(s)}$	$r_{19}^{(s)} = (\mathcal{K}_{19}^{(s)}*[p53]*[MDMX]*[AKT])/((1 + \mathcal{F}_{20}^{(s)}*[MYC])*(1 + \mathcal{F}_{21}^{(s)}*[Ras])*(1 + \mathcal{F}_{22}^{(s)}*[E2F1]))$
p53	ATMATR,AMPK, HIF-1	MDM2, GPCR	$\mathcal{F}_{23}^{(s)}, \mathcal{F}_{11}^{(s)}$	$\mathcal{K}_{20}^{(s)}, \mathcal{K}_{50}^{(s)}$	$r_{20}^{(s)} = (\mathcal{K}_{20}^{(s)}*[AMPK]*[HIF\text{-}1])/((1 + \mathcal{F}_{23}^{(s)}*[MDM2])*(1 + \mathcal{F}_{11}^{(s)}*[GPCR])), r_{50}^{(s)} = (\mathcal{K}_{50}^{(s)}*[ATMATR])$
MDMX		MDM2	$\mathcal{F}_{24}^{(s)}$	$\mathcal{K}_{21}^{(s)}$	$r_{21}^{(s)} = \mathcal{K}_{21}^{(s)}/(1 + \mathcal{F}_{24}^{(s)}*[MDM2])$
PTEN	p53			$\mathcal{K}_{22}^{(s)}$	$r_{22}^{(s)} = (\mathcal{K}_{22}^{(s)}*[p53])$
VHL		Succinate, Fumarate, ROS	$\mathcal{F}_{25}^{(s)}, \mathcal{F}_{26}^{(s)}, \mathcal{F}_{27}^{(s)}$	$\mathcal{K}_{23}^{(s)}$	$r_{23}^{(s)} = \mathcal{K}_{23}^{(s)}/((1 + \mathcal{F}_{25}^{(s)}*[Succinate])*(1 + \mathcal{F}_{26}^{(s)}*[Farmarate])*(1 + \mathcal{F}_{27}^{(s)}*[ROS]))$
PHD		ROS, Succinate, Fumarate	$\mathcal{F}_{28}^{(s)}, \mathcal{F}_{31}^{(s)}, \mathcal{F}_{32}^{(s)}$	$\mathcal{K}_{24}^{(s)}, \mathcal{K}_{30}^{(s)}$	$r_{24}^{(s)} = \mathcal{K}_{24}^{(s)}/(1 + \mathcal{F}_{28}^{(s)}*[ROS]), r_{30}^{(s)} = \mathcal{K}_{30}^{(s)}/((1 + \mathcal{F}_{31}^{(s)} * [Succinate])*(1 + \mathcal{F}_{32}^{(s)}*[Fumarate]))$
TIGAR	p53			$\mathcal{K}_{25}^{(s)}$	$r_{25}^{(s)} = (\mathcal{K}_{25}^{(s)}*[p53])$
MTORC2	ATP, AMP	MTORC1	$\mathcal{F}_{29}^{(s)}$	$\mathcal{K}_{16}^{(s)}, \mathcal{K}_{26}^{(s)}$	$r_{16}^{(s)} = (\mathcal{K}_{16}^{(s)}*[AMP]), r_{26}^{(s)} = (\mathcal{K}_{26}^{(s)}*[ATP])/(1 + \mathcal{F}_{29}^{(s)}*[MTORC1])$
MTORC1	AKT	MTORC2	$\mathcal{F}_{30}^{(s)}$	$\mathcal{K}_{27}^{(s)}$	$r_{27}^{(s)} = (\mathcal{K}_{27}^{(s)}*[AKT])/(1 + \mathcal{F}_{30}^{(s)}*[MTORC2])$
ROS	MTORC1, ATP			$\mathcal{K}_{28}^{(s)}$	$r_{28}^{(s)} = (\mathcal{K}_{28}^{(s)}*[MTORC1]*[ATP])$
MYC	ERK, IFN-y			$\mathcal{K}_{29}^{(s)}, \mathcal{K}_{54}^{(s)}$	$r_{29}^{(s)} = (\mathcal{K}_{29}^{(s)}*[ERK]), r_{54}^{(s)} = (\mathcal{K}_{54}^{(s)}*[IFN\text{-}y])$

Table S-8 Interaction matrix containing the information about interactions among the signaling proteins/molecules considered in Table S-7.

	$r_1^{(s)}$	$r_2^{(s)}$	$r_3^{(s)}$	$r_4^{(s)}$	$r_5^{(s)}$	$r_6^{(s)}$	$r_7^{(s)}$	$r_8^{(s)}$	$r_9^{(s)}$	$r_{10}^{(s)}$	$r_{11}^{(s)}$	$r_{12}^{(s)}$	$r_{13}^{(s)}$	$r_{14}^{(s)}$	$r_{15}^{(s)}$	$r_{16}^{(s)}$	$r_{17}^{(s)}$	$r_{18}^{(s)}$	$r_{19}^{(s)}$	$r_{20}^{(s)}$	$r_{21}^{(s)}$	$r_{22}^{(s)}$	$r_{23}^{(s)}$	$r_{24}^{(s)}$	$r_{25}^{(s)}$	$r_{26}^{(s)}$
STAT3	1	0	0	0	0	0	0	-1	0	0	0	0	0	0	0	0	0	0	0	0	0	0	0	0	0	0
TRAF6	0	-1	0	0	0	0	0	0	0	0	0	0	0	0	0	0	0	0	0	0	0	0	0	0	0	0
P13K	0	-1	0	1	-1	-1	0	0	0	0	0	0	0	-1	0	0	-1	-1	0	0	0	0	0	0	0	0
Raf-1	0	0	-1	0	0	0	0	0	0	0	1	0	0	0	0	0	0	0	0	0	0	0	0	0	0	0
Ras	0	0	-1	-1	0	0	0	0	0	1	-1	0	0	0	0	0	0	0	-1	0	0	0	0	0	0	0
MTORC2	0	0	0	0	-1	0	0	0	0	0	0	0	0	0	0	1	0	0	0	0	0	0	0	0	0	1
AKT	0	-1	0	0	1	-1	0	0	0	0	-1	0	0	0	0	0	-1	-1	-1	0	0	0	0	0	0	0
ROS	0	0	0	0	0	-1	0	0	0	0	0	0	0	0	0	0	0	0	0	0	0	0	-1	-1	0	0
Rheb	0	0	0	0	0	-1	0	0	0	0	0	0	1	0	0	0	0	0	0	0	0	0	0	0	0	0
ERK	0	0	1	0	0	0	-1	-1	-1	0	0	0	-1	0	0	0	0	0	0	0	0	0	0	0	0	0
mTOR	0	0	0	0	0	1	0	-1	-1	0	0	0	0	0	0	0	0	0	0	0	0	0	0	0	0	0
elf4E	0	0	0	0	0	0	0	-1	1	0	0	0	0	0	0	0	0	0	0	0	0	0	0	0	0	0
NF-κB	0	1	0	0	0	0	0	-1	0	0	0	0	0	0	0	0	-1	0	0	0	0	0	0	0	0	0
MNK	0	0	0	0	0	0	1	0	-1	0	0	0	0	0	0	0	0	0	0	0	0	0	0	0	0	0
PDK1	0	0	0	0	0	0	0	0	0	0	0	0	0	1	-1	0	0	0	0	0	0	0	0	0	0	0
p53	0	0	0	0	0	0	0	0	0	0	0	0	0	0	0	0	0	0	-1	1	0	-1	0	0	-1	0
MDMX	0	0	0	0	0	0	0	0	0	0	0	0	0	0	0	0	0	0	-1	0	1	0	0	0	0	0
AMPK	0	0	0	0	0	0	0	0	0	0	0	1	-1	0	0	0	0	0	0	-1	0	0	0	0	0	0
HIF-1	0	0	0	0	0	0	0	1	0	0	0	0	0	-1	0	0	0	0	0	0	0	0	0	0	0	0
PKCs	0	0	0	0	0	0	0	0	0	0	0	0	0	0	1	0	0	0	0	0	0	0	0	0	0	0
FASL	0	0	0	0	0	0	0	0	0	0	0	0	0	0	0	0	0	1	0	0	0	0	0	0	0	0
MDM2	0	0	0	0	0	0	0	0	0	0	0	0	0	0	0	0	0	0	1	-1	-1	0	0	0	0	0
PTEN	0	0	0	-1	-1	0	0	0	0	0	0	0	0	-1	0	0	0	0	0	0	0	1	0	0	0	0
MTORC1	0	0	0	0	0	0	0	0	0	0	0	0	0	0	0	0	0	0	0	0	0	0	0	0	0	-1
TIGAR	0	0	0	0	0	0	0	0	0	0	0	0	0	0	0	0	0	0	0	0	0	0	0	0	1	0
VHL	0	0	0	0	0	0	0	0	0	0	0	0	0	0	0	0	0	0	0	0	0	0	1	0	0	0
PHD	0	0	0	0	0	0	0	0	0	0	0	0	0	0	0	0	0	0	0	0	0	0	0	1	0	0
BCLX	0	0	0	0	0	0	0	0	0	0	0	0	0	0	0	0	1	0	0	0	0	0	0	0	0	0
MYC	0	0	0	0	0	0	0	0	0	0	0	0	0	0	0	0	0	0	-1	0	0	0	0	0	0	0

	$r_{27}^{(s)}$	$r_{28}^{(s)}$	$r_{29}^{(s)}$	$r_{30}^{(s)}$	$r_{31}^{(s)}$	$r_{32}^{(s)}$	$r_{33}^{(s)}$	$r_{34}^{(s)}$	$r_{35}^{(s)}$	$r_{36}^{(s)}$	$r_{37}^{(s)}$	$r_{38}^{(s)}$	$r_{39}^{(s)}$	$r_{43}^{(s)}$	$r_{44}^{(s)}$	$r_{45}^{(s)}$	$r_{46}^{(s)}$	$r_{47}^{(s)}$	$r_{48}^{(s)}$	$r_{49}^{(s)}$	$r_{50}^{(s)}$	$r_{52}^{(s)}$	$r_{53}^{(s)}$	$r_{54}^{(s)}$
STAT3	0	0	0	0	1	0	0	0	0	0	0	0	0	0	0	0	0	0	0	0	0	0	0	0
TRAF6	0	0	0	0	0	0	0	0	0	0	0	0	0	0	0	0	0	0	1	1	0	0	0	0
P13K	0	0	0	0	0	0	0	0	0	0	1	1	0	0	0	0	0	0	0	0	0	1	0	0
Raf-1	0	0	0	0	0	0	0	0	0	0	0	0	0	0	0	0	0	0	0	0	0	0	0	0
Ras	0	0	0	0	0	0	0	0	0	0	0	0	1	1	0	0	0	0	0	0	0	0	0	0
MTORC2	-1	0	0	0	0	0	0	0	0	0	0	0	0	0	0	0	0	0	0	0	0	0	0	0
AKT	-1	0	0	0	0	0	0	0	0	0	0	0	0	0	0	0	0	0	0	0	0	0	0	0
ROS	0	1	0	0	0	0	0	0	0	0	0	0	0	0	0	0	0	0	0	0	0	0	0	0
Rheb	0	0	0	0	0	0	0	0	0	0	0	0	0	0	0	0	1	1	0	0	0	0	0	0
ERK	0	0	-1	0	0	0	0	0	0	1	0	0	0	0	0	0	0	0	0	0	0	0	0	0
mTOR	0	0	0	0	0	0	0	0	0	0	0	0	0	0	0	0	0	0	0	0	0	0	1	0
elf4E	0	0	0	0	0	0	0	0	0	0	0	0	0	0	0	0	0	0	0	0	0	0	0	0
NF-κB	0	0	0	0	0	1	1	1	1	0	0	0	0	0	0	0	0	0	0	0	0	0	0	0
MNK	0	0	0	0	0	0	0	0	0	0	0	0	0	0	0	0	0	0	0	0	0	0	0	0
PDK1	0	0	0	0	0	0	0	0	0	0	0	0	0	0	0	0	0	0	0	0	0	0	0	0
p53	0	0	0	0	0	0	0	0	0	0	0	0	0	0	0	0	0	0	0	0	1	0	0	0
MDMX	0	0	0	0	0	0	0	0	0	0	0	0	0	0	0	0	0	0	0	0	0	0	0	0
AMPK	0	0	0	0	0	0	0	0	0	0	0	0	0	0	1	1	0	0	0	0	0	0	0	0
HIF-1	0	0	0	0	0	0	0	0	0	0	0	0	0	0	0	0	0	0	0	0	0	0	0	0
PKCs	0	0	0	0	0	0	0	0	0	0	0	0	0	0	0	0	0	0	0	0	0	0	0	0
FASL	0	0	0	0	0	0	0	0	0	0	0	0	0	0	0	0	0	0	0	0	0	0	0	0
MDM2	0	0	0	0	0	0	0	0	0	0	0	0	0	0	0	0	0	0	0	0	0	0	0	0
PTEN	0	0	0	0	0	0	0	0	0	0	0	0	0	0	0	0	0	0	0	0	0	0	0	0
MTORC1	1	-1	0	0	0	0	0	0	0	0	0	0	0	0	0	0	0	0	0	0	0	0	0	0
TIGAR	0	0	0	0	0	0	0	0	0	0	0	0	0	0	0	0	0	0	0	0	0	0	0	0
VHL	0	0	0	0	0	0	0	0	0	0	0	0	0	0	0	0	0	0	0	0	0	0	0	0
PHD	0	0	0	1	0	0	0	0	0	0	0	0	0	0	0	0	0	0	0	0	0	0	0	0
BCLX	0	0	0	0	0	0	0	0	0	0	0	0	0	0	0	0	0	0	0	0	0	0	0	0
MYC	0	0	1	0	0	0	0	0	0	0	0	0	0	0	0	0	0	0	0	0	0	0	0	1

Table S-9 Initial values of different molecules involved in integrated metabolic, signaling and gene regulatory network under consideration in the main article. Here, we have generated different samples to test the validity our model with several initial values in [0,1]. However, in this table we have provided one of the sets of initial values, that we have considered.

Pathway type	Name of molecule	Initial value
**Metabolic**	Glucose-1P	0.7333
	Glucose-6P	0.7152
	Glucose	0.6143
	Fructose-6P	0.922
	Fructose-1,6BP	0.6691
	Fructose-2,6BP	0.1878
	Dihydroxyacetone phosphate	0.3506
	Glyceraldehide-3P	0.5922
	1,3-Bisphosphoglycerate	0.9618
	3-Phosphoglycerate	0.9684
	2-Phosphoglycerate	0.2419
	Phosphoenolpyruvate	0.9735
	Pyruvate	0.9615
	Lactate	0.5368
	Acetyle-CoA	0.8203
	Oxaloacetate	0.4277
	Citrate	0.4796
	Isocitrate	0.9242
	α-Ketoglutarate	0.813
	Succinyl-CoA	0.9635
	Succinate	0.6902
	Fumarate	0.2321
	Malate	0.8642
	Glucono-1,5-lactone-6P	0.9406
	Gluconate-6P	0.7109
	Ribulose-5P	0.782
	Ribose-5P	0.7688
	Xylulose-5P	0.453
	Sedoheptolose-7P	0.6899
	Erythrose-4P	0.2541
	ATP	0.7354
	ADP	0.5286
	NADH	0.3492
	NAD	0.1416
	CoA	0.1874
	CO2	0.8411
	FFA	0.7253
	FAD	0.3854

Table S-9 continued from previous page

Pathway type	Name of molecule	Initial value
	FADH	0.9552
	CoA-SH	0.131
	NADPH	0.4949
**Signaling**	STAT3	0.5316
	NF-κB	0.8212
	ERK	0.3051
	P13K	0.5483
	AKT	0.9108
	mTOR	0.6172
	MNK	0.8607
	HIF-1	0.3648
	elf4E	0.6274
	Ras	0.3221
	TRAF6	0.6998
	Raf-1	0.1751
	ROS	0.6634
	Rheb	0.6949
	PTEN	0.7568
	VHL	0.5017
	PHD	0.5841
	AMPK	0.7921
	PDK1	0.6233
	PKCs	0.9355
	BCL-X	0.6221
	FASL	0.1153
	MDM2	0.2088
	p53	0.8764
	MDMX	0.5359
	MYC	0.8604
	TIGAR	0.2885
	MTORC2	0.5971
	MTORC1	0.6669
**Gene Regulatory**	Glut 1	0.968
	Phosphoglucomutase 1	0.4129
	Hexokinase	0.735
	Glucose-6-phosphatase	0.6275
	Phosphoglucoisomerase	0.3359
	Phosphofructokinase 1	0.74
	Fructose-1,6-bisphosphatase	0.7794
	Phosphofructokinase 2	0.3185
	Fructose-2,6-bisphosphatase	0.4982

Table S-9 continued from previous page

Pathway type	Name of molecule	Initial value
	Aldolase	0.719
	Triose phosphate isomerase	0.4233
	Glyceraldehyde-3-phosphate dehydrogenase	0.7627
	Phosphoglycerate kinase	0.4552
	Phosphoglycerate mutase	0.7151
	Enolase	0.7336
	Pyruvate kinase	0.6981
	Lactate dehydrogenase	0.1176
	Pyruvate dehydrogenase	0.3978
	Acyl-CoA synthetase	0.4819
	Fatty acid synthase	0.3432
	Pyruvate carboxylase	0.8773
	Phosphoenolpyruvate carboxykinase 1	0.8395
	Citrate synthase	0.8869
	Aconitase 1	0.899
	Isocitrate dehydrogenase	0.8521
	α-Ketoglutarate dehydrogenase complex	0.7922
	Succinyl CoA synthetase	0.8971
	Succinate dehydrogenase	0.8277
	Fumarase	0.7796
	Malate dehydrogenase	0.4397
	Glucose-6-phosphate dehydrogenase	0.2944
	6-phosphogluconolactonase	0.8114
	Phosphogluconate dehydrogenase	0.9544
	Ribose 5-phosphate isomerise A	0.3948
	Ribulose-5-phosphate-3-epimerase	0.7041
	Transketolase	0.4948
	Transaldolase 1	0.8502

Table S-10 Abbreviations of different molecules used in Tables S-1, S-2, S-3, S-4, S-5, S-6, S-7 and S-8. The names of the molecules involved in the signaling pathway under consideration are same as their abbreviations.

Pathway type	Full name of molecule	Abbreviation
**Metabolic**	Glucose-1P	glc_1P
	Glucose-6P	glc_6P
	Glucose	glc
	Fructose-6P	f_6P
	Fructose-1,6BP	f_16_BP
	Fructose-2,6BP	f_26_BP
	Dihydroxyacetone phosphate	dhap

Table S-10 continued from previous page

Pathway type	Full name of molecule	Abbreviation
	Glyceraldehide-3P	ga_3P
	1,3-Bisphosphoglycerate	1,3_BPG
	3-Phosphoglycerate	PG_3
	2-Phosphoglycerate	PG_2
	Phosphoenolpyruvate	PEP
	Pyruvate	PYR
	Lactate	LACTATE
	Acetyle-CoA	Ace_coa
	Oxaloacetate	OAA
	Citrate	Citrate
	Isocitrate	Isocitrate
	α-Ketoglutarate	alpha_KG
	Succinyl-CoA	Succ_coa
	Succinate	succinate
	Fumarate	fumarate
	Malate	malate
	Glucono-1,5-lactone-6P	gl_15_l6P
	Gluconate-6P	gl_6P
	Ribulose-5P	rbls_5P
	Ribose-5P	rbs_5P
	Xylulose-5P	xyl_5P
	Sedoheptolose-7P	sdhl_7P
	Erythrose-4P	eryt_4P
	ATP	ATP
	ADP	ADP
	NADH	NADH
	NAD	NAD
	CoA	COA
	CO2	CO2
	FFA	FFA
	FAD	FAD
	FADH	FADH
	CoA-SH	COA_SH
	NADPH	NADPH
**Gene Regulatory**	Glut 1	glut1
	Phosphoglucomutase 1	pgm_1
	Hexokinase	hk
	Glucose-6-phosphatase	g6Pase
	Phosphoglucoisomerase/ Glucose 6P isomerase	pgi
	Phosphofructokinase 1	pfk1
	Fructose-1,6-bisphosphatase	f16Bpase

Table S-10 continued from previous page

Pathway type	Full name of molecule	Abbreviation
	Phosphofructokinase 2	pfk2
	Fructose-2,6-bisphosphatase	f26Bpase
	Aldolase	ald
	Triose phosphate isomerase	tpi
	Glyceraldehyde-3-phosphate dehydrogenase	g3Pdh
	Phosphoglycerate kinase	pglck
	Phosphoglycerate mutase	pglcm
	Enolase	enl
	Pyruvate kinase	pk
	Lactate dehydrogenase	ldh
	Pyruvate dehydrogenase	pyrd
	Acyl-CoA synthetase	Acyl_coa_syn
	Fatty acid synthase	fa_synthase
	Pyruvate carboxylase	pyr_crbxylase
	Phosphoenolpyruvate carboxykinase 1	pep_crbxykinase1
	Citrate synthase	cit_synthase
	Aconitase 1	actnase
	Isocitrate dehydrogenase	isocit_deh
	α-Ketoglutarate dehydrogenase complex	KG_deh_cmp
	Succinyl CoA synthetase	succ_coa_synthase
	Succinate dehydrogenase	succ_deh
	Fumarase	frmase
	Malate dehydrogenase	mal_deh
	Glucose-6-phosphate dehydrogenase	g6Pdeh
	6-phosphogluconolactonase	pglc6
	Phosphogluconate dehydrogenase	phglc_deh
	Ribose 5-phosphate isomerise A	r5Piso_A
	Ribulose-5-phosphate-3-epimerase	rbls_5P_3ep
	Transketolase	trkl
	Transaldolase 1	trsdl1

Table S-11 The external inputs under consideration in the main article.

Sl. no.	External input Name	Symbol
1	IL6	u_1
2	LPS	u_2
3	IFN_y	u_3
4	TNF	u_4
5	IL1	u_5
6	GF	u_6
7	GPCR	u_7
8	cAMP	u_8
9	LKB1	u_9
10	RORa	u_{10}
11	Wnt	u_{11}
12	ATM + ATR = (ATMATR)	u_{12}
13	PTP	u_{13}
14	MKP	u_{14}
15	G12 + NF1 + pl20GAF= GNP	u_{15}
16	E2F1	u_{16}
17	glc_ext	u_{17}
18	NADP	u_{18}
19	Insulin	u_{19}
20	Glucagon	u_{20}
21	Ca	u_{21}
22	fluoro_acetate	u_{22}
23	Carboxin (ctm)	u_{23}
24	AMP	u_{24}
25	Alanine	u_{25}
26	glc_16BP	u_{26}
27	BPG_23	u_{27}

Table S-12 Values of kinetic rate constants involved in the modelling of integrated metabolic, signaling and gene regulatory network as described in the main article. The term $\mathcal{K}_{40}^{(s)}$ - $\mathcal{K}_{42}^{(s)}$ have not been used in simulation.

Pathway type	Kinetic constant	Value
**Metabolic**	$\mathcal{K}_{1}^{(m)}$	0.09
	$\mathcal{K}_{2}^{(m)}$	0.01
	$\mathcal{K}_{3}^{(m)}$	0.09
	$\mathcal{K}_{4}^{(m)}$	0.01
	$\mathcal{K}_{5}^{(m)}$	0.0812
	$\mathcal{K}_{6}^{(m)}$	0.09
	$\mathcal{K}_{7}^{(m)}$	0.017
	$\mathcal{K}_{8}^{(m)}$	0.09
	$\mathcal{K}_{9}^{(m)}$	0.0766
	$\mathcal{K}_{10}^{(m)}$	0.0826
	$\mathcal{K}_{11}^{(m)}$	0.0899
	$\mathcal{K}_{12}^{(m)}$	0.076
	$\mathcal{K}_{13}^{(m)}$	0.0532
	$\mathcal{K}_{14}^{(m)}$	0.0755
	$\mathcal{K}_{15}^{(m)}$	0.0727
	$\mathcal{K}_{16}^{(m)}$	0.05
	$\mathcal{K}_{17}^{(m)}$	0.01
	$\mathcal{K}_{18}^{(m)}$	0.0809
	$\mathcal{K}_{19}^{(m)}$	0.0643
	$\mathcal{K}_{20}^{(m)}$	0.09
	$\mathcal{K}_{21}^{(m)}$	0.0852
	$\mathcal{K}_{22}^{(m)}$	0.0296
	$\mathcal{K}_{23}^{(m)}$	0.0818
	$\mathcal{K}_{24}^{(m)}$	0.0844
	$\mathcal{K}_{25}^{(m)}$	0.0881
	$\mathcal{K}_{26}^{(m)}$	0.0848
	$\mathcal{K}_{27}^{(m)}$	0.09
	$\mathcal{K}_{28}^{(m)}$	0.0757
	$\mathcal{K}_{29}^{(m)}$	0.0766
	$\mathcal{K}_{30}^{(m)}$	0.0752
	$\mathcal{K}_{31}^{(m)}$	0.0603
	$\mathcal{K}_{32}^{(m)}$	0.0191
	$\mathcal{K}_{33}^{(m)}$	0.0658
	$\mathcal{K}_{34}^{(m)}$	0.0792
	$\mathcal{K}_{35}^{(m)}$	0.069
	$\mathcal{K}_{36}^{(m)}$	0.0892

Table S-12 continued from previous page

Pathway type	Kinetic constant	Value
	$\mathcal{K}_{37}^{(m)}$	0.0722
**Signaling**	$\mathcal{K}_{1}^{(s)}$	0.0013
	$\mathcal{K}_{2}^{(s)}$	0.0059
	$\mathcal{K}_{3}^{(s)}$	0.0039
	$\mathcal{K}_{4}^{(s)}$	0.0014
	$\mathcal{K}_{5}^{(s)}$	0.0049
	$\mathcal{K}_{6}^{(s)}$	0.0025
	$\mathcal{K}_{7}^{(s)}$	0.002
	$\mathcal{K}_{8}^{(s)}$	0.0016
	$\mathcal{K}_{9}^{(s)}$	0.0022
	$\mathcal{K}_{10}^{(s)}$	0.0025
	$\mathcal{K}_{11}^{(s)}$	0.0013
	$\mathcal{K}_{12}^{(s)}$	0.0061
	$\mathcal{K}_{13}^{(s)}$	0.0033
	$\mathcal{K}_{14}^{(s)}$	0.0053
	$\mathcal{K}_{15}^{(s)}$	0.0066
	$\mathcal{K}_{16}^{(s)}$	0.005
	$\mathcal{K}_{17}^{(s)}$	0.0053
	$\mathcal{K}_{18}^{(s)}$	0.0046
	$\mathcal{K}_{19}^{(s)}$	0.002
	$\mathcal{K}_{20}^{(s)}$	0.0049
	$\mathcal{K}_{21}^{(s)}$	0.0078
	$\mathcal{K}_{22}^{(s)}$	0.008
	$\mathcal{K}_{23}^{(s)}$	0.0032
	$\mathcal{K}_{24}^{(s)}$	0.0027
	$\mathcal{K}_{25}^{(s)}$	0.0055
	$\mathcal{K}_{26}^{(s)}$	0.0061
	$\mathcal{K}_{27}^{(s)}$	0.0043
	$\mathcal{K}_{28}^{(s)}$	0.0026
	$\mathcal{K}_{29}^{(s)}$	0.0086
	$\mathcal{K}_{31}^{(s)}$	0.0018
	$\mathcal{K}_{32}^{(s)}$	0.0021
	$\mathcal{K}_{33}^{(s)}$	0.0023
	$\mathcal{K}_{34}^{(s)}$	0.006
	$\mathcal{K}_{35}^{(s)}$	0.0056
	$\mathcal{K}_{36}^{(s)}$	0.0014
	$\mathcal{K}_{37}^{(s)}$	0.0084
	$\mathcal{K}_{38}^{(s)}$	0.0068
	$\mathcal{K}_{39}^{(s)}$	0.0069

Table S-12 continued from previous page

Pathway type	Kinetic constant	Value
	$\mathcal{K}_{43}^{(s)}$	0.0015
	$\mathcal{K}_{44}^{(s)}$	0.0079
	$\mathcal{K}_{45}^{(s)}$	0.0085
	$\mathcal{K}_{46}^{(s)}$	0.0089
	$\mathcal{K}_{47}^{(s)}$	0.0079
	$\mathcal{K}_{48}^{(s)}$	0.0073
	$\mathcal{K}_{49}^{(s)}$	0.0051
	$\mathcal{K}_{50}^{(s)}$	0.0024
	$\mathcal{K}_{52}^{(s)}$	0.0042
	$\mathcal{K}_{53}^{(s)}$	0.0021
	$\mathcal{K}_{54}^{(s)}$	0.0012
**Gene Regulatory**	$\mathcal{K}_{1}^{(g)}$	0.0007405
	$\mathcal{K}_{2}^{(g)}$	0.0008736
	$\mathcal{K}_{3}^{(g)}$	0.0006312
	$\mathcal{K}_{4}^{(g)}$	0.0008237
	$\mathcal{K}_{5}^{(g)}$	0.0008209
	$\mathcal{K}_{6}^{(g)}$	0.0007686
	$\mathcal{K}_{7}^{(g)}$	0.0006553
	$\mathcal{K}_{8}^{(g)}$	0.0007792
	$\mathcal{K}_{9}^{(g)}$	0.00069
	$\mathcal{K}_{10}^{(g)}$	0.0006402
	$\mathcal{K}_{11}^{(g)}$	0.0006638
	$\mathcal{K}_{12}^{(g)}$	0.0008685
	$\mathcal{K}_{13}^{(g)}$	0.0006214
	$\mathcal{K}_{14}^{(g)}$	0.0006727
	$\mathcal{K}_{15}^{(g)}$	0.0006161
	$\mathcal{K}_{16}^{(g)}$	0.0007325
	$\mathcal{K}_{17}^{(g)}$	0.000604
	$\mathcal{K}_{18}^{(g)}$	0.0008692
	$\mathcal{K}_{19}^{(g)}$	0.000659
	$\mathcal{K}_{20}^{(g)}$	0.000628
	$\mathcal{K}_{21}^{(g)}$	0.0006922
	$\mathcal{K}_{22}^{(g)}$	0.0007368
	$\mathcal{K}_{23}^{(g)}$	0.0006305
	$\mathcal{K}_{24}^{(g)}$	0.0008986
	$\mathcal{K}_{25}^{(g)}$	0.0006996
	$\mathcal{K}_{26}^{(g)}$	0.0006892
	$\mathcal{K}_{27}^{(g)}$	0.0006186

Table S-12 continued from previous page

Pathway type	Kinetic constant	Value
	$\mathcal{K}_{28}^{(g)}$	0.0006895
	$\mathcal{K}_{29}^{(g)}$	0.0006139
	$\mathcal{K}_{30}^{(g)}$	0.0007516
	$\mathcal{K}_{31}^{(g)}$	0.0008284
	$\mathcal{K}_{32}^{(g)}$	0.0007893
	$\mathcal{K}_{33}^{(g)}$	0.000627
	$\mathcal{K}_{34}^{(g)}$	0.0006243
	$\mathcal{K}_{35}^{(g)}$	0.0008332
	$\mathcal{K}_{36}^{(g)}$	0.0008715
	$\mathcal{K}_{37}^{(g)}$	0.0007601

Table S-13 Values of basal production rate constants for different genes involved in the gene regulatory network (GRN) under consideration in the main article.

GRN basal production rate constant	Value
b_{glut1}	0.0004939
b_{pgm_1}	0.0004301
b_{hk}	0.0004296
b_{g6Pase}	0.0004333
b_{pgi}	0.0004467
b_{pfk1}	0.0004648
$b_{f16Bpase}$	0.0004025
b_{pfk2}	0.0004842
$b_{f26Bpase}$	0.0004559
b_{ald}	0.0004854
b_{tpi}	0.0004348
b_{g3Pdh}	0.0004446
b_{pglck}	0.0004054
b_{pglcm}	0.0004177
b_{enl}	0.0004663
b_{pk}	0.0004331
b_{ldh}	0.0004898
b_{pyrd}	0.0004118
$b_{acyl\_coa\_syn}$	0.0004988
$b_{fa\_synthase}$	0.000454
$b_{pyr\_crbxylase}$	0.0004707
$b_{pep\_crbxykinase1}$	0.0004999
$b_{cit\_synthase}$	0.0004288
$b_{actnase}$	0.0004415
b_{isocit_deh}	0.0004465
$b_{KG\_deh\_cmp}$	0.0004764
$b_{succ\_coa\_synthase}$	0.0004818
b_{succ_deh}	0.00041
b_{frmase}	0.0004178
b_{mal_deh}	0.000436
b_{g6P_deh}	0.0004057
b_{pglc6}	0.0004522
b_{phglc_deh}	0.0004336
b_{r5Piso_A}	0.0004176
$b_{rbls\_5P\_3ep}$	0.0004209
b_{trkl}	0.0004905
b_{trsdl_1}	0.0004675

Table S-14 Values of decay rate constants for different genes involved in the gene regulatory network (GRN) under consideration in the main article. Here, it should be mentioned that values of all feedback/binding constants under consideration in the main article have been taken as 0.9.

GRN decay rate constant	Value
d_{glut1}	0.0001109
d_{pgm_1}	0.0001826
d_{hk}	0.0001338
d_{g6Pase}	0.0001294
d_{pgi}	0.0001746
d_{pfk1}	0.000101
$d_{f16Bpase}$	0.0001048
d_{pfk2}	0.0001668
$d_{f26Bpase}$	0.0001603
d_{ald}	0.0001526
d_{tpi}	0.000173
d_{g3Pdh}	0.0001707
d_{pglck}	0.0001781
d_{pglcm}	0.0001288
d_{enl}	0.0001693
d_{pk}	0.0001557
d_{ldh}	0.0001397
d_{pyrd}	0.0001062
$d_{acyl\_coa\_syn}$	0.000178
$d_{fa\_synthase}$	0.0001338
$d_{pyr\_crbxylase}$	0.0001608
$d_{pep\_crbxykinase1}$	0.0001741
$d_{cit\_synthase}$	0.0001105
$d_{actnase}$	0.0001128
d_{isocit_deh}	0.000155
$d_{KG\_deh\_cmp}$	0.0001485
$d_{succ\_coa\_synthase}$	0.000189
d_{succ_deh}	0.0001799
d_{frmase}	0.0001734
d_{mal_deh}	0.0001051
d_{g6P_deh}	0.0001073
d_{pglc6}	0.0001089
d_{phglc_deh}	0.0001798
d_{r5Piso_A}	0.0001943
$d_{rbls\_5P\_3ep}$	0.0001684
d_{trkl}	0.0001132
d_{trsdl_1}	0.0001723

Table S-15 Values of Michaelis Menten constants under consideration in the main article.

**Michaelis Menten constant values for metabolic pathway**	
$\mathcal{K}_{0}^{\mathcal{M}^{(m)}}$	0.05564
$\mathcal{K}_{1}^{\mathcal{M}^{(m)}}$	0.4479
$\mathcal{K}_{2}^{\mathcal{M}^{(m)}}$	0.05966
$\mathcal{K}_{3}^{\mathcal{M}^{(m)}}$	0.052076
$\mathcal{K}_{4}^{\mathcal{M}^{(m)}}$	0.4479
$\mathcal{K}_{5}^{\mathcal{M}^{(m)}}$	0.052373
$\mathcal{K}_{6}^{\mathcal{M}^{(m)}}$	0.4289
$\mathcal{K}_{7}^{\mathcal{M}^{(m)}}$	0.054248
$\mathcal{K}_{8}^{\mathcal{M}^{(m)}}$	0.4654
$\mathcal{K}_{9}^{\mathcal{M}^{(m)}}$	0.053261
$\mathcal{K}_{10}^{\mathcal{M}^{(m)}}$	0.07412
$\mathcal{K}_{11}^{\mathcal{M}^{(m)}}$	0.052933
$\mathcal{K}_{12}^{\mathcal{M}^{(m)}}$	0.4415
$\mathcal{K}_{13}^{\mathcal{M}^{(m)}}$	0.09692
$\mathcal{K}_{14}^{\mathcal{M}^{(m)}}$	0.41931
$\mathcal{K}_{15}^{\mathcal{M}^{(m)}}$	0.051036
$\mathcal{K}_{16}^{\mathcal{M}^{(m)}}$	0.4729
$\mathcal{K}_{17}^{\mathcal{M}^{(m)}}$	0.13405
$\mathcal{K}_{18}^{\mathcal{M}^{(m)}}$	0.4658
$\mathcal{K}_{19}^{\mathcal{M}^{(m)}}$	0.053377
$\mathcal{K}_{20}^{\mathcal{M}^{(m)}}$	0.3951
$\mathcal{K}_{21}^{\mathcal{M}^{(m)}}$	0.073413
$\mathcal{K}_{22}^{\mathcal{M}^{(m)}}$	0.42947
$\mathcal{K}_{23}^{\mathcal{M}^{(m)}}$	0.13745
$\mathcal{K}_{24}^{\mathcal{M}^{(m)}}$	0.49851
$\mathcal{K}_{25}^{\mathcal{M}^{(m)}}$	0.054972
$\mathcal{K}_{26}^{\mathcal{M}^{(m)}}$	0.45491484
$\mathcal{K}_{27}^{\mathcal{M}^{(m)}}$	0.1576
$\mathcal{K}_{28}^{\mathcal{M}^{(m)}}$	0.074994
$\mathcal{K}_{29}^{\mathcal{M}^{(m)}}$	0.4786
$\mathcal{K}_{30}^{\mathcal{M}^{(m)}}$	0.052321
$\mathcal{K}_{31}^{\mathcal{M}^{(m)}}$	0.052518
$\mathcal{K}_{32}^{\mathcal{M}^{(m)}}$	0.42146
$\mathcal{K}_{33}^{\mathcal{M}^{(m)}}$	0.053936
$\mathcal{K}_{34}^{\mathcal{M}^{(m)}}$	0.063326
$\mathcal{K}_{35}^{\mathcal{M}^{(m)}}$	0.050974
$\mathcal{K}_{36}^{\mathcal{M}^{(m)}}$	0.4698
$\mathcal{K}_{37}^{\mathcal{M}^{(m)}}$	0.054877
$\mathcal{K}_{38}^{\mathcal{M}^{(m)}}$	0.051364
$\mathcal{K}_{39}^{\mathcal{M}^{(m)}}$	0.051125
$\mathcal{K}_{40}^{\mathcal{M}^{(m)}}$	0.093633
$\mathcal{K}_{41}^{\mathcal{M}^{(m)}}$	0.4922
$\mathcal{K}_{42}^{\mathcal{M}^{(m)}}$	0.062864
$\mathcal{K}_{43}^{\mathcal{M}^{(m)}}$	0.052887
$\mathcal{K}_{44}^{\mathcal{M}^{(m)}}$	0.084375
$\mathcal{K}_{45}^{\mathcal{M}^{(m)}}$	0.092682
$\mathcal{K}_{46}^{\mathcal{M}^{(m)}}$	0.052271
$\mathcal{K}_{47}^{\mathcal{M}^{(m)}}$	0.073521
$\mathcal{K}_{48}^{\mathcal{M}^{(m)}}$	0.063836

B Supplementary Figures

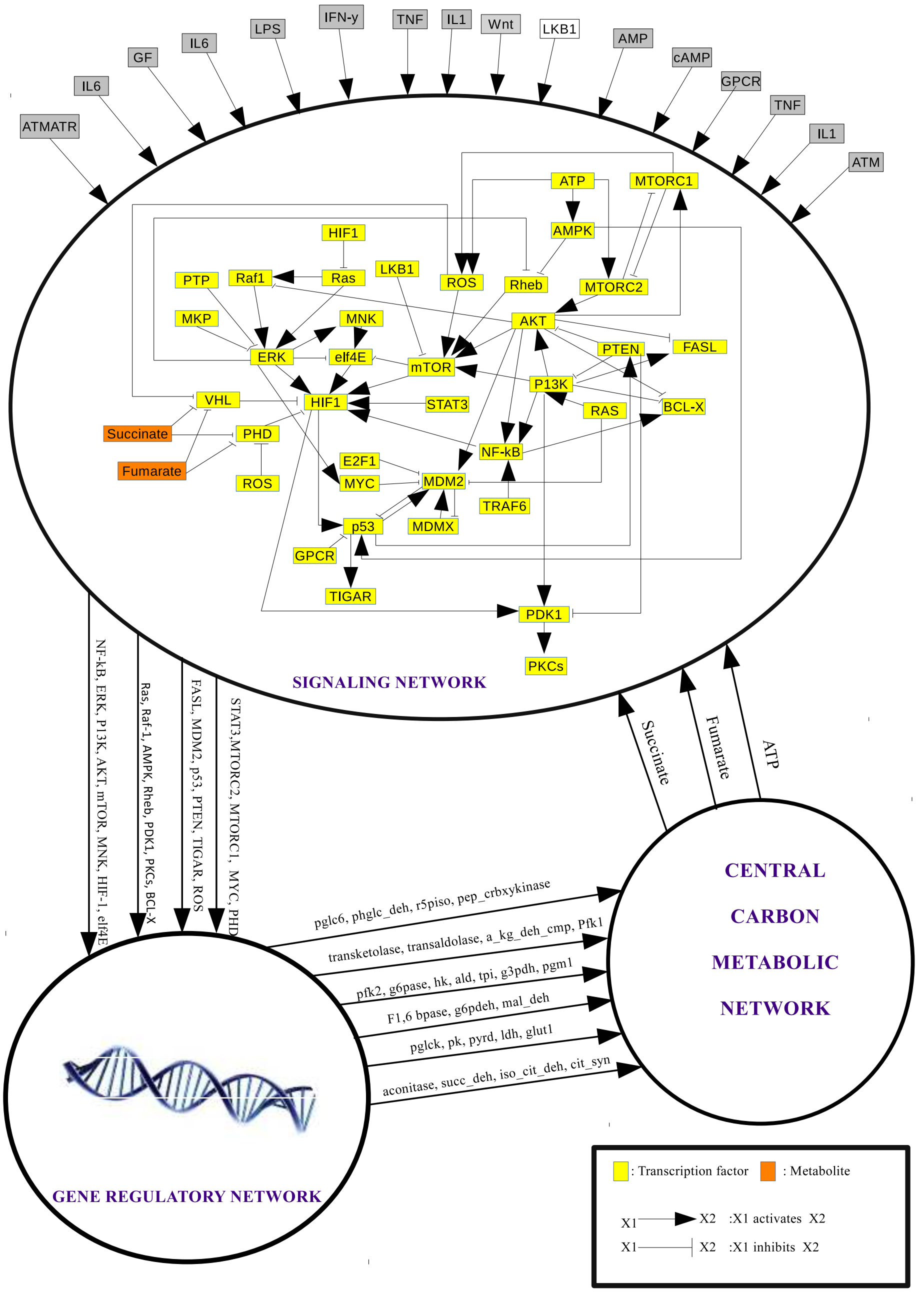

Figure S-1 Block diagram of the interactions among metabolic, signaling and gene regulatory networks related to central carbon metabolism of mammalian cells.

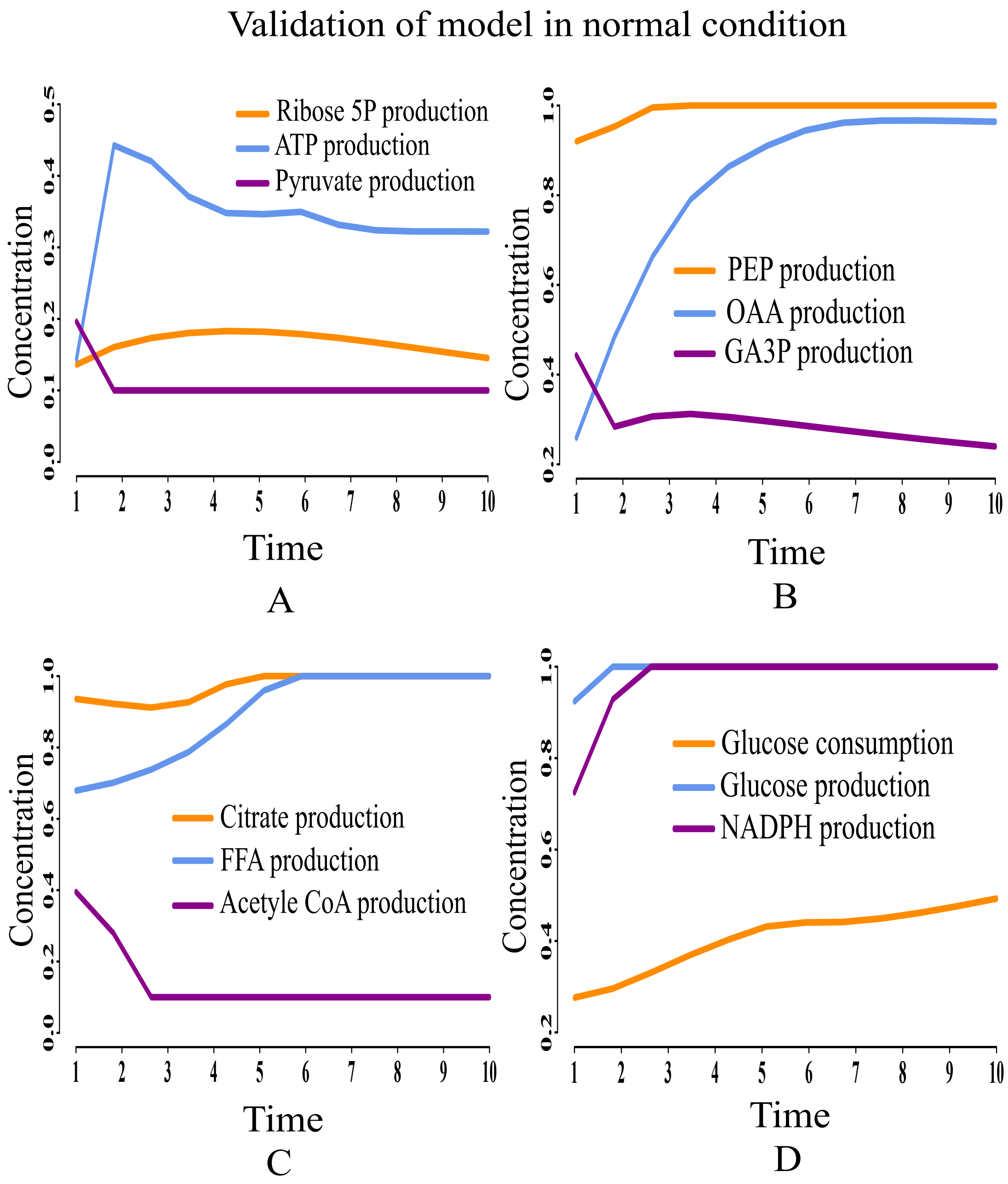

Figure S-2 Variation of concentrations of ribose 5P, ATP, pyruvate, PEP, OAA, GA3P (glyceraldehyde-3P), citrate, FFA, acetyl CoA and NADPH along with glucose consumption and production over time to validate the normal behavior of the integrated biochemical pathway related to carbon metabolism of mammalian cells.

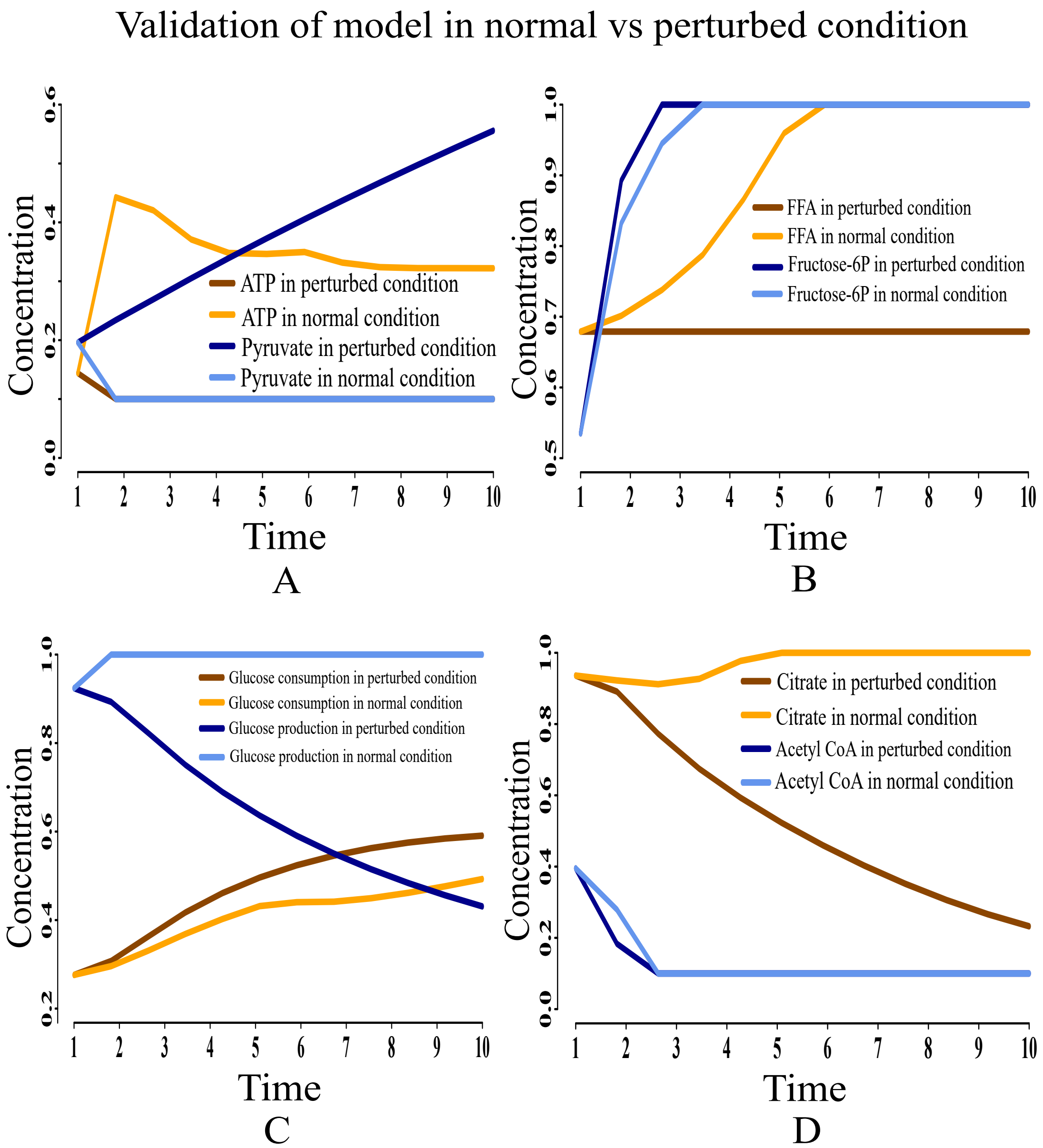

Figure S-3 Change of concentrations of ATP, pyruvate, FFA, fructose 6P, citrate, acetyl CoA over time along with glucose consumption and production in the integrated biochemical pathway related to carbon metabolism in perturbed condition compared to that in normal mammalian cells.

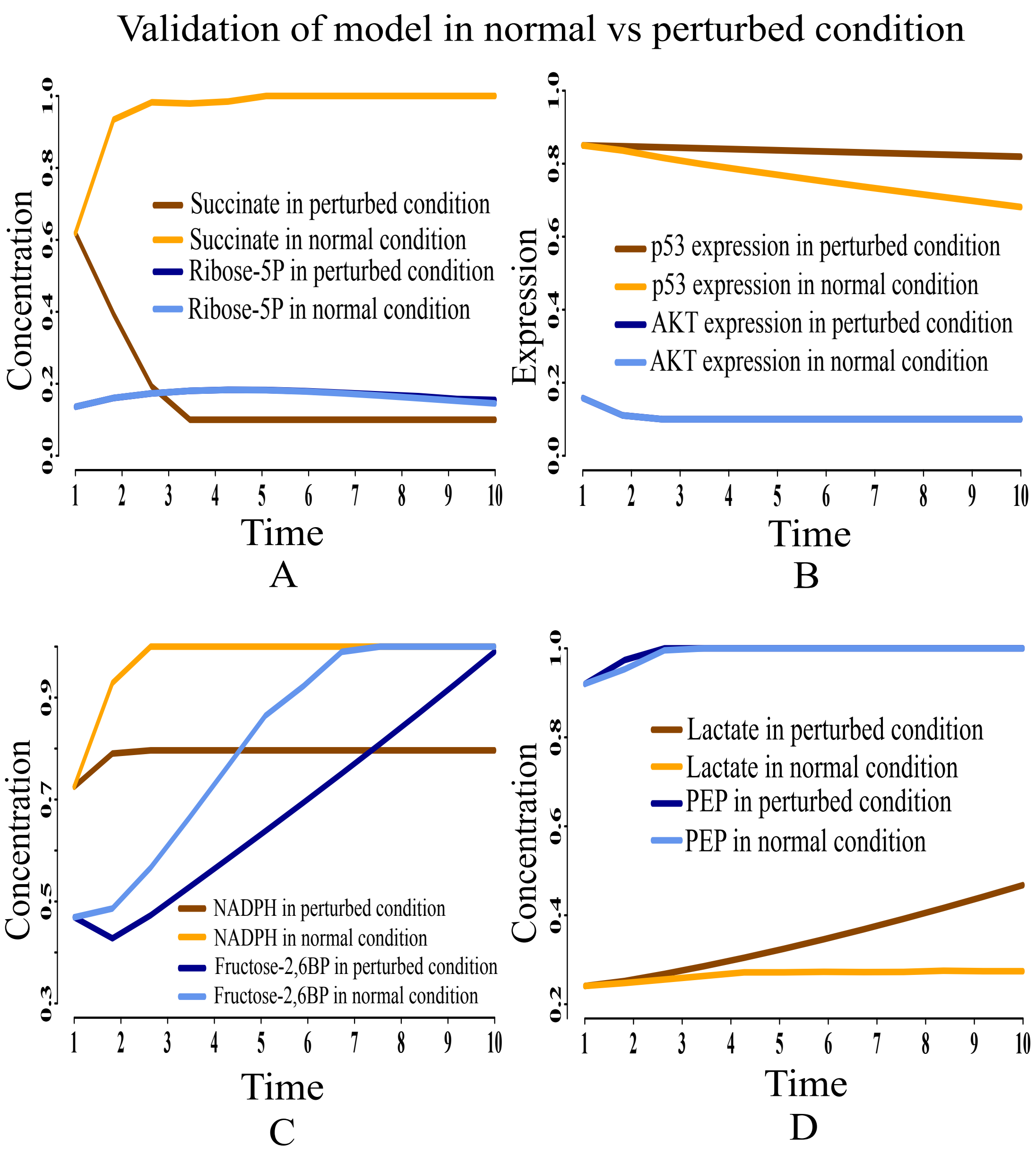

Figure S-4 Change of concentrations/expressions of succinate, ribose 5P, p53, AKT, NADPH, fructose 2,6 bisphosphate, lactate and PEP participating in the integrated biochemical pathway related to carbon metabolism in perturbed condition compared to that in normal mammalian cells.

C Supplementary Algorithms

Algorithm S-1 GA Controller Algorithm: Assuming there are **C** chromosomes (possible number of solution sets) in a population and **G** generations. Here **C** contains input-output variables. We have applied exceptional handling to find real valued solution for the output of the algorithm.

Input: $\langle \mathbf{x}, \mathbf{y}, \mathbf{z}, \mathbf{u} \rangle^{(in)}_{current}$, $\mathbf{v}_{current}$
Output: $\langle \mathbf{x}, \mathbf{y}, \mathbf{z}, \mathbf{u} \rangle^{(in)}_{new}$, $fitness^{max}$

$fitness^{max} \leftarrow 0$
$limit \leftarrow 0.1$
Population $\leftarrow$ **Create_Random_Population**($\langle \mathbf{x}, \mathbf{y}, \mathbf{z}, \mathbf{u} \rangle^{(in)}_{current}$)
for gen = 1 **to G do**
  for solution = 1 **to C do**
    $fitness_{val}$ $\leftarrow$**Calculate_Fitness**(solution, $\mathbf{v}_{current}$)
    $weighted_{pop}.append$ $\leftarrow$ **Make_Pair**(solution,$fitness_{val}$)
  end for
  Population$\leftarrow$Null
  for j = 1 **to C**/2 **do**
    $solution_1, solution_2$ $\leftarrow$ **Selection**($weighted_{pop}$)
    $solution_1^{modified}, solution_2^{modified}$ $\leftarrow$ **Crossover**($solution_1, solution_2$)
    Population.add $\leftarrow$ **Mutation**($solution_1^{modified}$)
    Population.add $\leftarrow$ **Mutation**($solution_2^{modified}$)
  end for
  $Fittest_{solution}$,$fitness^{max}$ $\leftarrow$ **Find_Fittest_Sol**(Population)
  $\langle \mathbf{x}, \mathbf{y}, \mathbf{z}, \mathbf{u} \rangle^{(in)}_{new} \leftarrow Fittest_{solution}$
  if ($1/fitness^{max} < limit$) **then**
    break
  else
    continue
  end if
end for
return $\langle \mathbf{x}, \mathbf{y}, \mathbf{z}, \mathbf{u} \rangle^{(in)}_{new}$, $fitness^{max}$

Algorithm S-2 Controlling the outputs at some specific values using GA controller.

Input: Trained SVR model, MIMO Plant model, initial input $\mathbf{v}_{current}$ and reference input $\langle \mathbf{x}, \mathbf{y}, \mathbf{z} \rangle^{(out)}_{reference}$.

Output: Final optimal input $\langle \mathbf{x}, \mathbf{y}, \mathbf{z}, \mathbf{u} \rangle^{(in)}_{final}$

tolerance $\leftarrow$ 0.01
threshold $\leftarrow$ 0.01
$fitness^{previous} \leftarrow 0$
limit $\leftarrow$ 30
count $\leftarrow$ 0
flag $\leftarrow$ 0
Generate $\langle \mathbf{x}, \mathbf{y}, \mathbf{z}, \mathbf{u} \rangle^{(in)}_{current}$ from $\mathbf{v}_{current}$
for i = 1 **to** MAXIMUM_ITERATION **do**
    $\langle \mathbf{x}, \mathbf{y}, \mathbf{z}, \mathbf{u} \rangle^{(in)}_{new}$, $fitness^{current} \leftarrow$ **GA-controller**($\langle \mathbf{x}, \mathbf{y}, \mathbf{z}, \mathbf{u} \rangle^{(in)}_{current}$, $\mathbf{v}_{current}$)
    if ((i $\geq$ 2) and ($fitness^{previous}$ - $fitness^{current}$ $>$ tolerance)) **then**
        if count $>$ limit **then**
            flag $\leftarrow$ 1
        else
            count $\leftarrow$ count + 1
            flag $\leftarrow$ 0
            Continue loop with $fitness^{previous}$ and $\langle \mathbf{x}, \mathbf{y}, \mathbf{z}, \mathbf{u} \rangle^{(in)}_{current}$
        end if
    else
        $fitness^{previous} \leftarrow fitness^{current}$
        flag $\leftarrow$ 1
    end if
    if flag = 1 **then**
        Create $\mathbf{v}_{new}$ from $\langle \mathbf{x}, \mathbf{y}, \mathbf{z}, \mathbf{u} \rangle^{(in)}_{new}$
        $\mathbf{v}_{current} \leftarrow \mathbf{v}_{new}$
        $fitness^{previous} \leftarrow fitness^{current}$
        $Y_{prediction} \leftarrow$ **SVR_Model_Predction**($\mathbf{v}_{new}$)
        $Y_{actual} \leftarrow$ **Plant_Output_Predction**($\langle \mathbf{x}, \mathbf{y}, \mathbf{z}, \mathbf{u} \rangle^{(in)}_{new}$)
        if count $>$ limit **then**
            print "Interrupt optimizing due to stuck at lower fitness solution...!!"
            break
        end if
        if ($\langle \mathbf{x}, \mathbf{y}, \mathbf{z} \rangle^{(out)}_{reference}$ - $\langle \mathbf{x}, \mathbf{y}, \mathbf{z} \rangle^{(out)}$ $<$ threshold) **then**
            $\langle \mathbf{x}, \mathbf{y}, \mathbf{z}, \mathbf{u} \rangle^{(in)}_{final} \leftarrow \langle \mathbf{x}, \mathbf{y}, \mathbf{z}, \mathbf{u} \rangle^{(in)}_{new}$
            break
        end if
    end if
end for
return $\langle \mathbf{x}, \mathbf{y}, \mathbf{z}, \mathbf{u} \rangle^{(in)}_{final}$
